# Similarities and differences between eating disorders and obsessive-compulsive disorder in childhood and adolescence: a systematic review

**DOI:** 10.3389/fpsyt.2024.1407872

**Published:** 2024-06-04

**Authors:** Michelangelo Di Luzio, Domenica Bellantoni, Anna Laura Bellantoni, Valeria Villani, Cristina Di Vincenzo, Valeria Zanna, Stefano Vicari, Maria Pontillo

**Affiliations:** ^1^ Child and Adolescent Neuropsychiatry Unit, Bambino Gesù Children’s Hospital, IRCCS, Rome, Italy; ^2^ Life Sciences and Public Health Department, Catholic University, Rome, Italy

**Keywords:** eating disorders, obsessive-compulsive disorder, childhood, adolescence, comorbidity, clinical features

## Abstract

**Background:**

The developmental age, comprising childhood and adolescence, constitutes an extremely important phase of neurodevelopment during which various psychiatric disorders can emerge. Obsessive-Compulsive Disorder (OCD) and Eating Disorders (ED) often manifest during this critical developmental period sharing similarities but also differences in psychopathology, neurobiology, and etiopathogenesis. The aim of this study is to focus on clinical, genetic and neurobiological similarities and differences in OCD and ED.

**Methods:**

This study is based on a PubMed/MEDLINE and Cochrane Central Register for Controlled Trial (CENTRAL). The research adhered to the guidelines outlined in the Preferred Reporting Items for Systematic Reviews and Meta-Analyses (PRISMA).

**Results:**

The aforementioned search yielded an initial collection of 335 articles, published from 1968 to September 2023. Through the application of inclusion and exclusion criteria, a total of 324 articles were excluded, culminating in a final selection of 10 articles.

**Conclusions:**

Our findings showed both differences and similarities between OCD and ED. Obsessive-compulsive (OC) symptoms are more prevalent in ED characterized by a binge/purge profile than in those with a restrictive profile during developmental age. OC symptomatology appears to be a common dimension in both OCD and ED. When presents, OC symptomatology, exhibits transversal characteristic alterations in the anterior cingulate cortex and poorer cognitive flexibility. These correlations could be highlighted by genetic overlaps between disorders. A comprehensive definition, integrating psychopathological and neurobiological aspects could significantly aid treatment selection and thereby influence the prognosis of these patients.

## 1 Introduction

The developmental age, comprising childhood and adolescence, constitutes an extremely important phase of neurodevelopment during which various psychiatric disorders can emerge. Obsessive-Compulsive Disorder (OCD) and Eating Disorders (ED) often manifest during this critical developmental period (1) sharing similarities but also distinctions in psychopathology, neurobiology, and etiopathogenesis. Specifically, the obsessive-compulsive (OC) symptomatology evident in ED, usually focusing on food and body shape themes, appears to be a connecting factor between the two disorders, resulting in high rates of comorbidity (2).

OCD, as defined by the Diagnostic and Statistical Manual of Mental Disorders, 5th edition text revision (3), is characterized by obsessions, compulsions, or both. Obsessions involve unwanted, repetitive, and distressing thoughts or images, often accompanied by compulsions, which are behaviors or mental actions the individual feels compelled to perform (3). One criterion of the DSM-5-TR is that obsessions and compulsions should not revolve around food, eating, or body shape, distinguishing it from ED. When OC symptomatology encompasses these themes, it can be regarded as ED-related OC symptomatology. The OCD symptoms can often be chronic, significantly impairing an individual’s functioning, especially when accompanied by poor insight and a substantial amount of time dedicated to OC rituals (4–6). OCD often emerges in late adolescence and early adulthood but can also onset in childhood (1, 7). Typically, it shows no gender differences, except in children where it predominantly affects males (8).

ED are psychiatric disorders characterized by disturbances in eating and the relationship with weight and body shape. They include Anorexia Nervosa (AN), Bulimia Nervosa (BN), Binge Eating Disorder (BED), Pica, Avoidant/Restrictive Food Intake Disorder (ARFID), and Rumination Disorder (3). They usually debut in adolescence and early adulthood (9, 10), with AN showing a progressive decrease in age of onset in recent years, including a higher prevalence in childhood (11). Scientific literature, exploring the correlation between ED and OCD, tends to focus primarily on three main ED: AN, BN, and BED. Furthermore, these three disorders exhibit similarities on a psychopathological level among them compared to the remaining three. AN, BN, and BED entail thoughts and behaviors focused on weight, body shape, and food in a consistent and repetitive manner, significantly impacting the self-esteem and mood of the individuals afflicted by them. Consequently, this review will specifically examine these three ED. AN is characterized by an intense fear of gaining weight and is associated with distorted body image. Patients with AN engage in behaviors to avoid eating and lose weight, such as elimination behaviors (exercise or vomiting/laxative use), leading to being underweight as calculated by the Body Mass Index (BMI). In BN, there is also an altered relationship with one’s body and body image, translating into repeated cycles of binge eating (consumption of a large amount of food in a short time) and subsequent compensatory or elimination behaviors. In BN, weight is maintained within normal limits or increased. BED is characterized by binge eating episodes without specific compensatory behaviors, as seen in BN, and is often associated with overweight or obesity.

As mentioned earlier, OCD and ED exhibit high comorbidity, especially with AN and BN. Specifically, in general population, BN shows a higher lifetime comorbidity with OCD in community studies (14–17%), while AN is higher in clinical populations (0–69%). This could be due to the low prevalence of AN in the general population, AN greater severity, and the scarcity of data on AN in community studies (2). Similarly, in clinical populations, the lifetime prevalence of AN in OCD (3–17%) is higher than that of BN (3–10%) (2). The presence of comorbidity usually indicates greater severity compared to the disorder alone. For example, ED with comorbid OCD show more severe ED symptoms and higher rates of hospitalization (12, 13). Moreover, OCD is sometimes identified as a risk factor for the later development of ED (2, 14), especially in a study by Micali et al. (15), suggesting that the presence of OCD in childhood and adolescence is associated with the development of ED in adulthood (15). Some longitudinal and familial studies suggest possible common etiological factors (2). Additionally, from a genetic perspective, common aspects have emerged. Although different genes have been identified, genome and twin studies confirm genetic affinity among OCD, ED, major depression, anxiety disorders, and substance use disorders (16). Furthermore, affinity between AN and OCD has been observed in Genome-Wide Association Studies (17), specifically for genes corresponding to the prefrontal cortex and serotoninergic network, indicating the presence of similar altered pathways (16, 18, 19). The implicated brain circuits in the two disorders partially overlap. These brain networks involve connections among various prefrontal cortex areas, including the anterior cingulate cortex (ACC), and the striatal region (20). The partial overlap of the neurocircuits involved in the disorders is also evident when investigating neurotransmitter aspects. It emerges that both OCD and ED show a pharmacological response to selective serotonin reuptake inhibitors (SSRIs), indicating a common alteration in the serotonin system (21–24).

Beyond epidemiological and neurobiological aspects, psychopathological characteristics also unite OCD and ED. Both disorders feature intrusive and compelling thoughts on a particular theme, leading to psychological distress and, consequently, behaviors aimed at reducing the state of distress. Based on these characteristics, some authors have hypothesized that ED and OCD may belong to the same psychopathological spectrum (2, 25–28). Other authors support the idea that ED may fall under an OC spectrum (29, 30), particularly in association with certain types of obsessions-compulsions, such as symmetry, aggression (31, 32), and cleanliness/contamination (33), and certain personality traits like perfectionism and impulsivity (34–36).

The aim of this study is to analyze these characteristics, similarities, and differences between ED and OCD specifically in children and adolescents, a less studied population. Studies focusing solely on epidemiological analyses of OCD and ED have been excluded from this review. These studies generally show overlapping data with those on adults with ED, indicating high comorbidity with OCD (37–39). Beyond comorbidity data, our study specifically aims to focus on the clinical, genetic, and neurobiological similarities and differences in OCD and ED. For this aim, studies presenting both disorders separately and in comorbidity have been included. Studies where individuals with OCD or ED exhibit traits of the other disorder without clear comorbidity were also considered.

Studying disorders at early-onset can provide crucial insights into etiology, characteristics, and potential treatments. Especially in disorders that show significant overlap in adulthood and where the childhood and adolescence are pivotal phases. Considering the literature on OCD and ED in adulthood, it is conceivable that also OCD and ED in developmental age may exhibit points of convergence and divergence. We hypothesize that OCD may have overlapping features with BN and AN, especially AN with restrictive symptoms. However, it could be suggested that ED do not strictly belong to an OC spectrum, as some authors assumed. Instead, there may be a shared OC dimension between OCD and ED, while each disorder still retains specific unique characteristics. Moreover, this hypothesis should be supported by evidence not only in the psychopathology but also in the neurobiological and genetic domains.

## 2 Methods

### 2.1 Search strategy

This study is based on a PubMed/MEDLINE and Cochrane Central Register for Controlled Trial (CENTRAL) search for studies published from the beginning of the databases until November 30, 2023, employing the following search terms: (“eating disorder” OR “anorexia nervosa” OR “binge eating disorder” OR “pica” OR “avoidant restrictive food intake disorder” OR “rumination disorder”) AND “obsessive-compulsive disorder” AND (“childhood” OR “child” OR “adolescence” OR “adolescent”). The entire research team reached a consensus on the search approach and collectively contributed to the examination of the literature. The chosen articles fulfilled the subsequent eligibility criteria (1): They constituted original research studies (2); they included subjects with a diagnosis of ED or OCD as assessed by Structured Clinical Interview for Diagnostic and statistical manual of mental disorders third/fourth/fifth edition/fifth edition text revision (DSM- III/IV/5/5-TR) or the Classification of Diseases ninth/tenth edition (ICD-9/10) (3); they compared ED to OCD patients in clinical, genetic or neurobiological features and vice versa. Otherwise they assessed clinical, genetic or neurobiological characteristics in OCD patients with ED comorbidity or symptoms or in ED patients with OCD comorbidity or symptoms (4); they separated data for ED and OCD (5); they included subjects with less than 18 years old.

### 2.2 Eligibility and study selection

The subsequent studies were not considered (1): reviews and meta-analyses (nevertheless, the reference lists of these studies were scrutinized to identify potentially eligible studies that might have been missed during the initial database search.) (i.e., “Review”) (2); case reports or case series (i.e., “Case Report”) (3); studies that did not assess individuals with OCD or ED with OCD comorbidity/symptoms (i.e., “No OCD”) (4); studies that did not assess individuals with ED or OCD with ED comorbidity/symptoms (i.e.”No ED”) (5); qualitative studies not supported by statistical analysis (i.e., “No Data”) (6); research that did not offer distinct data for OCD, ED, healthy controls or individuals with different psychiatric diagnoses (i.e., “Lumping) (7); studies unrelated to the pertinent topic (i.e., “Unrelated”) (8); studied that assessed animals (i.e., “Animal”) (9); protocols and ongoing studies (10); correction to existing article (11); studies for which no English translation was available (i.e., “No English”) (12); studies that included subjects over 18 years old (i.e., “Adult). Duplicate records in the two databases were removed. The criteria for including and excluding studies, established through two rounds of the Delphi method, gained unanimous acceptance from all authors. The research adhered to the guidelines outlined in the Preferred Reporting Items for Systematic Reviews and Meta-Analyses (PRISMA) (40). The online **Supplementary Materials** comprise the PRISMA flowchart and checklist, along with comprehensive results and data regarding included/excluded studies (refer to Online Supplement, **Supplementary Figures 1**, **2**, and **Supplementary Table 1**).

### 2.3 Data extraction and synthesis

Information extracted from the chosen articles were systematically recorded in a standardized spreadsheet. Precisely, the subsequent variables were recorded: primary author, publication year, sample size, participant age, gender ratio (male/female), study design (incorporating interviews, tests, tools or questionnaire employed), and outcomes pertaining to key clinical characteristics among individuals diagnosed with ED and OCD. The summary of included studies was reported in **Table 1**.

**Table 1:** Summary of included studies.

Study	Population	Design	Results	Observations
Stein et al., 2012 (41)	40 ids with AN-R (F40, $\bar{\text{x}}$ = 15.6 ± 1.7); 23 ids with AN-B\P (F23, $\bar{\text{x}}$ = 16.3 ± 1.3); 33 ids with BN\EDONS-P (F33, $\bar{\text{x}}$ = 16.6 ± 1.3); 20 ids HC (F26, $\bar{\text{x}}$ =16.5 ± 2.0)	LS. BDI; Y-BOCS; YBC-ED; RSPM	In admission all ED groups showed ↑ scores for OC symptoms ED-related and non-ED-related vs the HC group (p<0.001). BN/EDNOS-P group showed ↑ in OC ED-related symptoms compared to AN-R group (p<0.05). BN/EDNOS-P group show ↑ psychometric ED-related and no related OC symptoms (p<0.05). AN-R ↑ OC symptoms ED-related at discharge vs the AN-B/P group(p<0.05). AN-B/P group showed no changes at discharge related to OC symptoms. At admission no differences were found between all the groups for orbitofrontal functioning and alternation learning. In AN-R group, olfactory discrimination is positively correlated with compulsive ED symptoms (Y-BC-ED, p<0.01) and with the Y-BC-ED total score (p =0.05). BN/EDNOS-P group alteration learning is ↑ correlated with ED-related OC symptoms (p<0.01). In the AN-R group, olfactory discrimination is ↑ correlated with ED related OC symptoms (p<0.05).	The present study investigated orbitofrontal functioning in ED which share many features with OC symptoms and consequently with OCD. A better orbitofrontal functioning in ED ids could be associated with ED-related OC symptoms. This findings may represent a core feature of the ED that is independent of malnutrition behaviors, but is associated with ED related obsessionality. Larger sample of subjects may be an interesting suggestion for future researches.
Breithaupt et al., 2014 (42)	9 ids with AN (F9, $\bar{\text{x}}$ = 16.00 ± 1.00)	CS. EDE-Q; BCQ; WASI; ChOCI; CBOCI;	↑ BC behaviors positively correlates with OC behaviors (p <.0.5). Patients with BC behaviors report ↑ OC symptoms. Idiosyncratic checking behaviors were significantly related to cognition inhibition (p < 0.05): Ids with ↓ levels of cognitive inhibition had ↑ of idiosyncratic checking Ids with ↑ cognitive inhibition had ↓ idiosyncratic checking.	A negative relationship between cognitive inhibition and idiosyncratic BC was found out. These findings provide preliminary evidence that idiosyncratic BC in AN patients may suggest a neuropsychological profile similar in those with checking behaviors in OCD. Results are based on 9 ids, a larger sample of subjects may be an interesting suggestion for future researches.
Brooks et al., 2014 (43)	15 ids with EDNOS (F15, $\bar{\text{x}}$ =15.00 ± 1.36); 20 ids HC (F20, $\bar{\text{x}}$ =14.20 ± 1.11)	CS. EDE-Q; OCI-R; BIS-11 N-BACK; fMRI	EDNOS group showed ↑ scores on OCI scale (p<0.001) compared to HC. EDNOS showed ↑ scores on BIS 11 (p= 0.0005) compared to HC. At fMRI the EDNOS group showed ↑ activation in the medial frontal gyrus (p<0.0001). HC group did not show any brain region with ↑ activation than the EDNOS group.	EDONS patients showed marked activation in the PFC circuitry associated with better WM performance and higher OC symptoms. These results were not found in the HC group. These findings show that ED-related thoughts may be related to a neuronal response in the early stages of the disease. Results are based only on the Sweden population.
Lewis et al., 2018 (44)	94 ids with AN-R (F94, $\bar{\text{x}}$ =15.75 ± 1.50);67 ids with AN-B\P (F67, $\bar{\text{x}}$ =16.23 ± 1.33); 48 ids with BN (F48, $\bar{\text{x}}$ =16.70± 0.99)	LS. Y-BOCS; YBC-ED; EAT-26; BDI; STAI;	AN-B/P group patients showed ↑ in Y-BOCS (p=0.006), YBC-EDS (p =0.017), EAT-26 (p=0.003) and BDI (p<0.001) compared to AN-R or BN patients. AN-B/P and BN group patients showed ↑ on Y-BOCS (p=0.006), compared to AN-R patients. No difference was found between the groups AN-R, AN-B/P, BN for YBC-EDS and EAT 26. Results showed ↑ for ED, OC and depressive symptoms in AN-B/P patients (p<0.05) in the acute phase of disease compared with patients with AN-R or BN. AN-B/P patients showed ↑ OC symptoms in the stabilized phase of the disease compared to the patients with restrictive symptoms (p<0.05).	The present study aimed to investigate the relationship between OCD symptoms and different types of ED. It was found that OC symptoms are more marked in the acute phase of AN-B/P patients compared to AN-R and BN patients. Differently, an improvement in obsessionality was found from the acute phase to the stabilized phase in patients with AN-B/P compared to the AN-R group. These results could be influenced by the stages that have been considered of ED.
Tyszkiewicz-Nwafor et al., 2018 (45)	76 is with AN (F 76 $\bar{\text{x}}$ = 15.6 ± 2.3); 30 ids HC (F 30 $\bar{\text{x}}$ = 15.3 ± 1.5)	CS EAT-26; BDI; YBOCS; BS; ELISA kit.	The concentration of adiponectin in the acAN was ↑ than in HC (*P* = 0.05) and ↑ in the recAN (p = 0.01). Resistin concentrations were ↓ in acAN and recAN than in HC (*P =* 0.00). No difference was observed in resistin concentration in the acAN and recAN groups (p = 0.20). In the acAN group, a negative correlation was noted between adiponectin concentration and YBOCS scores (p = 0.049).	Association between adiponectin with OC symptoms and resistin with depressive symptoms indicate their potential contribution to emotion regulation and AN pathogenesis.
Plana et al., 2019 (46)	116 ids with AN (F 113, VEO 34 $\bar{\text{x}}$ =); 74 ids with OCD(F 37, VEO 31 $\bar{\text{x}}$ =); 65 ids VEO ( $\bar{\text{x}}$ = 14,4 ± 2.2); 125 EO ( $\bar{\text{x}}$ = 16,3 ± 1.4)	CS EAT-40; LOI-CV; CAPS; CDI; BS in EDTA; MagNA Pure LC DNA isolation Kit III; LC MagNA Pure system; Illumina	This study confirms a genetic overlap between OCD and AN. Specifically, it describes genetic pleiotropy for age at onset across these disorders, associating two SNPs (rs6311, rs4942587) of the HTR2A with the very-early onset phenotype.	Psychometric evaluations to detect association between comorbidities and genetic risk were not performed. it might be interesting to evaluate this association in combination with the genetic study using a larger sample as well.
Flamarique et al., 2019 (47)	79 ids with AN (F 77 $\bar{\text{x}}$ =16.1 ± 1.2); 32 ids with OCD (F 10 $\bar{\text{x}}$ =15.5 ± 2.6); 74 ids with HC (F 52 $\bar{\text{x}}$ = 16.5 ± 1.4)	CS CAPS; CBT; CDI; CY-BOCS; EAT-40; K-SADS; LOI-CV.	SOP seemed ↑ in AN ids than in OCD ids (p<0.001) or HC ids (p<001). SOP predicted OC, depressive, and ED symptoms in the AN group (p<0.001). SPP total score among HC ids was similar to that in the AN ids and ↑ than that in the OCD ids (p = 0.003). In HC, SPP was related to OC (p = 0.002), depressive (p = 0.011), and ED symptomatology (p = 0.006).	This is the first study comparing different dimensions of perfectionism. It could have clinical implications in general treatment of AN ids and also in order to prevent from developing obsessive, depressive and eating symptoms in both AN and healthy ids. A limitation can be related to the fact that comparison with other studies that have used different scales may make these results difficult to compare and generalize.
Bohon et al., 2020 (48)	14 ids with WR-AN (F14, $\bar{\text{x}}$ = 15.79 ± 1.93); 11 ids with OCD (F11, $\bar{\text{x}}$ = 15.64 ± 2.01); 24 HC (F24, $\bar{\text{x}}$ = 15.29 ± 1.65)	CS. WCTS during fMRI.	OCD ids showed ↑ perseverative errors (set shifting deficits) than WR-AN (p=0.010) and HC (p=0.047). WR-AN and HC had no differences. No differences in neural activity at fMRI during WCTS between groups. OCD ids showed positive correlation between perseverative errors and brain activity in the rFP, rIFG and rMFG (p=0.002). This correlation was not significant examining the whole brain. This correlation was not present in HC. In WR-AN perseverative errors and brain activity were related with no differences compare to both HC and OCD.	Set shifting deficits could be related to OC symptoms in adolescence, more than ED symptoms. rIFG is related to inhibition, in OCD ids a ↑ perseverative errors could be related to a ↑ effort to inhibit responses. A limitation is that authors assessed WR-AN and not AN in an acute phase.
Reilly et al., 2022 (49)	110 ids with BN (F 103, $\bar{\text{x}}$ = 15.80 ± 1.51)	RCT: CBT vs FBT. EDE; CYBOCS; YBC-ED	In BN ids ED related OC symptoms ↓ after an 18 session treatment with CBT or FBT and ↓ at 6- and 12-months follow-up (p<0.001). ED related OC symptoms account for ED-symptoms level at 12-month follow-up (p<0.001). OC general symptoms did not change significantly after CBT or FBT treatment or at follow-up. OC general symptom changes were not related to ED symptoms variations.	BN in adolescence shows a strong relationship between ED symptoms and ED related OC symptoms, but not with general OC symptoms. These results could be influenced by the overlapping in content of EDE and YBC-ED.
Camprodon-Boadas et al., 2023 (50)	20 ids with OCD (F11, $\bar{\text{x}}$ = 14.10 ± 1.86); 20 ids with AN (F20 $\bar{\text{x}}$ = 14.85 ± 1.59); 20 with FEP (F12 ( $\bar{\text{x}}$ = 15.79 ± 1.26)	CS. BABS; BDI-II	AN group showed ↑ severity of depressive symptoms than OCD group (p<0.001). At BABS OCD group had ↓ scores in almost all items than AN and FEP groups (p<0.001). No differences between AN and OCD groups in fixation of ideas (item 4). OCD group had ↓ scores than the AN group in insight (item 6)(p = 0.016). Both OCD group (p = 0.006) and AN group (p < 0.001) had ↓ scores than FEP group in delusions of reference (item 7). AN and FEP appeared to be different only in item 7. OCD group scored ↓ than the FEP (p = 0.001) and AN (p < 0.001) groups in the total delusional belief score.	Results highlight a ↑ presence and severity of delusional thinking in AN ids than OCD ids, that showed low scores in BABS. Intriguing AN seems to be more similar to FEP level of delusional beliefs compared to OCD in adolescent patients. In particular AN group shows ↓ level of insight than OCD, this could cause the development of delusional features in thought, preoccupations and beliefs related to the disorder. Results are based only on the assessment of patients throughout the BABS.

acAN, acute phase of anorexia nervosa; AN, anorexia nervosa; AN-R, anorexia nervosa-restricting type; AN-B/P, anorexia nervosa -binge/purge type; BABS, Brown Assessment of Beliefs Scale; BC, Body Checking; BCQ, Body Checking Questionnaire; BDI-II, Beck Depression Inventory; BIS-11, Barratt impulsiveness scale; BMI, Body Mass Index; BN, bulimia nervosa; BS, blood samples; CAPS, Child and Adolescent Perfectionism Scale; CBOCI, Clark–Beck Obsessive Compulsive Scale; CBT, Cognitive Behavioral Therapy; CDI, Childhood Depression Inventory; ChOCI, Children Obsessive Compulsive Inventory; CS, cross-sectional study; CYBOCS, Children’s Yale-Brown Obsessive Compulsive Scale; EAT-26, Eating Attitude Test; EAT-40, Eating Attitudes Test; ED, eating disorder; EDE-Q, eating disorder examination questionnaire; EDNOS, eating disorder not otherwise specified; EDNOS-P, eating disorder not otherwise specified-purging type; EO, early onset; F, female; FBT, Family-Based Treatment; FEP, first-episode psychosis; fMRI, functional resonance magnetic imaging; HC, healthy controls; ids, individuals; LOI-CV, Leyton Obsessional Inventory - Child Version; LS, longitudinal study; K-SADS, Kiddie Schedule for Affective Disorders and Schizophrenia; N-BACK, n-back task; OC, obsessive compulsive; OCI-R, Obsessive-compulsiveness inventory; OCD, obsessive-compulsive disorder; PC, psychiatric controls; RCT, randomized controlled trial; recAN, anorexia nervosa recovery; rFP, right frontal pole; rIFG, right inferior frontal gyrus; rMFG, right middle frontal gyrus; RSPM, Raven Standard Progressive Matrices; SNPs, single-nucleotide polymorphism; SOP, self-oriented perfectionism; SPP, self-oriented perfectionism; STAI, State-Trait Anxiety Inventory; VEO, very early onset; vs, versus; WASI, Wechsler Abbreviated Scale of Intelligence; WCTS, Wisconsin Card Sorting Test; WM, working memory; WR-AN, weight-restored anorexia nervosa; $\bar{\text{x}}$ , mean age; YBC-ED, Yale-Brown-Cornell Eating Disorders Scale; YBOCS, Yale-Brown Obsessive Compulsive Scale; ↑, higher/more/increased; ↓, lower/lesser/decreased.

### 2.4 Risk of bias assessment

In order to evaluate the reliability of the review, its quality, and to rigorously analyze the outcomes of the chosen studies, a risk of bias analysis was performed. This analysis adhered to the indications and criteria put forth by the Agency for Health Care Research and Quality (51). Each study underwent bias assessment in accordance with the stipulated criteria, encompassing selection bias, performance bias, detection bias, attrition bias, and reporting bias. Subsequently, a bias level, categorized as low, medium, or high, was assigned to each study based on the assessment. The included studies were independently evaluated by the authors, and any disparities in the assessments were resolved through discussions. The evaluation of the risk of bias is detailed in the online supplements, specifically in **Supplementary Table 2**.

## 3 Results

### 3.1 Search results

The aforementioned search yielded an initial collection of 335 articles, published from 1968 to September 2023. Through the application of inclusion and exclusion criteria, a total of 324 articles were excluded, culminating in a final selection of 10 articles (refer to **Table 1**). Detailed explanations for the rejection of each study can be found in the online supplements, specifically **Supplementary Table 1**. The complete search results, along with reasons for exclusion when applicable, are depicted in the PRISMA flowchart, accessible in the online supplements (**Supplementary Figure 1**).

### 3.2 Overview of the included studies

The 10 included studies examined various different aspects. Many focused on OC symptomatology in patients with ED, while others analyzed clinical differences between patients with OCD and ED. None assessed the presence of ED symptomatology in OCD patients. Furthermore, the studies evaluated neuroimaging, genetic, neurobiological, and neurocognitive factors. In this paragraph, we provide summaries of each study included in the review.

Stein and colleagues (41) conducted a longitudinal study in 2012 in a sample of adolescent females. According to DSM-IV, 40 were diagnosed with anorexia nervosa, restricting type (mean age 15.6); 23 with AN, binge/purge (B/P) type (mean age 16.3); 33 with BN or eating disorder not otherwise specified (EDNOS) B/P type (mean age 16.6); and 20 healthy controls (HC) (mean age 16.5). The 4 groups with ED were inpatients. They were assessed within 14 days of admission. As orbitofrontal dysfunction is an important feature of OCD, the aim of the study was to assess orbitofrontal function in ED. Orbitofrontal function was assessed using alternative learning and olfactory threshold and discrimination. The sample were assessed by Beck Depression Inventory (BDI), a scale to measure the severity of depression; Yale-Brown Obsessive Compulsive Scale (YBOCS), which assesses the severity and type of symptoms in patients with OCD; Yale Brown-Cornell Eating Disorders Scale (YBC-ED) to assess core concerns and rituals associated with ED; Raven Standard Progressive Matrices to measure intelligence. The results showed that ED-related obsessionality is associated in ED with better orbitofrontal cortex (OFC) functioning than in non-ED-related obsessionality.

It appears noteworthy to specify that obsessionality does not refer only to obsession but to the broader cognitive disturbance caused by obsessive-compulsive symptoms (52). Furthermore, all of the ED groups have better scores than the HC on olfactory threshold and olfactory discrimination, but worse scores than the HC on alternative learning.

In 2014, Breithaupt et al. (42) investigated the role of body checking in anorexia nervosa as a behavioral link to OCD in a cross-sectional study, with a sample of 9 female in-patients with AN. The age range was 14 to 17 years. They were assessed using the Eating Disorder Examination Questionnaire (EDE-Q), a self-report questionnaire that evaluates the frequency and severity of behaviors associated with ED diagnosis; Body Checking Questionnaire (BCQ), which assesses the frequency of body checking behaviors; Children Obsessive Compulsive Inventory (ChOCI), which assesses the presence and severity of OCD in children and adolescents up to 17 years old; Clark-Beck Obsessive Compulsive Scale (CBOCI), which assesses the frequency and severity of obsessive-compulsive symptoms; Wechsler Abbreviated Scale of Intelligence (WASI), which evaluates intelligence. The results showed a significant correlation between body checking symptoms and OCD behaviors; indeed, patients with body checking behaviors reported OCD symptoms. The idiosyncratic checking behavior was significantly related to cognitive inhibition. Patients with lower levels of cognitive inhibition had higher symptoms of idiosyncratic body checking. Patients with higher cognitive inhibition had lower symptoms of idiosyncratic body checking. The study showed that increased symptoms of body checking were correlated with symptoms of OCD: idiosyncratic body checking may be an indication of a similar neuropsychological profile between patients with AN and those with obsessive-compulsive checking behavior.

Brooks and colleagues (43) in 2014 conducted a cross-sectional study of 15 outpatients with an eating disorder non otherwise specified (EDNOS) and 20 HC. The average age of the sample with EDNOS was 15 years. The main hypothesis of the study was that impulsivity, rumination and food restriction could be associated with neuronal activity in response to eating stimuli in adolescent patients with an eating disorder. The sample was assessed using the EDE-Q; the Obsessive-Compulsiveness Inventory (OCI), which measures obsessive-compulsive symptoms with 6 subscales investigating scavenging, chewing, neutralizing, obsessing, hoarding and ordering; Barratt Impulsiveness Scale (BIS-11) which is a self-report measuring the trait of impulsivity based on sensation seeking and anxiety; the N-Back Task assesses working memory by presenting a sequence of stimuli one by one. They also used functional magnetic resonance imaging (fMRI) to measure neural responses. The EDNOS patients had higher OCD symptom scores on the OCI scale than the HC. The EDNOS patients also had higher impulsivity scores at BIS-11 than the HC. During fMRI, the EDNOS patients showed greater activation of the medial frontal gyrus, unlike the HC, who showed no activation of brain regions.

In one longitudinal study conducted in 2018, Lewis and colleagues (44) explored the relationship between OCD symptoms and different types of ED. The sample included adolescent female inpatients of whom 94 with a diagnosis of restrictive AN (AN-R) (mean age: 15.7); 67 with a type of binge/purge AN (AN-B/P) (mean age:16.7) and 48 with BN (mean age: 16.7). The following scales were used to assess the sample: YBOCS; YBC-ED; Eating Attitude Test (EAT-26) which is used to measure symptoms and concerns related to the eating disorder; BDI; and the State -Trait Anxiety Inventory (STAI) which assesses trait and state anxiety and aids in the differentiation between anxiety and depression diagnoses. The results showed that in more eating, obsessive and depressive symptoms are discovered in the most acute phase of the illness compared to AN-R or BN. It was also noted that patients with a diagnosis of AN-B/P showed an improvement of OCD symptoms from the acute phase to the stabilization of ED compared to the patients with AN-R. Given this, we can state that OC symptomatology is more severe in the worst phase for patients with AN-B/P than for patients with AN-R and BN. This study demonstrates different patterns of OC symptomatology in different types of ED during the course of the disease.

In a longitudinal study conducted in 2018, Tyszkiewicz-Nwafor and colleagues (45) investigated the different adiponectin and resistin serum levels between acute phase of anorexia nervosa (acAN) and anorexia nervosa recovery (recAN) and the relationship between serum concentrations of selected adipokines and other psychiatric behaviors, such as OC symptoms. The sample included 76 female inpatients acAN from Child and Adolescent Psychiatric Department (mean age: 15,6) and 30 female HC recruited among middle school students (mean age: 15,3). AN patients were diagnosed according to DSM-IV criteria. The following scales were used to assess the sample: EAT-26, BDI, YBOCS. The ELISA kit was used to conduct the biochemical analysis to determine the concentration of adiponectin and resistin on a venous blood sample. The same procedure was repeated on discharge 10.8 ± 1.4 weeks later. The results showed an higher concentration of adiponectin in the acAN than in HC, and was higher in the recAN than in the acAN and in HC. There were lower resistin concentrations in acAN and recAN than in HC, without differences between the acAN and recAN groups. In the acAN group, a negative correlation was noted between adiponectin concentration and YBOCS scores. The association between adiponectin with OC symptoms and resistin with depressive symptoms indicate their potential contribution to emotion regulation and AN pathogenesis.

In 2019 Flamarique and colleagues (46) conducted a cross-sectional study on self-oriented perfectionism (SOP) and socially prescribed perfectionism (SPP) in patients with OCD and patients with ED, in order to investigate their different role in OCD and ED and to find out how perfectionism influenced OC, depressive, and eating symptomatology. Perfectionism is a trait marked by setting unreachable personal standards. SOP and SPP are two different dimensions of perfectionism. SOP is delineated as an internal pursuit of perfection and the establishment of high self-expectations that are unattainable. SPP represents an individual’s perception of external high expectations, with acceptance contingent upon meeting these standards. The sample included out-patients recruited from the Department of Child and Adolescent Psychiatry and Psychology at the Hospital Clínic in Barcelona. According to DSM-IV-TR 79 were diagnosed with anorexia nervosa (mean age: 16.1), 32 were diagnosed with OCD (mean age: 15.5) and 74 were HC (mean age: 16.5). The sample was assessed by Child and Adolescent Perfectionism Scale (CAPS), a scale used in pediatric population to measure perfectionism, which includes two subscales that measure SOP and SPP; Children’s Yale-Brown Obsessive Compulsive Scale (CY-BOCS), which assesses the severity and type of symptoms in child and adolescent patients with OCD; Childhood Depression Inventory (CDI) that assesses depressive symptoms in children and adolescents; Eating Attitudes Test (EAT-40) to assess attitudes and symptoms usually associated with ED; Leyton Obsessional Inventory - Child Version (LOI-CV) to asses OC symptomatology and estimates the level of interference with daily activities; Kiddie Schedule for Affective Disorders and Schizophrenia (K-SADS) semi-structured interview that assesses current and past psychopathology in children and adolescents. The results showed that SOP seemed predominant in patients with AN than in patients with OCD or HC. SOP predicted OC, depressive, and ED symptoms in the AN group. SPP total score among HC was similar to the total score among patients with AN and higher than that totalized by the patients with OCD. In HC, SPP was related to OC, depressive, and eating symptomatology.

In Plana et al. (47), a cross-sectional study of 2019, authors investigated the existence of a genetic overlap between very early onset AN and OCD. The study recruited 116 adolescents diagnosed with AN and 74 adolescents diagnosed with OCD from Department of Child and Adolescent Psychiatry and Psychology at the Hospital Cliínic in Barcelona. Diagnoses were made using the department’s clinical interview, which assessed the patients’ current psychopathology and developmental history according to DSM-IV criteria. Twelve patients from the OCD group also had comorbid AN. Patients were divided based on the age of onset of the disorder into “very early onset” (VEO) and “early onset” (EO). For AN patients, VEO AN was defined as onset before age 13, while EO was defined as onset from 13 to 18 years. For OCD patients, VEO OCD was defined as onset before age 10, and EO was defined as onset from 10 to 18 years. Dividing patients by age, the sample resulted composed by 65 patients with VEO (mean age 14,4) and 125 patients with EO (mean age 16,3). Several tests and questionnaires were provided: EAT-40, LOI-CV, CAPS and CDI. All patients underwent blood sampling, using EDTA tubes. These samples were then subjected to genomic DNA extraction employing the MagNA Pure LC DNA Isolation Kit III along with an LC MagNA Pure system (Roche Diagnostics GmbH, Mannheim, Germany). Subsequently, the concentration of the extracted DNA was determined by measuring absorbance, a process carried out using the ND1000 device (NanoDrop, Wilmington, Delaware). A total of 44 single nucleotide polymorphism (SNPs) covering the target loci and surrounding regions were chosen from 5 candidate genes related to the serotonin system. The study confirmed genetic pleiotropy between OCD and AN for age at onset, associating two SNPs (rs6311, rs4942587) of the HTR2A gene (located on chromosome 13q14) with the VEO phenotype.

In 2020 Bohon and colleagues (48) conducted a cross sectional study using a sample of 14 patients with weight-restored AN (WR-AN), mean age 15.79; 11 patients with OCD, mean age 15.64; 24 HC, mean age 15.29. The sample consisted entirely of out-patients and was assessed using the Wisconsin Card Sorting Test (WCST) while they underwent fMRI. The WCST is a tool used to assess patients’ frontal functions, including flexibility in strategy choice, problem solving, and is also used to assess inability to abstract and persevere through 128 cards. The aim of the study was to evaluate set shifting and task switching in adolescents with AN or OCD and the brain activity corresponding to these processes, comparing the three groups (AN, OCD, HC). They measured brain activity by fMRI imaging while performing the WCTS. The results demonstrated that the OCD groups showed more perseverative errors (set shifting deficits) than the WR-AN and the HC groups for whom no difference was found. Also, no difference was also found in neural activity at fMRI during WCTS between the groups. The OCD group showed a positive correlation between perseverative errors and brain activity in the right frontal pole, right inferior frontal gyrus and in the right middle frontal gyrus; this correlation was not found in HC group. In WR-AN group perseverative errors and brain activity were related with no differences compare to both HC and OCD groups. These results suggest that the similarities between OCD and AN are determined by OC features that are also present in the AN group, rather than by a common neurocognitive signature.

Reilly and colleagues (49) conducted a randomized controlled trial study in 2022, using a sample of 110 out-patients with BN, their mean age was 15.8. OCD traits are often noted in adolescents with ED and predict poor response to therapeutic treatment but emerging data on adults suggests that there may be changes in ED-related OCD symptoms after appropriate treatment. Therefore, the aim of this study was to evaluate possible changes in OCD symptoms with Family-Based Treatment (FBT) or Cognitive Behavioral Therapy (CBT) for BN. The sample was submitted to 18 sessions of FBT or CBT and was evaluated with 6- or 12-month follow-ups by EDE-Q; CYBOCS; and YBC-ED. The results showed that, for patients with BN, after 18 sessions of CBT or FBT, the OC symptoms related to ED decreased; they also decreased at 6- and 12-month follow-ups. At the same time, the OC general symptoms of OCD did not change significantly after CBT or FBT treatment. Furthermore, changes in symptoms of OCD were not correlated with changes in the ED symptoms. Therefore, BN in adolescence has a strong correlation with ED-related OC symptoms but not with general OC symptoms. Both FBT and CBT can be helpful in reducing ED-related OC symptoms but not general OC symptoms which showed no changes during treatment.

Camprodon-Boadas and colleagues (50) conducted a cross-sectional study in 2023 with a sample of 60 individuals divided as follows: 20 diagnosed with OCD average age 14.10, 20 patients diagnosed with AN average age 14. 85 years and 20 patients with a diagnosis of first-episode psychosis (FEP) and mean age of 15.79 years. They were all in-patients. The aim of the study was to compare the presence of delusional thinking between the different samples (OCD, AN, FEP) using the Brown Assessment of Beliefs Scale (BABS). The BABS assesses through delusionality of beliefs in several psychiatric disorders with several items exploring concepts such as conception and fixity of ideas, insight, idea of the other. BDI, on the other hand, measures the severity of depressive symptoms. The results showed that the AN group had higher scores than the OCD group for depressive symptoms. The OCD group had lower scores in almost all items of the BABS than the AN and FEP groups; but no differences were found between the AN and OCD group on fixation of ideas (item 4). Differently, the OCD group showed lower scores than the AN group on insight (item 6). In comparison to the FEP group, lower scores were found in the AN and OCD groups for delusions of reference (item 7), the only item in which AN and FEP showed differences. Lastly, the OCD group scored lower than the FEP and AN group in the total score for delusional beliefs and show an higher level of insight.

## 4 Discussion

The results of the studies included in the review show a complex scenario of overlaps and divergences between ED and OCD during developmental age. These findings explore various neurobiological and clinical aspects and should be contextualized in relation to broader literature findings in the adult population. As mentioned in the introduction section, this review did not include studies solely examining comorbidity rates between OCD and ED in developmental age. However, we hereby present the key results found in the literature, which seem to align with those observed in adulthood. Indeed, in community studies conducted on children and adolescents, ED show high comorbidity rates with OCD, ranging from 28% (37) to 20% (39). Among ED, a particularly high comorbidity between BN and OCD was found in a North American study (65%) (37), while greater comorbidity with AN (3.2%) compared to BN (1.2%) was reported in a German study (38). Epidemiological studies on children and adolescents align with those on adults, indicating high comorbidity of OCD not only with ED but also with other disorders such as depressive and anxiety disorders (2, 37–39). However, unlike in adults, there is no co-occurrence of personality disorders and substance use disorders, as these disorders are generally not present in children (37). In fact, according to the DSM-5-TR (3), a diagnosis of personality disorder can be made for patients with a clinical pattern which is stable and of long duration, typically beginning in adolescence or early adulthood. Furthermore while maladaptive personality traits and patterns may have their roots in early development, personality traits observed during early and middle childhood do not consistently indicate later personality disorder (53).

In the following paragraphs, we discuss the results of the review considering evidences available in the scientific literature. For clarity, we have organized the discussion into paragraphs based on the topics addressed, as follows.

### 4.1 Clinical features

The scientific literature on developmental age presents interesting findings, particularly regarding the clinical relationship and overlap between OCD, AN, and BN. It is observed that there is a general presence of OC symptoms, both ED-related and non-ED-related, in the population with ED. These latter include specific (AN and BN) and unspecified disorders. This underscores the pervasiveness of overlap between ED and OCD during developmental age (41, 43, 44).

In the beginning, it appears that AN-B/P exhibits greater OC symptoms compared to both BN and AN-R (44). Patients with AN-B/P may encompass aspects of the broad spectrum of OC symptomatology, ranging from impulsivity to obsessiveness and compulsiveness, displaying mixed characteristics of both BN and AN (44). Impulsivity might play a significant role as a factor contributing to OC symptom presence (54). In this light, it seems that ED with binge-purge aspects show more prominent and evident OC symptoms, as BN also displays more OC symptoms than AN-R (44). These differences are particularly pronounced in developmental age and early adulthood, as demonstrated by similar findings from Speranza and colleagues (55). In contrast, in adulthood, the differences between various ED types seem to diminish in favor of a generalized presence of severe OC symptoms, indicating a progressive increase in OC symptoms with age (31, 56, 57). AN-R, however, presents a profile with less pronounced obsessiveness but perhaps more resistant to treatment and more prone to chronicity, at least during developmental age (44). This may be due to a comorbidity profile with a higher presence of non-ED-related OC symptoms, such as checking behaviors, showing a lower response to treatments, including psychotherapeutic interventions for ED, and requiring specific treatment for general OC symptoms (42, 49, 58, 59).

A distinction seems to exist within OC symptoms between those related to eating behaviors and those unrelated. OC ED-related symptoms appear to be associated with a better response to treatments, especially those specifically addressing eating pathology. Both CBT and FBT, whether in outpatient or intensive inpatient settings, have been shown to reduce both eating symptoms and OC ED-related symptoms in adolescents with binge-purge ED (41, 49). OC ED-related symptomatology also seems to be more closely linked to the proper functioning of the OFC, one of the areas most affected in OCD (41). The higher presence of non-ED-related obsessive symptoms associated with ED might signify a worse prognosis in adulthood (12, 13).

From a clinical perspective, perfectionism can also represent a worsening element in the psychopathological picture in both OCD and AN. Perfectionism is a characteristic associated with various psychiatric disorders, often observed in OCD and AN in adolescent and adult populations (60, 61). Despite this, in the only study directly comparing adolescent patients with OCD and AN, AN shows a higher presence of perfectionism, particularly self-oriented perfectionism (46). This aspect seems to correlate AN more strongly with the development of depressive symptoms, eating disorders, as well as OC symptoms, indicating a greater complexity of the disorder in terms of comorbidities. Other studies have confirmed the presence of a high level of perfectionism in adolescent patients with AN and a higher presence of perfectionism in adult patients with AN compared to those with OCD (62–65).

Another interesting result emerging from the review is the greater clinical similarity between AN and early-onset psychotic disorders compared to OCD. Patients with AN tend to exhibit increased delusional thinking and a lower level of insight compared to those with OCD (50). This not only suggests a possible greater severity for AN compared to OCD but also indicates another substantial difference between EDs and OCD. While various authors suggest the potential membership of ED and OCD in the same psychopathological spectrum due to common OC characteristics (2, 25–28), the presence of an egodystonic nature in OCD obsessions and compulsions compared to ED could be crucial (66). However, regarding egosyntonicity and egodistonicity in ED and OCD Roncero and colleagues (67) pointed out that there is no difference between egosyntonicity and egodystonicity between different types of ED, therefore ED patients are not completely egosyntonic (67). Indeed, was also found that egosyntonicity and egodystonicity are not associated with any comorbidity (67). Furthermore, Purdon and colleagues in 2007 found for OCD that the association between egosyntonicity and disorder severity may not be linear. Furthermore, Purdon developed an instrument, the Ego-Dystonicity Questionnaire (68) which, when applied to patients with OCD, showed no linear correlations between OCD and egodystonicity (68). These findings suggest a non-linear association between egosyntonicity/egodystonicity and ED or OCD. In conclusion, from a clinical standpoint, a common OC dimension among the disorders is apparent, but there is no evidence that ED belong to the OCD dimension.”

### 4.2 Cognitive flexibility and executive functions

Given the symptomatic manifestations of OCD and ED, characterized by rigid and repetitive behaviors in both disorders, a reduction in executive functions that ensure good cognitive flexibility and inhibition of actions and thoughts could be present. In adolescents with OCD, there appears to be a decrease in cognitive flexibility and inhibition abilities, as evidenced by a deficit in set shifting and an increase in perseverative errors. This impairment seems more pronounced in OCD than in ED, particularly in AN (WR-AN) (48). These results for OCD align with findings in adults, where a greater presence of set shifting alterations has been observed (69, 70), while studies on adolescent OCD have shown inconsistent results (71, 72).

In the adult AN population, inconsistent results emerge, but there is a greater impairment in cognitive inhibition compared to adolescents with AN (73, 74). This might suggest a worsening of this characteristic over time in AN, which, however, remains lower compared to OCD. Alternatively, it could be attributed to the increased development of OC symptoms in ED. Indeed, adult AN patients exhibit more OC symptoms than adolescents (75). The presence of alterations in cognitive flexibility and cognitive inhibition may be linked to the presence of OC symptoms rather than the disorders themselves. The association between worsened cognitive inhibition in AN and the presence of OC symptoms is also suggested by the results of Beithraupt and colleagues (42), showing a negative correlation between checking symptoms and cognitive inhibition. In line with this perspective, one of the studies in the review (41) highlights better executive functions in ED patients with ED-related OC symptoms compared to those with no-ED-related OC symptoms, which are more typical of OCD.

### 4.3 Neural circuits and neurobiological aspects

Examining brain functioning in OCD and ED reveals the existence of various brain regions with alterations that partially overlap. In OCD, the cortico-striato-thalamo-cortical (CSTC) circuit appears central, involving the OFC, ACC, and basal ganglia such as the caudate (dorsal caudate) (76–78). The CSTC circuit, responsible for controlling movement execution, habit formation, and reward aspects typical of OCD and ED symptomatology, not only exhibits hyperactivity (79) but also seems to normalize its activity after OCD treatment (80–82). The differences in the functional alterations of these structures between developmental and adult age in OCD are not clear. Evaluating some studies, structural differences seem to exist, especially in the OFC, with an increase in size of OFC and ACC in the infantile form (83) and a decrease in the adult form (84). However, more in-depth longitudinal studies are needed to better define this aspect.

On the other hand, ED predominantly exhibit alterations in mesolimbic and mesocortical reward circuits (18). Some studies emphasize the possible presence of alterations in the striatum, especially the ventral part (85), in the prefrontal cortex, and thus in some parts of the CSTC circuit (86, 87) in AN and BN. In these latter ED, the ACC and striatum seem relevant as areas implicated in behavioral and thought control, similarly to OCD. However, significant areas involved in the reward system, like the ventral striatum, appear less involved in OCD (88). Therefore, OCD and ED seem to share functional alterations in partially overlapping areas of the prefrontal cortex, striatum, and connecting circuits between these two regions. One region implicated in both types of disorders seems to be the ACC, as there is evidence of its involvement in OCD (89–91), AN (92) and BN (93). The ACC appears to be one of the brain regions most associated with executive processes such as cognitive flexibility and inhibition (20, 94), along with the dorsolateral prefrontal cortex (95).

The developmental age is a time during which the capacity for self-regulation develops rapidly (96–98). The maturation of the prefrontal cortex and the development of this capability are believed to be closely linked (98, 99). Consequently, disruptions in the maturation of these circuits may play a role in the common challenges associated with regulating thoughts, urges, and behaviors observed in these disorders. In OCD and ED, a connection seems to emerge between poorer cognitive inhibition, a greater presence of OC symptoms, and alterations in the functioning of the prefrontal cortex in both adolescents (48) and adults (20). The presence of hyperactivity in the prefrontal cortex appears to be an important aspect in OCD patients as well as in those with ED showing OC symptoms (43, 100, 101). Hyperactivity in the prefrontal cortex in the presence of OC symptoms could be an indirect effect of the attempt to control actions and thoughts by brain areas dedicated to inhibition (102). Consistent with the previous paragraph, alterations in cognitive flexibility and the greater difficulty in inhibiting dysfunctional behaviors, requiring hyperactivity of the prefrontal region, are more associated with the common OC symptomatology than the two disorders per se. In this perspective, OCD shows increased ACC activation (103), while AN shows decreased activation during tests assessing inhibition control and error evaluation (104).

In addition to the functional aspects of brain areas, further partial overlaps between OCD and ED emerge from the study of neurotransmitter systems. The serotonergic system appears to be a common network. However, OCD does not seem to show clear and unequivocal alterations in the serotonergic system but rather in the glutamatergic system (105). In contrast, EDs exhibit major alterations in the serotonin and dopamine systems (18). The evidence of this overlap is mainly empirical, as it arises from the common effectiveness of SSRI drugs in both OCD and ED (21–24). Specifically, among ED, those with a greater response to SSRIs are those with a binge/purge profile (BED and BN) (23, 24), supporting a possible greater presence of common elements between binge/purge profile and OCD.

Another indirect way to demonstrate the common dysfunction of the serotonergic network is by evaluating differences in the presence of adipokines in these patients. Adipokines (adiponectin and resistin) have altered production in AN due to the decrease in adipocytes. Studies show a tendency toward an increase in adiponectin and a reduction in resistin in AN patients (106). These changes in the blood appear to influence the regulation of food intake with an effect on the cortico-limbic circuit. The alterations persist even in AN during the compensatory phase, suggesting a role in maintaining some symptoms or susceptibility to anorexia-like symptomatology. The correlation between OC symptoms and adiponectin suggests an additional neurobiological connection between OCD and AN (45). Adipokines seem to play a role in regulating brain neurotransmitters; it has been observed that resistin inhibits serotonin and norepinephrine at the level of the hypothalamus (107), while adiponectin appears to (102)increase brain serotonin levels (108). A possible negative correlation has emerged between OC symptoms and adiponectin concentration in AN patients (45). Furthermore, other studies (109, 110) confirm that adiponectin levels are decreased in OCD. These results, along with those found in the study by Tyszkiewicz-Nwafor, seem to confirm the presence of a correlation between adipokines and serotonin, and thus between adipokines and disorders such as OCD or ED. In AN patients, comorbidity with OC symptoms could be associated with a decreased presence of adiponectin and thus a decrease in serotonin levels with greater dysfunction of the serotonergic system. This appears particularly significant considering that comorbidity with OCD makes AN more severe (12, 13). However, contrasting results seem to emerge regarding adiponectin levels, as it would be decreased in OCD, while it would be increased in AN, despite the two disorders, as mentioned, showing many common neurobiological and symptomatic aspects. This difference could be explained by distinct profiles of alteration in the serotonergic system and symptomatic manifestations.

### 4.4 Genetic implications

A genetic correlation exists between AN and OCD, as evidenced by GWAS; a recent genetic association study identified a genetic overlap (111). However, it is noteworthy that there are no GWAS for BN and BED or other ED (18). Possible common SNPs between ED and OCD have been identified, as emphasized by GWAS studies for AN and OCD. Two SNPs (rs6311, rs4942587) in the HTR2A gene, which encodes for the 5HT2A receptor, appear to be linked to the development of OCD and AN. These SNPs are particularly correlated in patients with childhood onset, suggesting that patients with earlier onset may have greater genetic overlap (47). The HTR2A gene encodes the 5HT2A receptor, and the rs6311 polymorphism is one of the most studied. The rs6311 polymorphism is linked to a promoter variant called -1438 G/A, where the A allele causes an increase in promoter activity and, consequently, receptor activity. Specifically, the presence of the A allele is associated with the development of OCD with earlier onset and more severe symptom manifestation (112, 113). The 5HT2A receptor appears to be one of the most important elements among the pathogenetic alterations of the serotonergic system in OCD. In fact, the efficacy of 5HT2A receptor antagonist agents (such as atypical antipsychotics) as adjunct therapy to SSRIs in OCD patients suggests that blocking this receptor may play a central role in the pathophysiology of OC symptoms (114). In ED, there are less clear results regarding the correlation with alterations of the HTR2A gene and the rs6311 polymorphism. Some studies confirm the association, particularly with the presence of the A allele, especially in restrictive AN, while others deny the association (115). It appears that the G allele may be more prevalent in BN (115). The presence of a common polymorphism in a gene encoding for the 5-HT2A receptor underscores how the alteration of the serotonergic system may be crucial in these two forms of disorders, albeit possibly in different ways. These results confirm the heterogeneous presence of common characteristics; the alteration of the serotonergic system, particularly involving the 5HT2A gene, might be more typical of OCD and be present in both OCD and ED with onset in childhood. This higher genetic overlap could be more typical of childhood onset forms compared to adolescent and adult onset disorders. The study of Plana and colleagues (47), included in this review, focused only on genes related to the serotonergic system. In adults, evaluating a broader range of genes related to different networks, it is evident that OCD and AN show overlapping polymorphisms in genes related to both the serotonergic and glutamatergic systems, as well as neurotrophin genes (111). However, does not appear a specific correlation with the HTR2A gene, despite being examined. Building on the information provided in the previous paragraphs, the increased occurrence of serotonergic system alterations in patients with childhood onset AN should correspond to a higher prevalence of OC symptoms compared to cases with onset in adolescence or adulthood. However, specific studies on clinical features of ED with childhood onset are required to better delineate these differences.

## 5 Limitations and strengths

This study is characterized by several strengths and limitations. Firstly, to the best of our knowledge, this is the first systematic review delving into the relationship between OCD and ED in children and adolescents. This may prompt other authors and clinicians to engage in new considerations and reflections on this topic. Nevertheless, it should be noted that despite the limited sample of studies, they represent different research groups with diverse and non-overlapping populations. Moreover, these studies approach the investigated topic from various perspectives, including clinical, genetic, and neurobiological, also with the use of neuroimaging. However, research in this field is constrained, and the final selection of included studies is relatively small. Some of the studies included have small sample sizes, often below 20 subjects. Literature on this topic, both in developmental and adult populations, predominantly focuses on AN and BN within the broader category of ED. There is a notable lack of studies that analyze the relationship between OCD and BED. Studies in this regard could be promising, given the potential correlation between B/P-type symptoms and OC symptoms. This gap is even more apparent in genetic studies, where the only available GWAS is solely for AN. A consequence of the focus on disorders like AN and BN is that the majority of individuals studied are female. This could potentially influence results since OCD often presents a higher frequency in males during the developmental age. A final limitation to highlight is that existing literature primarily concentrates on the presence of OC symptoms in ED, seldom exploring the reverse scenario of potential ED symptoms in the OCD population. This could be an intriguing field for further researches. Another aspect not thoroughly explored in the included studies, yet potentially very intriguing, is the correlation between ED and different subtypes of OCD. This could be an intriguing area for future research given the heterogeneity of OC manifestations.

## 6 Conclusions

The hypotheses formulated by us have been partially confirmed by the results of the review. As hypothesized, mixed results emerge, indicating both differences and similarities between OCD and ED. However, one aspect of our hypotheses is not confirmed. Indeed, OC symptoms are more frequent in ED with a B/P profile (BN and AN-B/P) than in ED with a restrictive profile (AN-R) in developmental age. This difference seems to disappear in adulthood, possibly due to an increase in OC symptoms in ED over time. Therefore, our incorrect hypothesis may be due to the age of patients evaluated in the studies. On the other hand, in developmental age, there may be a greater severity, a lesser response to treatments, and a higher rate of chronicity of OC symptoms in AN-R patients. This could be as ED with a B/P profile, compared to AN-R, present more ED-related OC symptoms. These show a better response to ED psychotherapeutic treatments and a better neurocognitive profile than non-ED-related OC symptoms. A take home message is that OC symptomatology appears to be a common dimension in both OCD and ED, with a wide range of manifestations, rather than representing just a comorbidity or the inclusion of ED within the OCD spectrum. When presents, OC symptomatology, exhibits transversal characteristic alterations in the ACC and poorer cognitive flexibility. These correlations could be highlighted by genetic overlaps between disorders. These aspects may be particularly evident analyzing the developmental age population. Further studies in this area are needed to gain deeper insights into the characteristics of OCD and ED. A better clinical and neurobiological definition, delineating differences and overlaps, could significantly improve treatment selection and thereby influence the outcome of these patients.

## Data availability statement

The original contributions presented in the study are included in the article/**Supplementary Material**. Further inquiries can be directed to the corresponding author.

## Author contributions

ML: Conceptualization, Investigation, Methodology, Supervision, Writing – original draft, Writing – review & editing. DB: Writing – original draft, Writing – review & editing. AB: Methodology, Writing – original draft, Writing – review & editing. VV: Writing – original draft, Writing – review & editing. CDV: Writing – original draft, Writing – review & editing. SV: Writing – original draft, Writing – review & editing. MP: Writing – original draft, Writing – review & editing. VZ: Writing – review & editing.

## References

[1] Heyman I Fombonne E Simmons H Ford T Meltzer H Goodman R . Prevalence of obsessive-compulsive disorder in the British nationwide survey of child mental health. Int Rev Psychiatry Abingdon Engl. (2003) 15:178–84. doi: 10.1080/0954026021000046146 12745330

[2] Altman SE Shankman SA . What is the association between obsessive-compulsive disorder and eating disorders? Clin Psychol Rev. (2009) 29:638–46. doi: 10.1016/j.cpr.2009.08.001 19744759

[3] American Psychiatric Association . *Diagnostic and statistical manual of mental disorders: DSM-5-TR.* Fifth edition, text revision. Washington, DC: American Psychiatric Association Publishing (2022). p. 1050.

[4] Rasmussen SA Eisen JL . The epidemiology and clinical features of obsessive compulsive disorder. Psychiatr Clin North Am. (1992) 15:743–58. doi: 10.1016/S0193-953X(18)30205-3 1461792

[5] Norberg MM Calamari JE Cohen RJ Riemann BC . Quality of life in obsessive-compulsive disorder: an evaluation of impairment and a preliminary analysis of the ameliorating effects of treatment. Depress Anxiety. (2008) 25:248–59. doi: 10.1002/da.20298 17352377

[6] Hirschtritt ME Bloch MH Mathews CA . Obsessive-compulsive disorder: advances in diagnosis and treatment. JAMA. (2017) 317:1358–67. doi: 10.1001/jama.2017.2200 28384832

[7] Abramowitz JS Taylor S McKay D . Obsessive-compulsive disorder. Lancet Lond Engl. (2009) 374:491–9. doi: 10.1016/S0140-6736(09)60240-3 19665647

[8] Geller DA . Obsessive-compulsive and spectrum disorders in children and adolescents. Psychiatr Clin North Am. (2006) 29:353–70. doi: 10.1016/j.psc.2006.02.012 16650713

[9] Javaras KN Runfola CD Thornton LM Agerbo E Birgegård A Norring C . Sex- and age-specific incidence of healthcare-register-recorded eating disorders in the complete swedish 1979–2001 birth cohort. Int J Eat Disord. (2015) 48:1070–81. doi: 10.1002/eat.22467 PMC5028825 26769444

[10] Treasure J Duarte TA Schmidt U . Eating disorders. Lancet. (2020) 395:899–911. doi: 10.1016/S0140-6736(20)30059-3 32171414

[11] Steinhausen H Jensen CM . Time trends in lifetime incidence rates of first-time diagnosed anorexia nervosa and bulimia nervosa across 16 years in a danish nationwide psychiatric registry study. Int J Eat Disord. (2015) 48:845–50. doi: 10.1002/eat.22402 25809026

[12] Thiel A Broocks A Ohlmeier M Jacoby GE Schüssler G . Obsessive-compulsive disorder among patients with anorexia nervosa and bulimia nervosa. Am J Psychiatry. (1995) 152:72–5. doi: 10.1176/ajp.152.1.72 7802124

[13] Drakes DH Fawcett EJ Rose JP Carter-Major JC Fawcett JM . Comorbid obsessive-compulsive disorder in individuals with eating disorders: An epidemiological meta-analysis. J Psychiatr Res. (2021) 141:176–91. doi: 10.1016/j.jpsychires.2021.06.035 34216946

[14] Klein DN Riso LP . Psychiatric disorders: Problems of boundaries and comorbidity. Basic Issues Psychopathol. (1993), 19–66.

[15] Micali N Hilton K Nakatani E Heyman I Turner C Mataix-Cols D . Is childhood OCD a risk factor for eating disorders later in life? A longitudinal study. Psychol Med. (2011) 41:2507–13. doi: 10.1017/S003329171100078X 21733209

[16] Watson HJ Yilmaz Z Thornton LM Hübel C Coleman JRI Gaspar HA . Genome-wide association study identifies eight risk loci and implicates metabo-psychiatric origins for anorexia nervosa. Nat Genet. (2019) 51:1207–14. doi: 10.1038/s41588-019-0439-2 PMC6779477 31308545

[17] International Obsessive Compulsive Disorder Foundation Genetics Collaborative (IOCDF-GC) OCD Collaborative Genetics Association Studies (OCGAS) . Revealing the complex genetic architecture of obsessive-compulsive disorder using meta-analysis. Mol Psychiatry. (2018) 23:1181–8. doi: 10.1038/mp.2017.154 PMC6660151 28761083

[18] Bulik CM Coleman JRI Hardaway JA Breithaupt L Watson HJ Bryant CD . Genetics and neurobiology of eating disorders. Nat Neurosci. (2022) 25:543–54. doi: 10.1038/s41593-022-01071-z PMC9744360 35524137

[19] Song W Wang W Yu S Lin GN . Dissection of the genetic association between anorexia nervosa and obsessive–compulsive disorder at the network and cellular levels. Genes. (2021) 12:491. doi: 10.3390/genes12040491 33801746 PMC8065602

[20] Marsh R Maia TV Peterson BS . Functional disturbances within frontostriatal circuits across multiple childhood psychopathologies. Am J Psychiatry. (2009) 166:664–74. doi: 10.1176/appi.ajp.2009.08091354 PMC2734479 19448188

[21] Jarry JL Vaccarino FJ . Eating disorder and obsessive-compulsive disorder: neurochemical and phenomenological commonalities. J Psychiatry Neurosci JPN. (1996) 21:36–48. 8580116 PMC1188732

[22] Goodman WK Price LH Rasmussen SA Delgado PL Heninger GR Charney DS . Efficacy of fluvoxamine in obsessive-compulsive disorder. A double-blind comparison with placebo. Arch Gen Psychiatry. (1989) 46:36–44. doi: 10.1001/archpsyc.1989.01810010038006 2491940

[23] Leombruni P Pierò A Lavagnino L Brustolin A Campisi S Fassino S . A randomized, double-blind trial comparing sertraline and fluoxetine 6-month treatment in obese patients with Binge Eating Disorder. Prog Neuropsychopharmacol Biol Psychiatry. (2008) 32:1599–605. doi: 10.1016/j.pnpbp.2008.06.005 18598735

[24] Milano W Siano C Putrella C Capasso A . Treatment of bulimia nervosa with fluvoxamine: a randomized controlled trial. Adv Ther. (2005) 22:278–83. doi: 10.1007/BF02849936 16236688

[25] Rothenberg A . Adolescence and eating disorder: the obsessive-compulsive syndrome. Psychiatr Clin North Am. (1990) 13:469–88. doi: 10.1016/S0193-953X(18)30355-1 2235695

[26] Rothenberg A . Eating disorder as a modern obsessive-compulsive syndrome. Psychiatry. (1986) 49:45–53. doi: 10.1080/00332747.1986.11024306 3458264

[27] Hsu LK Kaye W Weltzin T . Are the eating disorders related to obsessive compulsive disorder? Int J Eat Disord. (1993) 14:305–18. doi: 10.1002/1098-108x(199311)14:3<305::aid-eat2260140309>3.0.co;2-l 8275067

[28] Bulik CM Beidel DC Duchmann E Weltzin TE Kaye WH . Comparative psychopathology of women with bulimia nervosa and obsessive-compulsive disorder. Compr Psychiatry. (1992) 33:262–8. doi: 10.1016/0010-440X(92)90051-Q 1643868

[29] Bartz JA Hollander E . Is obsessive–compulsive disorder an anxiety disorder? Prog Neuropsychopharmacol Biol Psychiatry. (2006) 30:338–52. doi: 10.1016/j.pnpbp.2005.11.003 16455175

[30] Treasure J . Where do eating disorders lie on the diagnostic spectrum and what does it mean? Nord J Psychiatry. (2006) 60:27–31. doi: 10.1080/08039480500517984 16500796

[31] Halmi KA Sunday SR Klump KL Strober M Leckman JF Fichter M . Obsessions and compulsions in anorexia nervosa subtypes. Int J Eat Disord. (2003) 33:308–19. doi: 10.1002/eat.10138 12655628

[32] Matsunaga H Miyata A Iwasaki Y Matsui T Fujimoto K Kiriike N . A comparison of clinical features among Japanese eating-disordered women with obsessive-compulsive disorder. Compr Psychiatry. (1999) 40:337–42. doi: 10.1016/S0010-440X(99)90137-2 10509614

[33] Hasler G LaSalle-Ricci VH Ronquillo JG Crawley SA Cochran LW Kazuba D . Obsessive–compulsive disorder symptom dimensions show specific relationships to psychiatric comorbidity. Psychiatry Res. (2005) 135:121–32. doi: 10.1016/j.psychres.2005.03.003 15893825

[34] Summerfeldt LJ Hood K Antony MM Richter MA Swinson RP . Impulsivity in obsessive-compulsive disorder: comparisons with other anxiety disorders and within tic-related subgroups. Pers Individ Differ. (2004) 36:539–53. doi: 10.1016/S0191-8869(03)00113-2

[35] Wade TD Tiggemann M Bulik CM Fairburn CG Wray NR Martin NG . Shared temperament risk factors for anorexia nervosa: A twin study. Psychosom Med. (2008) 70:239–44. doi: 10.1097/PSY.0b013e31815c40f1 18158375

[36] Wonderlich SA Connolly KM Stice E . Impulsivity as a risk factor for eating disorder behavior: Assessment implications with adolescents. Int J Eat Disord. (2004) 36:172–82. doi: 10.1002/eat.20033 15282687

[37] Convertino AD Blashill AJ . Psychiatric comorbidity of eating disorders in children between the ages of 9 and 10. J Child Psychol Psychiatry. (2022) 63:519–26. doi: 10.1111/jcpp.13484 34225382

[38] Jaite C Hoffmann F Glaeske G Bachmann CJ . Prevalence, comorbidities and outpatient treatment of anorexia and bulimia nervosa in German children and adolescents. Eat Weight Disord - Stud Anorex Bulim Obes. (2013) 18:157–65. doi: 10.1007/s40519-013-0020-4 23760844

[39] Mohammadi MR Mostafavi S Hooshyari Z Khaleghi A Ahmadi N Molavi P . Prevalence, correlates and comorbidities of feeding and eating disorders in a nationally representative sample of Iranian children and adolescents. Int J Eat Disord. (2020) 53:349–61. doi: 10.1002/eat.23197 31742760

[40] Page MJ McKenzie JE Bossuyt PM Boutron I Hoffmann TC Mulrow CD . The PRISMA 2020 statement: an updated guideline for reporting systematic reviews. BMJ. (2021) 372:n71. doi: 10.1136/bmj.n71 33782057 PMC8005924

[41] Stein D Gross-Isseroff R Besserglick R Ziv A Mayer G Yaroslavsky A . Olfactory function and alternation learning in eating disorders. Eur Neuropsychopharmacol. (2012) 22:615–24. doi: 10.1016/j.euroneuro.2011.12.006 22858418

[42] Breithaupt LE Payne HA Rose M . Body checking as a behavioral link: A preliminary study assessing inhibition and its association to idiosyncratic body checking in anorexia nervosa. Eat Behav. (2014) 15:591–4. doi: 10.1016/j.eatbeh.2014.08.003 25218356

[43] Brooks SJ Solstrand Dahlberg L Swenne I Aronsson M Zarei S Lundberg L . Obsessive-compulsivity and working memory are associated with differential prefrontal cortex and insula activation in adolescents with a recent diagnosis of an eating disorder. Psychiatry Res Neuroimaging. (2014) 224:246–53. doi: 10.1016/j.pscychresns.2014.10.001 25456522

[44] Lewis YD Gilon Mann T Enoch-Levy A Dubnov-Raz G Gothelf D Weizman A . Obsessive–compulsive symptomatology in female adolescent inpatients with restrictive compared with binge–purge eating disorders. Eur Eat Disord Rev. (2019) 27:224–35. doi: 10.1002/erv.2638 30198142

[45] Tyszkiewicz-Nwafor M Slopien A Dmitrzak-Węglarz M Rybakowski F . Adiponectin and resistin in acutely ill and weight-recovered adolescent anorexia nervosa: Association with psychiatric symptoms. World J Biol Psychiatry. (2019) 20:723–31. doi: 10.1080/15622975.2018.1492735 30264643

[46] Flamarique I Plana MT Castro-Fornieles J Borràs R Moreno E Lázaro L . Comparison of perfectionism dimensions in adolescents with anorexia nervosa or obsessive-compulsive disorder. J Can Acad Child Adolesc Psychiatry J Acad Can Psychiatr Enfant Adolesc. (2019) 28:45–54. PMC6691799 31447902

[47] Plana MT Torres T Rodríguez N Boloc D Gassó P Moreno E . Genetic variability in the serotoninergic system and age of onset in anorexia nervosa and obsessive-compulsive disorder. Psychiatry Res. (2019) 271:554–8. doi: 10.1016/j.psychres.2018.12.019 30554102

[48] Bohon C Weinbach N Lock J . Performance and brain activity during the Wisconsin Card Sorting Test in adolescents with obsessive–compulsive disorder and adolescents with weight-restored anorexia nervosa. Eur Child Adolesc Psychiatry. (2020) 29:217–26. doi: 10.1007/s00787-019-01350-4 PMC6868308 31114967

[49] Reilly EE Gorrell S Brosof L Lock J Le Grange D . Characterizing changes in obsessive–compulsive symptoms over the course of treatment for adolescent bulimia nervosa. Int J Eat Disord. (2022) 55:1342–51. doi: 10.1002/eat.23782 PMC9869712 35861249

[50] Camprodon-Boadas P de la Serna E Plana MT Flamarique I Lázaro L Borràs R . Delusional beliefs in adolescents with anorexia nervosa, obsessive-compulsive disorder, or first-episode psychosis: A comparative study. Psychiatry Res. (2023) 328:115490. doi: 10.1016/j.psychres.2023.115490 37748237

[51] Agency for Healthcare Research and Quality . Methods guide for effectiveness and comparative effectiveness reviews. AHRQ Publication (2008) 10:5–348. 21433403

[52] Denys D . Obsessionality & compulsivity: a phenomenology of obsessive-compulsive disorder. Philos Ethics Humanit Med PEHM. (2011) 6:3. doi: 10.1186/1747-5341-6-3 21284843 PMC3041996

[53] Fortunato A Tanzilli A Lingiardi V Speranza A . Personality disorders in childhood: validity of the coolidge personality and neuropsychological inventory for children (CPNI). Int J Environ Res Public Health. (2022) 19:4050. doi: 10.3390/ijerph19074050 35409734 PMC8998288

[54] Berlin GS Hollander E . Compulsivity, impulsivity, and the DSM-5 process. CNS Spectr. (2014) 19:62–8. doi: 10.1017/S1092852913000722 24229702

[55] Speranza M Corcos M Godart N Loas G Guilbaud O Jeammet P . Obsessive compulsive disorders in eating disorders. Eat Behav. (2001) 2:193–207. doi: 10.1016/S1471-0153(01)00035-6 15001030

[56] Matsunaga H Kiriike N Miyata A Iwasaki Y Matsui T Fujimoto K . Prevalence and symptomatology of comorbid obsessive–compulsive disorder among bulimic patients. Psychiatry Clin Neurosci. (1999) 53:661–6. doi: 10.1046/j.1440-1819.1999.00622.x 10687747

[57] Amianto F Secci I Arletti L Davico C Abbate Daga G Vitiello B . Obsessive–compulsive symptoms in young women affected with anorexia nervosa, and their relationship with personality, psychopathology, and attachment style. Eat Weight Disord - Stud Anorex Bulim Obes. (2022) 27:1193–207. doi: 10.1007/s40519-021-01252-y PMC8964650 34189704

[58] Fairburn CG . Cognitive behavior therapy and eating disorders. New York: Guilford Press (2008). p. 324.

[59] Strober M Freeman R Morrell W . The long-term course of severe anorexia nervosa in adolescents: survival analysis of recovery, relapse, and outcome predictors over 10–15 years in a prospective study. Int J Eat Disord. (1997) 22:339–60. doi: 10.1002/(sici)1098-108x(199712)22:4<339::aid-eat1>3.0.co;2-n 9356884

[60] Limburg K Watson HJ Hagger MS Egan SJ . The relationship between perfectionism and psychopathology: A meta-analysis. J Clin Psychol. (2017) 73:1301–26. doi: 10.1002/jclp.22435 28026869

[61] Egan SJ Wade TD Shafran R . Perfectionism as a transdiagnostic process: A clinical review. Clin Psychol Rev. (2011) 31:203–12. doi: 10.1016/j.cpr.2010.04.009 20488598

[62] Castro J Gila A Gual P Lahortiga F Saura B Toro J . Perfectionism dimensions in children and adolescents with anorexia nervosa. J Adolesc Health. (2004) 35:392–8. doi: 10.1016/S1054-139X(04)00052-7 15488433

[63] Castro-Fornieles J Gual P Lahortiga F Gila A Casulà V Fuhrmann C . Self-oriented perfectionism in eating disorders. Int J Eat Disord. (2007) 40:562–8. doi: 10.1002/eat.20393 17510925

[64] Cockell SJ Hewitt PL Seal B Sherry S Goldner EM Flett GL . Trait and self presentational dimensions of perfectionism among women with anorexia nervosa. Cognit Ther Res. (2002) 26:745–58. doi: 10.1023/A:1021237416366

[65] Sassaroli S Romero Lauro LJ Maria Ruggiero G Mauri MC Vinai P Frost R . Perfectionism in depression, obsessive-compulsive disorder and eating disorders. Behav Res Ther. (2008) 46:757–65. doi: 10.1016/j.brat.2008.02.007 18394588

[66] Holden NL . Is anorexia nervosa an obsessive-compulsive disorder? Br J Psychiatry. (1990) 157:1–5. doi: 10.1192/bjp.157.1.1 2204458

[67] Roncero M Belloch A Perpiñá C Treasure J . Ego-syntonicity and ego-dystonicity of eating-related intrusive thoughts in patients with eating disorders. Psychiatry Res. (2013) 208:67–73. doi: 10.1016/j.psychres.2013.01.006 23541243

[68] Purdon C Cripps E Faull M Joseph S Rowa K . Development of a measure of egodystonicity. J Cognit Psychother. (2007) 21:198–216. doi: 10.1891/088983907781494537

[69] Shin NY Lee TY Kim E Kwon JS . Cognitive functioning in obsessive-compulsive disorder: a meta-analysis. Psychol Med. (2014) 44:1121–30. doi: 10.1017/S0033291713001803 23866289

[70] Snyder HR Kaiser RH Warren SL Heller W . Obsessive-compulsive disorder is associated with broad impairments in executive function: A meta-analysis. Clin Psychol Sci. (2015) 3:301–30. doi: 10.1177/2167702614534210 PMC4351670 25755918

[71] Andrés S Boget T Lázaro L Penadés R Morer A Salamero M . Neuropsychological performance in children and adolescents with obsessive-compulsive disorder and influence of clinical variables. Biol Psychiatry. (2007) 61:946–51. doi: 10.1016/j.biopsych.2006.07.027 17157271

[72] Beers SR Rosenberg DR Dick EL Williams T O’Hearn KM Birmaher B . Neuropsychological study of frontal lobe function in psychotropic-naive children with obsessive-compulsive disorder. Am J Psychiatry. (1999) 156:777–9. doi: 10.1176/ajp.156.5.777 10327915

[73] Smith KE Mason TB Johnson JS Lavender JM Wonderlich SA . A systematic review of reviews of neurocognitive functioning in eating disorders: The state-of-the-literature and future directions. Int J Eat Disord. (2018) 51:798–821. doi: 10.1002/eat.22929 30102781 PMC6594106

[74] Lang K Stahl D Espie J Treasure J Tchanturia K . Set shifting in children and adolescents with anorexia nervosa: an exploratory systematic review and meta-analysis. Int J Eat Disord. (2014) 47:394–9. doi: 10.1002/eat.22235 24347025

[75] Steinhausen H-C . The outcome of anorexia nervosa in the 20th century. Am J Psychiatry. (2002) 159:1284–93. doi: 10.1176/appi.ajp.159.8.1284 12153817

[76] Baxter LR Schwartz JM Mazziotta JC Phelps ME Pahl JJ Guze BH . Cerebral glucose metabolic rates in nondepressed patients with obsessive-compulsive disorder. Am J Psychiatry. (1988) 145:1560–3. doi: 10.1176/ajp.145.12.1560 3264118

[77] Breiter HC Rauch SL Kwong KK Baker JR Weisskoff RM Kennedy DN . Functional magnetic resonance imaging of symptom provocation in obsessive-compulsive disorder. Arch Gen Psychiatry. (1996) 53:595–606. doi: 10.1001/archpsyc.1996.01830070041008 8660126

[78] Stern ER Taylor SF . Cognitive neuroscience of obsessive-compulsive disorder. Psychiatr Clin North Am. (2014) 37:337–52. doi: 10.1016/j.psc.2014.05.004 25150566

[79] Ting JT Feng G . Neurobiology of obsessive-compulsive disorder: insights into neural circuitry dysfunction through mouse genetics. Curr Opin Neurobiol. (2011) 21:842–8. doi: 10.1016/j.conb.2011.04.010 PMC3192923 21605970

[80] Benkelfat C Nordahl TE Semple WE King AC Murphy DL Cohen RM . Local cerebral glucose metabolic rates in obsessive-compulsive disorder. Patients treated clomipramine. Arch Gen Psychiatry. (1990) 47:840–8. doi: 10.1001/archpsyc.1990.01810210048007 2393342

[81] Park HR Kim IH Kang H McCairn KW Lee DS Kim B-N . Electrophysiological and imaging evidence of sustained inhibition in limbic and frontal networks following deep brain stimulation for treatment refractory obsessive compulsive disorder. PloS One. (2019) 14:e0219578. doi: 10.1371/journal.pone.0219578 31323037 PMC6641158

[82] Le Jeune F Vérin M N’Diaye K Drapier D Leray E Du Montcel ST . Decrease of prefrontal metabolism after subthalamic stimulation in obsessive-compulsive disorder: a positron emission tomography study. Biol Psychiatry. (2010) 68:1016–22. doi: 10.1016/j.biopsych.2010.06.033 20951978

[83] Szeszko PR Christian C MacMaster F Lencz T Mirza Y Taormina SP . Gray matter structural alterations in psychotropic drug-naive pediatric obsessive-compulsive disorder: an optimized voxel-based morphometry study. Am J Psychiatry. (2008) 165:1299–307. doi: 10.1176/appi.ajp.2008.08010033 18413702

[84] Pujol J Soriano-Mas C Alonso P Cardoner N Menchón JM Deus J . Mapping structural brain alterations in obsessive-compulsive disorder. Arch Gen Psychiatry. (2004) 61:720. doi: 10.1001/archpsyc.61.7.720 15237084

[85] Davis C Woodside DB . Sensitivity to the rewarding effects of food and exercise in the eating disorders. Compr Psychiatry. (2002) 43:189–94. doi: 10.1053/comp.2002.32356 11994836

[86] Finch JE Palumbo IM Tobin KE Latzman RD . Structural brain correlates of eating pathology symptom dimensions: A systematic review. Psychiatry Res Neuroimaging. (2021) 317:111379. doi: 10.1016/j.pscychresns.2021.111379 34487978

[87] Geisler D King JA Bahnsen K Bernardoni F Doose A Müller DK . Altered white matter connectivity in young acutely underweight patients with anorexia nervosa. J Am Acad Child Adolesc Psychiatry. (2022) 61:331–40. doi: 10.1016/j.jaac.2021.04.019 33989747

[88] Berridge KC Kringelbach ML . Affective neuroscience of pleasure: reward in humans and animals. Psychopharmacol (Berl). (2008) 199:457–80. doi: 10.1007/s00213-008-1099-6 PMC3004012 18311558

[89] Maltby N Tolin DF Worhunsky P O’Keefe TM Kiehl KA . Dysfunctional action monitoring hyperactivates frontal–striatal circuits in obsessive–compulsive disorder: an event-related fMRI study. NeuroImage. (2005) 24:495–503. doi: 10.1016/j.neuroimage.2004.08.041 15627591

[90] Ursu S Stenger VA Shear MK Jones MR Carter CS . Overactive action monitoring in obsessive-compulsive disorder: evidence from functional magnetic resonance imaging. Psychol Sci. (2003) 14:347–53. doi: 10.1111/1467-9280.24411 12807408

[91] Gehring WJ Himle J Nisenson LG . Action-monitoring dysfunction in obsessive-compulsive disorder. Psychol Sci. (2000) 11:1–6. doi: 10.1111/1467-9280.00206 11228836

[92] Kaye W . Neurobiology of anorexia and bulimia nervosa. Physiol Behav. (2008) 94:121–35. doi: 10.1016/j.physbeh.2007.11.037 PMC2601682 18164737

[93] Marsh R Steinglass JE Gerber AJ Graziano O’Leary K Wang Z Murphy D . Deficient activity in the neural systems that mediate self-regulatory control in bulimia nervosa. Arch Gen Psychiatry. (2009) 66:51. doi: 10.1001/archgenpsychiatry.2008.504 19124688 PMC2759684

[94] Marsh R Zhu H Schultz RT Quackenbush G Royal J Skudlarski P . A developmental fMRI study of self-regulatory control. Hum Brain Mapp. (2006) 27:848–63. doi: 10.1002/hbm.20225 PMC2292452 16421886

[95] Oldrati V Patricelli J Colombo B Antonietti A . The role of dorsolateral prefrontal cortex in inhibition mechanism: A study on cognitive reflection test and similar tasks through neuromodulation. Neuropsychologia. (2016) 91:499–508. doi: 10.1016/j.neuropsychologia.2016.09.010 27647553

[96] Diamond A . Abilities and neural mechanisms underlying AB performance. Child Dev. (1988) 59:523–7. doi: 10.1111/j.1467-8624.1988.tb01486.x 3359870

[97] Luna B Garver KE Urban TA Lazar NA Sweeney JA . Maturation of cognitive processes from late childhood to adulthood. Child Dev. (2004) 75:1357–72. doi: 10.1111/j.1467-8624.2004.00745.x 15369519

[98] Best JR Miller PH . A developmental perspective on executive function. Child Dev. (2010) 81:1641–60. doi: 10.1111/j.1467-8624.2010.01499.x PMC3058827 21077853

[99] Rubia K Smith AB Woolley J Nosarti C Heyman I Taylor E . Progressive increase of frontostriatal brain activation from childhood to adulthood during event-related tasks of cognitive control. Hum Brain Mapp. (2006) 27:973–93. doi: 10.1002/hbm.20237 PMC6871373 16683265

[100] Evans DW Lewis MD Iobst E . The role of the orbitofrontal cortex in normally developing compulsive-like behaviors and obsessive–compulsive disorder. Brain Cognit. (2004) 55:220–34. doi: 10.1016/S0278-2626(03)00274-4 15134855

[101] Maia TV Cooney RE Peterson BS . The neural bases of obsessive–compulsive disorder in children and adults. Dev Psychopathol. (2008) 20:1251–83. doi: 10.1017/S0954579408000606 PMC3079445 18838041

[102] Roth RM Saykin AJ Flashman LA Pixley HS West JD Mamourian AC . Event-related functional magnetic resonance imaging of response inhibition in obsessive-compulsive disorder. Biol Psychiatry. (2007) 62:901–9. doi: 10.1016/j.biopsych.2006.12.007 17511967

[103] Fitzgerald KD Welsh RC Gehring WJ Abelson JL Himle JA Liberzon I . Error-related hyperactivity of the anterior cingulate cortex in obsessive-compulsive disorder. Biol Psychiatry. (2005) 57:287–94. doi: 10.1016/j.biopsych.2004.10.038 15691530

[104] Pieters GLM De Bruijn ERA Maas Y Hulstijn W Vandereycken W Peuskens J . Action monitoring and perfectionism in anorexia nervosa. Brain Cognit. (2007) 63:42–50. doi: 10.1016/j.bandc.2006.07.009 16962223

[105] Goodman WK Storch EA Sheth SA . Harmonizing the neurobiology and treatment of obsessive-compulsive disorder. Am J Psychiatry. (2021) 178:17–29. doi: 10.1176/appi.ajp.2020.20111601 33384007 PMC8091795

[106] Karageorgiou V Furukawa TA Tsigkaropoulou E Karavia A Gournellis R Soureti A . Adipokines in anorexia nervosa: A systematic review and meta-analysis. Psychoneuroendocrinology. (2020) 112:104485. doi: 10.1016/j.psyneuen.2019.104485 31805456

[107] Brunetti L Orlando G Recinella L Michelotto B Ferrante C Vacca M . Resistin, but not adiponectin, inhibits dopamine and norepinephrine release in the hypothalamus. Eur J Pharmacol. (2004) 493:41–4. doi: 10.1016/j.ejphar.2004.04.020 15189762

[108] Mössner R . Enhancement of serotonin transporter function by tumor necrosis factor alpha but not by interleukin-6. Neurochem Int. (1998) 33:251–4. doi: 10.1016/S0197-0186(98)00026-6 9759920

[109] Atmaca M Ustundag B Metin K Topuz M . Low plasma adiponectin levels in obsessive-compulsive disorder. J Affect Disord. (2009) 117:205–7. doi: 10.1016/j.jad.2008.12.019 19176249

[110] Ari M Ozturk OH Bez Y Arica S Can Y Erduran D . Serum adiponectin and resistin levels in patients with obsessive compulsive disorder. J Affect Disord. (2012) 136:979–82. doi: 10.1016/j.jad.2011.07.029 22119090

[111] Mas S Plana MT Castro-Fornieles J Gassó P Lafuente A Moreno E . Common genetic background in anorexia nervosa and obsessive compulsive disorder: preliminary results from an association study. J Psychiatr Res. (2013) 47:747–54. doi: 10.1016/j.jpsychires.2012.12.015 23337130

[112] Walitza S Wewetzer C Warnke A Gerlach M Geller F Gerber G . 5-HT2A promoter polymorphism –1438G/A in children and adolescents with obsessive-compulsive disorders. Mol Psychiatry. (2002) 7:1054–7. doi: 10.1038/sj.mp.4001105 12476319

[113] Walitza S Bové DS Romanos M Renner T Held L Simons M . Pilot study on HTR2A promoter polymorphism, –1438G/A (rs6311) and a nearby copy number variation showed association with onset and severity in early onset obsessive–compulsive disorder. J Neural Transm. (2012) 119:507–15. doi: 10.1007/s00702-011-0699-1 21874579

[114] Goddard AW Shekhar A Whiteman AF McDougle CJ . Serotoninergic mechanisms in the treatment of obsessive–compulsive disorder. Drug Discov Today. (2008) 13:325–32. doi: 10.1016/j.drudis.2007.12.009 18405845

[115] Monteleone P Maj M . Genetic susceptibility to eating disorders: associated polymorphisms and pharmacogenetic suggestions. Pharmacogenomics. (2008) 9:1487–520. doi: 10.2217/14622416.9.10.1487 18855537

