# Supplementary material for: Similarities and differences between eating disorders and obsessive-compulsive disorder in childhood and adolescence: a systematic review
Source: Front Psychiatry. 2024 Jun 4;15:1407872. doi: 10.3389/fpsyt.2024.1407872 (PMC11183500; doi:10.3389/fpsyt.2024.1407872)
Supplement: Supplementary file 1 [file DataSheet_1.pdf]

Supplementary Materials

Supplementary Figure 1. PRISMA flowchart.

Search strategy:
PubMed: ("eating disorder" OR "anorexia nervosa" OR "binge eating disorder" OR "pica" OR "avoidant restrictive food intake disorder" OR "rumination disorder") AND "obsessive-compulsive disorder" AND ("childhood" OR "child" OR "adolescence" OR "adolescent")
CENTRAL: ("eating disorder" OR "anorexia nervosa" OR "binge eating disorder" OR "pica" OR "avoidant restrictive food intake disorder" OR "rumination disorder") AND "obsessive-compulsive disorder" AND ("childhood" OR "child" OR "adolescence" OR "adolescent")
on 30-11-2023

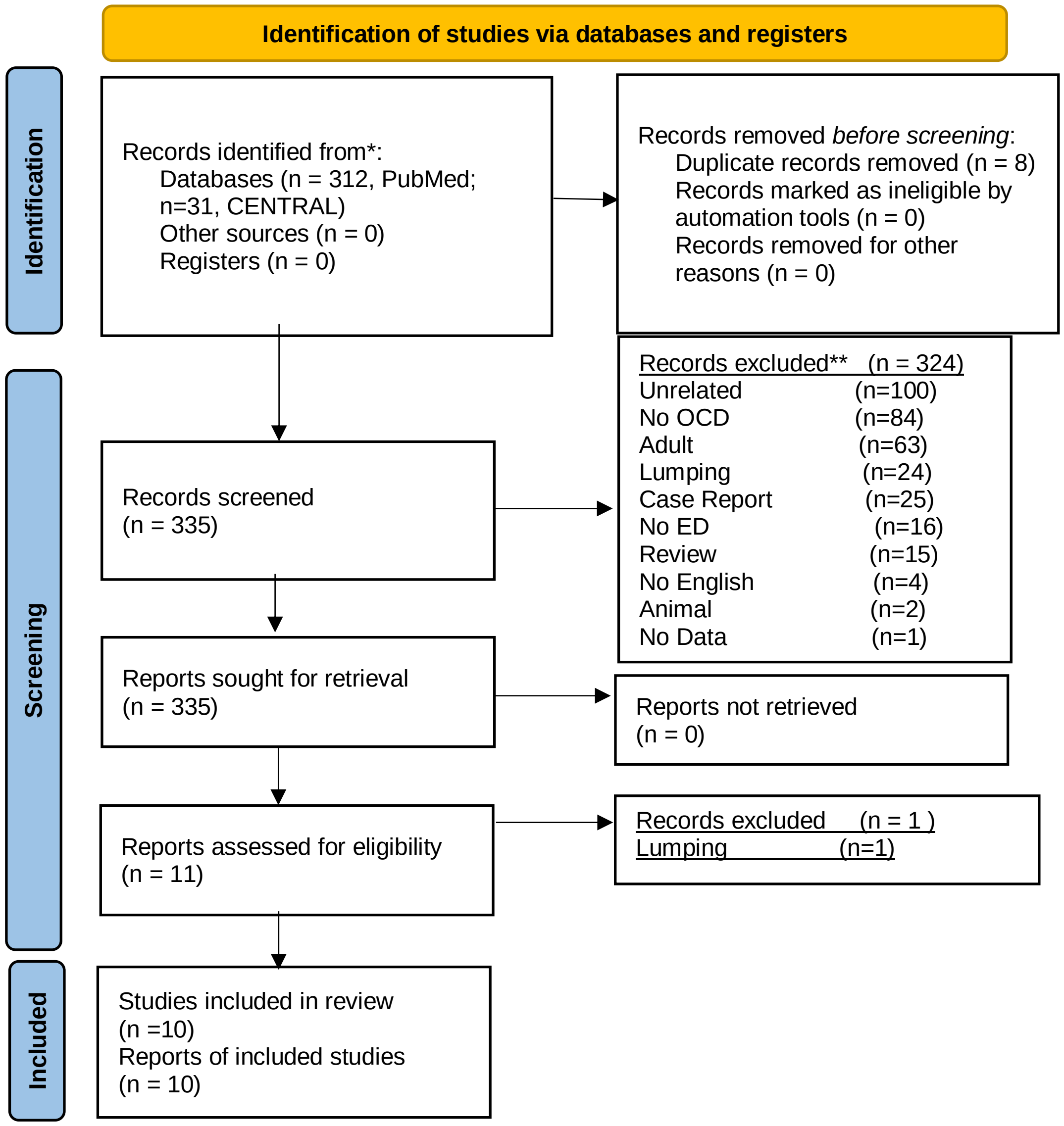

*Consider, if feasible to do so, reporting the number of records identified from each database or register searched (rather than the total number across all databases/registers).

**If automation tools were used, indicate how many records were excluded by a human and how many were excluded by automation tools. *No automation tools were used*

From: Page MJ, McKenzie JE, Bossuyt PM, Boutron I, Hoffmann TC, Mulrow CD, et al. The PRISMA 2020 statement: an updated guideline for reporting systematic reviews. BMJ 2021;372:n71. doi: 10.1136/bmj.n71

Supplementary Figure 2. PRISMA Checklist.

PRISMA 2020 Checklist

Section and Topic	Item #	Checklist item	Location where item is reported
**TITLE**			
Title	1	Identify the report as a systematic review.	Title
**ABSTRACT**			
Abstract	2	See the PRISMA 2020 for Abstracts checklist.	Abstract
**INTRODUCTION**			
Rationale	3	Describe the rationale for the review in the context of existing knowledge.	Introduction
Objectives	4	Provide an explicit statement of the objective(s) or question(s) the review addresses.	Introduction
**METHODS**			
Eligibility criteria	5	Specify the inclusion and exclusion criteria for the review and how studies were grouped for the syntheses.	Methods
Information sources	6	Specify all databases, registers, websites, organisations, reference lists and other sources searched or consulted to identify studies. Specify the date when each source was last searched or consulted.	Methods
Search strategy	7	Present the full search strategies for all databases, registers and websites, including any filters and limits used.	Methods
Selection process	8	Specify the methods used to decide whether a study met the inclusion criteria of the review, including how many reviewers screened each record and each report retrieved, whether they worked independently, and if applicable, details of automation tools used in the process.	Methods
Data collection process	9	Specify the methods used to collect data from reports, including how many reviewers collected data from each report, whether they worked independently, any processes for obtaining or confirming data from study investigators, and if applicable, details of automation tools used in the process.	Methods<br>Acknowledgements
Data items	10a	List and define all outcomes for which data were sought. Specify whether all results that were compatible with each outcome domain in each study were sought (e.g. for all measures, time points, analyses), and if not, the methods used to decide which results to collect.	Methods<br>Supplements
	10b	List and define all other variables for which data were sought (e.g. participant and intervention characteristics, funding sources). Describe any assumptions made about any missing or unclear information.	Supplements
Study risk of bias assessment	11	Specify the methods used to assess risk of bias in the included studies, including details of the tool(s) used, how many reviewers assessed each study and whether they worked independently, and if applicable, details of automation tools used in the process.	Methods<br>Supplements
Effect measures	12	Specify for each outcome the effect measure(s) (e.g. risk ratio, mean difference) used in the synthesis or presentation of results.	N/A
Synthesis methods	13a	Describe the processes used to decide which studies were eligible for each synthesis (e.g. tabulating the study intervention characteristics and comparing against the planned groups for each synthesis (item #5)).	Methods
	13b	Describe any methods required to prepare the data for presentation or synthesis, such as handling of missing summary statistics, or data conversions.	Methods
	13c	Describe any methods used to tabulate or visually display results of individual studies and syntheses.	Methods
	13d	Describe any methods used to synthesize results and provide a rationale for the choice(s). If meta-analysis was performed, describe the model(s), method(s) to identify the presence and extent of statistical heterogeneity, and software package(s) used.	Methods
	13e	Describe any methods used to explore possible causes of heterogeneity among study results (e.g. subgroup analysis, meta-regression).	N/A
	13f	Describe any sensitivity analyses conducted to assess robustness of the synthesized results.	N/A
Reporting bias assessment	14	Describe any methods used to assess risk of bias due to missing results in a synthesis (arising from reporting biases).	Methods<br>Supplements
Certainty assessment	15	Describe any methods used to assess certainty (or confidence) in the body of evidence for an outcome.	Methods
**RESULTS**			
Study selection	16a	Describe the results of the search and selection process, from the number of records identified in the search to the number of studies included in the review, ideally using a flow diagram.	Results<br>Supplements
	16b	Cite studies that might appear to meet the inclusion criteria, but which were excluded, and explain why they were excluded.	Supplements

Study characteristics	17	Cite each included study and present its characteristics.	Results
Risk of bias in studies	18	Present assessments of risk of bias for each included study.	Supplements<br>Limitations and strengths
Results of individual studies	19	For all outcomes, present, for each study: (a) summary statistics for each group (where appropriate) and (b) an effect estimate and its precision (e.g. confidence/credible interval), ideally using structured tables or plots.	Table 1<br>Results
Results of syntheses	20a	For each synthesis, briefly summarise the characteristics and risk of bias among contributing studies.	Results<br>Supplements
	20b	Present results of all statistical syntheses conducted. If meta-analysis was done, present for each the summary estimate and its precision (e.g. confidence/credible interval) and measures of statistical heterogeneity. If comparing groups, describe the direction of the effect.	N/A
	20c	Present results of all investigations of possible causes of heterogeneity among study results.	Discussion<br>Limitations and strengths
	20d	Present results of all sensitivity analyses conducted to assess the robustness of the synthesized results.	N/A
Reporting biases	21	Present assessments of risk of bias due to missing results (arising from reporting biases) for each synthesis assessed.	Supplements<br>Limitations and strengths
Certainty of evidence	22	Present assessments of certainty (or confidence) in the body of evidence for each outcome assessed.	Supplements<br>Results
**DISCUSSION**			
Discussion	23a	Provide a general interpretation of the results in the context of other evidence.	Discussion
	23b	Discuss any limitations of the evidence included in the review.	Limitations and strengths
	23c	Discuss any limitations of the review processes used.	Limitations and strengths
	23d	Discuss implications of the results for practice, policy, and future research.	Conclusions
**OTHER INFORMATION**			
Registration and protocol	24a	Provide registration information for the review, including register name and registration number, or state that the review was not registered.	N/A
	24b	Indicate where the review protocol can be accessed, or state that a protocol was not prepared.	N/A
	24c	Describe and explain any amendments to information provided at registration or in the protocol.	N/A
Support	25	Describe sources of financial or non-financial support for the review, and the role of the funders or sponsors in the review.	Funding
Competing interests	26	Declare any competing interests of review authors.	Disclosure statement
Availability of data, code and other materials	27	Report which of the following are publicly available and where they can be found: template data collection forms; data extracted from included studies; data used for all analyses; analytic code; any other materials used in the review.	Supplements

From: Page MJ, McKenzie JE, Bossuyt PM, Boutron I, Hoffmann TC, Mulrow CD, et al. The PRISMA 2020 statement: an updated guideline for reporting systematic reviews. BMJ 2021;372:n71. doi: 10.1136/bmj.n71

For more information, visit: http://www.prisma-statement.org/

Supplementary Table 1. Table of Included/excluded studies

	Search from PUBMED/MEDLINE	
1.	Front Pediatr. 2023 Sep 21;11:1170379. doi: 10.3389/fped.2023.1170379. eCollection 2023. Estimate of the incidence of PANDAS and PANS in 3 primary care populations. Wald ER , Eickhoff J , Flood GE , Heinz MV , Liu D , Agrawal A , Morse RP , Raney VM , Veerapandiyan A, Madan JC.	Unrelated
**2.**	**Psychiatry Res. 2023 Oct;328:115490. doi: 10.1016/j.psychres.2023.115490. Epub 2023 Sep 21.Delusional beliefs in adolescents with anorexia nervosa, obsessive-compulsive disorder, or first-episode psychosis: A comparative study. Camprodon-Boadas P , De la Serna E , Plana MT , Flamarique I , Lázaro L , Borràs R , Baeza I , Tasa-Vinyals E , Sugranyes G , Ortiz AE , Castro-Fornieles J .**	**Included**
3.	BMC Psychiatry. 2023 Jul 31;23 :551. doi: 10.1186/s12888-023-05039-6. "A ghost doesn't need insulin," Cotard's delusion leading to diabetic ketoacidosis and a body-mass index of 15: a case presentation. Robertson C , Dunn T .	Unrelated
4.	Front Psychiatry. 2023 Apr 27;14:966935. doi: 10.3389/fpsyt.2023.966935. eCollection 2023. Case report: Anorexia nervosa and unspecified restricting-type eating disorder in Jewish ultra-orthodox religious males, leading to severe physical and psychological morbidity. Laufer S , Herman E , Serfaty D , Latzer Y , Ashkenazi R , Attias O , Oren S , Shimomi M , Uziel M , Enoch-Levy A , Witztum E , Stein D .	Case Report
5.	J Clin Med. 2022 Oct 18;11 :6134. doi: 10.3390/jcm11206134.Orthorexia Nervosa, Eating Disorders, and Obsessive-Compulsive Disorder: A Selective Review of the Last Seven Years. Pontillo M , Zanna V , Demaria F , Averna R , Di Vincenzo C , De Biase M , Di Luzio M , Foti B , Tata MC , Vicari S .	Review
6.	Diabetes Metab. 2023 Jan;49 :101392. doi: 10.1016/j.diabet.2022.101392. Epub 2022 Oct 1.Parental history of psychiatric disorders and risk of type 1 diabetes in the offspring.Yin W , Persson M , Sandin S .	Unrelated
7.	Mol Psychiatry. 2022 Dec;27 :5244-5254. doi: 10.1038/s41380-022-01753-x. Epub 2022 Aug 30.Paternal age and 13 psychiatric disorders in the offspring: a population-based cohort study of 7 million children in Taiwan. Wang SH , Wu CS , Hsu LY , Lin MC , Chen PC , Thompson WK , Fan CC .	Lumping
**8.**	**Int J Eat Disord. 2022 Oct;55 :1342-1351. doi: 10.1002/eat.23782. Epub 2022 Jul 21. Characterizing changes in obsessive-compulsive symptoms over the course of treatment for adolescent bulimia nervosa. Reilly EE , Gorrell S , Brosof L , Lock J , Le Grange D .**	**Included**
9.	Nutr Hosp. 2022 Aug 26;39(Spec No2):8-15. doi: 10.20960/nh.04173. [Characterization, epidemiology and trends of eating disorders].[Article in Spanish] Arija Val V , Santi Cano MJ , Novalbos Ruiz JP , Canals J , Rodríguez Martín A .	Review
10.	World J Psychiatry. 2022 Mar 19;12 :425-449. doi: 10.5498/wjp.v12.i3.425. eCollection 2022 Mar 19. Clinical high-risk criteria of psychosis in 8-17-year-old community subjects and inpatients not suspected of developing	Unrelated

	psychosis. Schultze-Lutter F , Walger P , Franscini M , Traber-Walker N , Osman N , Walger H , Schimmelmann BG , Flückiger R , Michel C .	
11.	J Am Acad Child Adolesc Psychiatry. 2022 Jul;61 :934-945. doi: 10.1016/j.jaac.2021.11.035. Epub 2022 Apr 1. Genome-wide Association Meta-analysis of Childhood and Adolescent Internalizing Symptoms. Jami ES , Hammerschlag AR , Ip HF , Allegrini AG , Benyamin B , Border R , Diemer EW , Jiang C , Karhunen V , Lu Y , Lu Q , Mallard TT , Mishra PP , Nolte IM , Palviainen T , Peterson RE , Sallis HM , Shabalin AA , Tate AE , Thiering E , Vilor-Tejedor N , Wang C , Zhou A , Adkins DE , Alemany S , Ask H , Chen Q , Corley RP , Ehli EA , Evans LM , Havdahl A , Hagenbeek FA , Hakulinen C , Henders AK, Hottenga JJ , Korhonen T , Mamun A, Marrington S, Neumann A, Rimfeld K, Rivadeneira F, Silberg JL , van Beijsterveldt CE , Vuoksimaa E , Whipp AM , Tong X , Andreassen OA, Boomsma DI , Brown SA, Burt SA , Copeland W, Dick DM, Harden KP , Harris KM, Hartman CA , Heinrich J, Hewitt JK , Hopfer C, Hypponen E , Jarvelin MR, Kaprio J , Keltikangas-Järvinen L , Klump KL , Krauter K, Kuja-Halkola R , Larsson H , Lehtimäki T , Lichtenstein P , Lundström S, Maes HH, Magnus P, Munafò MR, Najman JM, Njølstad PR, Oldehinkel AJ , Pennell CE , Plomin R, Reichborn-Kjennerud T , Reynolds C, Rose RJ, Smolen A , Snieder H , Stallings M , Standl M, Sunyer J, Tiemeier H , Wadsworth SJ , Wall TL, Whitehouse AJO, Williams GM, Ystrøm E, Nivard MG , Bartels M , Middeldorp CM.	Unrelated
12.	Int J Eat Disord. 2022 May;55 :664-677. doi: 10.1002/eat.23705. Epub 2022 Mar 18.In pursuit of biomarkers for predicting susceptibility to activity-based anorexia in adolescent female rats. Milton LK , Patton T , O'Keeffe M , Oldfield BJ , Foldi CJ .	Animal
13.	Psychol Med. 2023 May;53 :3021-3035. doi: 10.1017/S0033291721005079. Epub 2022 Mar 4.Predicting eating disorder and anxiety symptoms using disorder-specific and transdiagnostic polygenic scores for anorexia nervosa and obsessive-compulsive disorder. Yilmaz Z , Schaumberg K , Halvorsen M , Goodman EL , Brosof LC , Crowley JJ ; Anorexia Nervosa Genetics Initiative; Eating Disorders Working Group of the Psychiatric Genomics Consortium; Tourette Syndrome/Obsessive-Compulsive Disorder Working Group of the Psychiatric Genomics Consortium; Mathews CA , Mattheisen M , Breen G , Bulik CM , Micali N , Zerwas SC .	Lumping
14.	Transl Psychiatry. 2022 Feb 14;12 :59. doi: 10.1038/s41398-022-01817-0.Insulinopathies of the brain? Genetic overlap between somatic insulin-related and neuropsychiatric disorders.Fanelli G , Franke B , De Witte W , Ruisch IH , Haavik J , van Gils V , Jansen WJ , Vos SJB , Lind L , Buitelaar JK , Banaschewski T , Dalsgaard S , Serretti A , Mota NR , Poelmans G , Bralten J .	Lumping
15.	Front Psychiatry. 2022 Jan 11;12:803898. doi: 10.3389/fpsyt.2021.803898. eCollection 2021.Ehlers-Danlos Syndrome in the Field of Psychiatry: A Review. Ishiguro H , Yagasaki H , Horiuchi Y .	Review
16.	Eur Eat Disord Rev. 2022 Mar;30 :110-123. doi: 10.1002/erv.2885. Epub 2022 Jan 21. Bridging of childhood obsessive-compulsive personality disorder traits and adult eating disorder symptoms: A network analysis approach. Giles S , Hughes EK , Fuller-Tyszkiewicz M , Treasure J ,	Adult

	Fernandez-Aranda F , Karwautz AFK , Wagner G , Anderluh M , Collier DA , Krug I .	
17.	Front Psychiatry. 2021 Nov 11;12:703701. doi: 10.3389/fpsyt.2021.703701. eCollection 2021.Comorbidities in Obsessive-Compulsive Disorder Across the Lifespan: A Systematic Review and Meta-Analysis. Sharma E , Sharma LP , Balachander S , Lin B , Manohar H , Khanna P , Lu C , Garg K , Thomas TL , Au ACL , Selles RR , Højgaard DRMA , Skarphedinsson G , Stewart SE .	Review
18.	Front Genet. 2021 Aug 31;12:711624. doi: 10.3389/fgene.2021.711624. eCollection 2021. Polygenic Heterogeneity Across Obsessive-Compulsive Disorder Subgroups Defined by a Comorbid Diagnosis. Strom NI , Grove J , Meier SM , Bækvad-Hansen M , Becker Nissen J , Damm Als T , Halvorsen M , Nordentoft M , Mortensen PB , Hougaard DM , Werge T , Mors O , Børglum AD , Crowley JJ , Bybjerg-Grauholm J , Mattheisen M .	No ED
19.	Front Psychiatry. 2021 Jul 1;12:658416. doi: 10.3389/fpsyt.2021.658416. eCollection 2021.Is the Severity of the Clinical Expression of Anorexia Nervosa Influenced by an Anxiety, Depressive, or Obsessive-Compulsive Comorbidity Over a Lifetime? Riquin E , Raynal A , Mattar L , Lalanne C , Hirot F , Huas C , Duclos J , Berthoz S ; EVHAN group; Godart N .	Adult
20.	J Child Psychol Psychiatry. 2022 May;63 :519-526. doi: 10.1111/jcpp.13484. Epub 2021 Jul 5. Psychiatric comorbidity of eating disorders in children between the ages of 9 and 10.Convertino AD , Blashill AJ .	No OCD
21.	J Psychiatr Res. 2021 Sep;141:176-191. doi: 10.1016/j.jpsychires.2021.06.035. Epub 2021 Jun 19. Comorbid obsessive-compulsive disorder in individuals with eating disorders: An epidemiological meta-analysis. Drakes DH , Fawcett EJ , Rose JP , Carter-Major JC , Fawcett JM .	Review
22.	Eat Weight Disord. 2022 Apr;27 :1193-1207. doi: 10.1007/s40519-021-01252-y. Epub 2021 Jun 30. Obsessive-compulsive symptoms in young women affected with anorexia nervosa, and their relationship with personality, psychopathology, and attachment style. Amianto F , Secci I , Arletti L , Davico C , Abbate Daga G , Vitiello B .	Adult
23.	Int J Eat Disord. 2021 Sep;54 :1608-1618. doi: 10.1002/eat.23570. Epub 2021 Jun 18. Comorbid mental disorders during long-term course in a nationwide cohort of patients with anorexia nervosa. Steinhausen HC , Villumsen MD , Hørder K , Winkler LA , Bilenberg N , Støving RK .	Adult
24.	Mol Psychiatry. 2022 Jan;27 :281-295. doi: 10.1038/s41380-021-01161-7. Epub 2021 Jun 2. Age at onset of mental disorders worldwide: large-scale meta-analysis of 192 epidemiological studies.Solmi M , Radua J , Olivola M , Croce E , Soardo L , Salazar de Pablo G , Il Shin J , Kirkbride JB , Jones P , Kim JH , Kim JY , Carvalho AF , Seeman MV , Correll CU , Fusar-Poli P .	Review
25.	World Psychiatry. 2021 Jun;20 :244-275. doi: 10.1002/wps.20881.Efficacy and acceptability of pharmacological, psychosocial, and brain stimulation interventions in children and adolescents with mental disorders: an umbrella	Review

	review. Correll CU , Cortese S , Croatto G , Monaco F , Krinitski D , Arrondo G , Ostinelli EG , Zangani C , Fornaro M , Estradé A , Fusar-Poli P , Carvalho AF , Solmi M .	
26.	Eur Eat Disord Rev. 2021 Jul;29 :622-633. doi: 10.1002/erv.2828. Epub 2021 Apr 13.Motivation to recover for adolescent and adult eating disorder patients in residential treatment. Manwaring J , Blalock DV , Le Grange D , Duffy A , McClanahan SF , Johnson C , Mehler PS , Plotkin M , Rienecke RD .	No OCD
27.	Ital J Pediatr. 2021 Mar 9;47 :59. doi: 10.1186/s13052-021-01006-7.Autism spectrum disorder and anorexia nervosa: an Italian prospective study. Pruccoli J , Solari A , Terenzi L , Malaspina E , Angotti M , Pignataro V , Gualandi P , Sacrato L , Cordelli DM , Franzoni E , Parmeggiani A .	No OCD
28.	Arch Womens Ment Health. 2021 Aug;24 :671-680. doi: 10.1007/s00737-021-01120-9. Epub 2021 Mar 18. 2019-nCOV distress and depressive, anxiety and OCD-type, and eating disorder symptoms among postpartum and control women. Thompson KA , Bardone-Cone AM .	Adult
29.	Neuropeptides. 2021 Apr;86:102133. doi: 10.1016/j.npep.2021.102133. Epub 2021 Feb 17. Omentin and visfatin in adolescent inpatients with anorexia nervosa; association with symptoms. Tyszkiewicz-Nwafor M , Dutkiewicz A , Paszynska E , Dmitrzak-Weglarz M , Slopien A .	Unrelated
30.	Lancet Digit Health. 2021 Apr;3 :e217-e230. doi: 10.1016/S2589-7500 00017-0. Epub 2021 Feb 18. Indirect acute effects of the COVID-19 pandemic on physical and mental health in the UK: a population-based study. Mansfield KE , Mathur R , Tazare J , Henderson AD , Mulick AR , Carreira H , Matthews AA , Bidulka P , Gayle A , Forbes H , Cook S , Wong AYS , Strongman H , Wing K , Warren-Gash C , Cadogan SL , Smeeth L , Hayes JF , Quint JK , McKee M , Langan SM .	Unrelated
31.	Eat Behav. 2021 Jan;40:101476. doi: 10.1016/j.eatbeh.2021.101476. Epub 2021 Feb 2. The interaction of negative psychological well-being and picky eating in relation to disordered eating in undergraduate students. Barnhart WR , Hamilton L , Jordan AK , Pratt M , Musher-Eizenman DR .	Unrelated
32.	Nutrients. 2021 Jan 27;13 :403. doi: 10.3390/nu13020403. Health-Related Quality of Life in Spanish Women with Eating Disorders. Panea-Pizarro I , Moran JM , Lavado-García J , Beato-Fernández L , Domínguez-Martin AT , Huerta-González S , Novo A , Pedrera-Zamorano JD , López-Espuela F .	Adult
33.	J Cogn Psychother. 2020 Nov 1;34 :280-305. doi: 10.1891/JCPSY-D-20-00001. Acceptance and Commitment Therapy: A Systematic Literature Review of Prevention and Intervention Programs for Mental Health Difficulties in Children and Young People.Harris E , Samuel V .	Review
34.	Nutrients. 2020 Nov 13;12 :3490. doi: 10.3390/nu12113490. Identifying the Profile of Orthorexic Behavior and "Normal" Eating Behavior with Cluster Analysis: A Cross-Sectional Study among Polish Adults. Brytek-Matera A , Staniszewska A , Hallit S .	Adult
35.	J Am Coll Health. 2022 Oct;70 :1975-1980. doi: 10.1080/07448481.2020.1841769. Epub 2020 Nov 12. Are the obsessive-compulsive traits a moderator for the relationship between autism and	Adult

	anorexia? A cross-sectional study among university students.Acikel SB , Cikili Y .	
36.	Alcohol Clin Exp Res. 2020 Dec;44 :2536-2544. doi: 10.1111/acer.14477. Epub 2020 Nov 5.Associations Between Psychiatric Disorders and Alcohol Consumption Levels in an Adult Primary Care Population.Palzes VA , Parthasarathy S , Chi FW , Kline-Simon AH , Lu Y , Weisner C , Ross TB , Elson J , Sterling SA .	No OCD
37.	Int J Eat Disord. 2020 Nov;53 :1844-1854. doi: 10.1002/eat.23366. Epub 2020 Aug 26. Increased rates of eating disorders and their symptoms in women with major depressive disorder and anxiety disorders. Garcia SC , Mikhail ME , Keel PK , Burt SA , Neale MC , Boker S , Klump KL .	No OCD
38.	Int J Eat Disord. 2020 Oct;53 :1636-1646. doi: 10.1002/eat.23355. Epub 2020 Aug 9. Cognitive-behavioral therapy for avoidant/restrictive food intake disorder: Feasibility,acceptability, and proof-of-concept for children and adolescents.Thomas JJ , Becker KR , Kuhnle MC , Jo JH , Harshman SG , Wons OB , Keshishian AC , Hauser K , Breithaupt L , Liebman RE , Misra M , Wilhelm S , Lawson EA , Eddy KT .	No OCD
39.	Epidemiol Psychiatr Sci. 2020 May 26;29:e131. doi: 10.1017/S2045796020000451. Risk of eating disorders in international adoptees: a cohort study using Swedish national population registers. Strand M , Zhang R , Thornton LM , Birgegård A , D'Onofrio BM , Bulik CM .	Adult
40.	Eat Behav. 2020 Apr;37:101386. doi: 10.1016/j.eatbeh.2020.101386. Epub 2020 Apr 16. Obsessive compulsive disorder and thought action fusion: Relationships with eating disorder outcomes. Lee EB , Barney JL , Twohig MP , Lensegrav-Benson T , Quakenbush B .	Adult
41.	J Neural Transm (Vienna). 2020 Nov;127 :1547-1568. doi: 10.1007/s00702-020-02197-9. Epub 2020 May 7.Mental health dished up-the use of iPSC models in neuropsychiatric research. McNeill RV , Ziegler GC , Radtke F , Nieberler M , Lesch KP , Kittel-Schneider S.	Review
42.	Eat Weight Disord. 2021 Mar;26 :667-677. doi: 10.1007/s40519-020-00906-7. Epub 2020 Apr 30. Personality disorder traits, obsessive ideation and perfectionism 20 years after adolescent-onset anorexia nervosa: a recovered study. Gárriz M , Andrés-Perpiñá S , Plana MT , Flamarique I , Romero S , Julià L , Castro-Fornieles J .	No OCD
43.	Gene. 2020 Jul 20;748:144675. doi: 10.1016/j.gene.2020.144675. Epub 2020 Apr 18.m Sequence analysis of five exons of SLC6A4 gene in Mexican patients with anorexia nervosa and bulimia nervosa. Hernández-Muñoz S , Camarena-Medellin B , González Macías L , Aguilar-García A , Flores-Flores G , Luna Dominguez D , Azaola-Espinosa A , Flores-Ramos M , Caballero-Romo A .	No OCD
44.	J Med Case Rep. 2020 Apr 17;14 :49. doi: 10.1186/s13256-020-02371-3. An adolescent girl with signs and symptoms of anaphylaxis and negative immunologic workup: a case report. Green S , Hicks A , Hilsendager C , Bauer M , Frank GKW .	Unrelated
45.	Ann Ital Chir. 2020 Mar 9;9:S2239253X20032442. Paediatric gastric trichobezoar: the Rapunzel syndrome A report of two cases. Tripodi V,	Unrelated

	Caruso A, Luraghi M, Armao FT, Bisagni P, Romano S, Scagnelli P, Giacchero R, Gendarini A, Leoni P.	
46.	Am J Psychiatry. 2020 Feb 1;177 :134-142. doi: 10.1176/appi.ajp.2019.19020184. Randomized Controlled Trial Comparing Health Coach-Delivered Smartphone-Guided Self-Help With Standard Care for Adults With Binge Eating. Hildebrandt T , Michaeledes A , Mayhew M , Greif R , Sysko R , Toro-Ramos T , DeBar L .	Unrelated
47.	Psychol Med. 2021 Apr;51 :870-880. doi: 10.1017/S0033291719003945. Epub 2020 Jan 8.Prenatal and perinatal factors and risk of eating disorders. Larsen JT , Bulik CM , Thornton LM , Koch SV , Petersen L .	Lumping
48.	Nat Commun. 2019 Dec 18;10 :5765. doi: 10.1038/s41467-019-13544-0. Genetic correlations of psychiatric traits with body composition and glycemic traits are sex- and age-dependent. Hübel C , Gaspar HA , Coleman JRI , Hanscombe KB , Purves K , Prokopenko I , Graff M , Ngwa JS , Workalemahu T ; ADHD Working Group of the Psychiatric Genomics Consortium; Meta-Analyses of Glucose and Insulin-related traits consortium (MAGIC); Autism Working Group of the Psychiatric Genomics Consortium; Bipolar Disorder Working Group of the Psychiatric Genomics Consortium; Eating Disorders Working Group of the Psychiatric Genomics Consortium; Major Depressive Disorder Working Group of the Psychiatric Genomics Consortium; OCD & Tourette Syndrome Working Group of the Psychiatric Genomics Consortium; PTSD Working Group of the Psychiatric Genomics Consortium; Schizophrenia Working Group of the Psychiatric Genomics Consortium; Sex Differences Cross Disorder Working Group of the Psychiatric Genomics Consortium; Substance Use Disorders Working Group of the Psychiatric Genomics Consortium; German Borderline Genomics Consortium; International Headache Genetics Consortium; O'Reilly PF , Bulik CM , Breen G .	Adult
49.	Int J Eat Disord. 2020 Mar;53 :349-361. doi: 10.1002/eat.23197. Epub 2019 Nov 19. Prevalence, correlates and comorbidities of feeding and eating disorders in a nationally representative sample of Iranian children and adolescents. Mohammadi MR , Mostafavi SA , Hooshyari Z , Khaleghi A , Ahmadi N , Molavi P , Armani Kian A , Safavi P , Delpisheh A , Talepasand S , Hojjat SK , Pourdehghan P , Ostovar R , Hosseini SH , Mohammadzadeh S , Salmanian M , Alavi SS , Ahmadi A , Zarafshan H .	No OCD
**50.**	**J Can Acad Child Adolesc Psychiatry. 2019 Aug;28 :45-54. Epub 2019 Aug 1. Comparison of Perfectionism Dimensions in Adolescents with Anorexia Nervosa or Obsessive-Compulsive Disorder. Flamarique I , Plana MT , Castro-Fornieles J , Borràs R , Moreno E , Lázaro L .**	**Included**
51.	Psychiatr Pol. 2019 Apr 30;53 :371-382. doi: 10.12740/PP/OnlineFirst/85729. Epub 2019 Apr 30. Orthorexia nervosa - a separate clinical entity, a part of eating disorder spectrum or another manifestation of obsessive-compulsive disorder? [Article in English, Polish] Łucka I , Janikowska-Hołoweńko D , Domarecki P , Plenikowska-Ślusarz T , Domarecka M	No OCD
52.	Eat Behav. 2019 Aug;34:101298. doi: 10.1016/j.eatbeh.2019.05.001. Epub 2019 May 31. Obsessions are strongly related to eating disorder symptoms	Adult

	in anorexia nervosa and atypical anorexia nervosa. Levinson CA , Brosof LC , Ram SS , Pruitt A , Russell S , Lenze EJ .	
**53.**	**Eur Child Adolesc Psychiatry. 2020 Feb;29 :217-226. doi: 10.1007/s00787-019-01350-4. Epub 2019 May 21. Performance and brain activity during the Wisconsin Card Sorting Test in adolescents with obsessive-compulsive disorder and adolescents with weight-restored anorexia nervosa. Bohon C , Weinbach N , Lock J .**	**Included**
54.	Eat Weight Disord. 2020 Jun;25 :821-826. doi: 10.1007/s40519-019-00694-9. Epub 2019 Apr 25. Clinical and socio-demographic features in childhood vs adolescent-onset anorexia nervosa in an Asian population. Kwok C , Kwok V , Lee HY , Tan SM .	No OCD
55.	Asian J Psychiatr. 2019 Apr;42:94-103. doi: 10.1016/j.ajp.2019.03.029. Epub 2019 Mar 28. Meningioma and psychiatric symptoms: An individual patient data analysis. Gyawali S , Sharma P , Mahapatra A .	Review
56.	J Behav Ther Exp Psychiatry. 2019 Sep;64:113-122. doi: 10.1016/j.jbtep.2019.03.007. Epub 2019 Mar 30. A randomised controlled trial of guided internet-based cognitive behavioural therapy for perfectionism: Effects on psychopathology and transdiagnostic processes. Kothari R , Barker C , Pistrang N , Rozental A , Egan S , Wade T , Allcott-Watson H , Andersson G , Shafran R .	Adult
57.	Minerva Pediatr. 2020 Dec;72 :501-507. doi: 10.23736/S0026-4946.19.05202-2. Epub 2019 Feb 13. A glance into psychiatric comorbidity in adolescents with anorexia nervosa. Catone G , Pisano S , Muzzo G , Corrado G , Russo K , Maiorano A , Salerno F , Gritti A.	No OCD
58.	Psychiatry Res. 2019 Jul;277:23-38. doi: 10.1016/j.psychres.2019.01.026. Epub 2019 Jan 8.Transdiagnostic neuroimaging in psychiatry: A review. Mitelman SA.	Review
59.	Am J Med Genet B Neuropsychiatr Genet. 2019 Sep;180 :428-438. doi: 10.1002/ajmg.b.32709. Epub 2018 Dec 28.Genomics of body fat percentage may contribute to sex bias in anorexia nervosa. Hübel C , Gaspar HA , Coleman JRI , Finucane H , Purves KL , Hanscombe KB , Prokopenko I ; MAGIC investigators; Graff M , Ngwa JS , Workalemahu T ; Eating Disorders Working Group of the Psychiatric Genomics Consortium; Major Depressive Disorder Working Group of the Psychiatric Genomics Consortium; Schizophrenia Working Group of the Psychiatric Genomics Consortium; Tourette Syndrome/Obsessive-Compulsive Disorder Working Group of the Psychiatric Genomics Consortium; O'Reilly PF , Bulik CM , Breen G .	No OCD
60.	Brain Behav. 2019 Jan;9 :e01205. doi: 10.1002/brb3.1205. Epub 2018 Dec 27. Resting state connectivity within and across neural circuits in anorexia nervosa. Uniacke B , Wang Y , Biezonski D , Sussman T , Lee S , Posner J , Steinglass J .	No OCD
61.	J Am Coll Health. 2019 Nov-Dec;67 :801-816. doi: 10.1080/07448481.2018.1515745. Epub 2018 Dec 20. Pilot study of a personality-based approach to assessing eating disorder and Obsessive Compulsive Disorder symptom risk in college men and women. Hellberg SN , Ladis IE , Shepherd CB .	Adult

62.	J Behav Addict. 2018 Dec 1;7 :1143-1156. doi: 10.1556/2006.7.2018.129. Epub 2018 Dec 17. Orthorexia nervosa: A behavioral complex or a psychological condition? Strahler J , Hermann A , Walter B , Stark R .	Adult
**63.**	**Psychiatry Res. 2019 Jan;271:554-558. doi: 10.1016/j.psychres.2018.12.019. Epub 2018 Dec 6. Genetic variability in the serotoninergic system and age of onset in anorexia nervosa and obsessive-compulsive disorder. Plana MT , Torres T , Rodríguez N , Boloc D , Gassó P , Moreno E , Lafuente A , Castro-Fornieles J , Mas S , Lazaro L .**	**Included**
64.	J Child Psychol Psychiatry. 2019 Jun;60 :686-696. doi: 10.1111/jcpp.12984. Epub 2018 Oct 24. Anxiety disorder symptoms at age 10 predict eating disorder symptoms and diagnoses in adolescence. Schaumberg K , Zerwas S , Goodman E , Yilmaz Z , Bulik CM , Micali N .	No OCD
65.	Am J Med Genet B Neuropsychiatr Genet. 2018 Oct;177 :641-657. Doi: 10.1002/ajmg.b.32652. Epub 2018 Oct 16.Genetic correlations among psychiatric and immune-related phenotypes based on genome-wide association data. Tylee DS , Sun J , Hess JL , Tahir MA , Sharma E , Malik R , Worrall BB , Levine AJ , Martinson JJ , Nejentsev S , Speed D , Fischer A , Mick E , Walker BR , Crawford A , Grant SFA , Polychronakos C , Bradfield JP , Sleiman PMA , Hakonarson H , Ellinghaus E , Elder JT , Tsoi LC , Trembath RC , Barker JN , Franke A , Dehghan A ; 23 and Me Research Team ; Inflammation Working Group of the CHARGE Consortium; METASTROKE Consortium of the International Stroke Genetics Consortium; Netherlands Twin Registry; neuroCHARGE Working Group; Obsessive Compulsive and Tourette Syndrome Working Group of the Psychiatric Genomics Consortium; Faraone SV , Glatt SJ .	Lumping
**66.**	**World J Biol Psychiatry. 2019 Nov;20 :723-731. doi: 10.1080/15622975.2018.1492735. Epub 2018 Sep 28. Adiponectin and resistin in acutely ill and weight-recovered adolescent anorexia nervosa: Association with psychiatric symptoms. Tyszkiewicz-Nwafor M , Slopien A , Dmitrzak-Węglarz M , Rybakowski F .**	**Included**
**67.**	**Eur Eat Disord Rev. 2019 May;27 :224-235. doi: 10.1002/erv.2638. Epub 2018 Sep 9. Obsessive-compulsive symptomatology in female adolescent inpatients with restrictive compared with binge-purge eating disorders.Lewis YD , Gilon Mann T , Enoch-Levy A , Dubnov-Raz G , Gothelf D , Weizman A , Stein D .**	**Included**
68.	Eur Eat Disord Rev. 2019 Mar;27 :161-172. doi: 10.1002/erv.2635. Epub 2018 Aug 22. Associations between dimensions of anorexia nervosa and obsessive-compulsive disorder: An examination of personality and psychological factors in patients with anorexia nervosa. Levinson CA , Zerwas SC , Brosof LC , Thornton LM , Strober M , Pivarunas B , Crowley JJ , Yilmaz Z , Berrettini WH , Brandt H , Crawford S , Fichter MM , Halmi KA , Johnson C , Kaplan AS , La Via M , Mitchell J , Rotondo A , Woodside DB , Kaye WH , Bulik CM .	Adult
69.	Mol Psychiatry. 2020 Sep;25 :2036-2046. doi: 10.1038/s41380-018-0115-4. Epub 2018 Aug 7. Examination of the shared genetic basis of anorexia nervosa and obsessive-compulsive disorder. Yilmaz Z(#) , Halvorsen M(#) ,	Adult

	Bryois J , Yu D , Thornton LM , Zerwas S , Micali N , Moessner R , Burton CL , Zai G , Erdman L , Kas MJ , Arnold PD , Davis LK , Knowles JA , Breen G , Scharf JM , Nestadt G , Mathews CA(#) , Bulik CM(#) , Mattheisen M(#) , Crowley JJ(#) (28); Eating Disorders Working Group of the Psychiatric Genomics Consortium, Tourette Syndrome/Obsessive–Compulsive Disorder Working Group of the Psychiatric Genomics Consortium.	
70.	Compr Psychiatry. 2018 Oct;86:67-73. doi: 10.1016/j.comppsych.2018.07.013. Epub 2018 Jul 29. Dual diagnosis of obsessive compulsive and compulsive buying disorders: Demographic, clinical, and psychiatric correlates. Kim HS , Hodgins DC , Torres AR , Fontenelle LF , do Rosário MC , de Mathis MA , Ferrão YA , Miguel EC , Tavares H .	Adult
71.	Eat Weight Disord. 2019 Feb;24 :29-35. doi: 10.1007/s40519-018-0527-9. Epub 2018 Jun 22. People behind unhealthy obsession to healthy food: the personality profile of tendency to orthorexia nervosa. Kiss-Leizer M , Rigó A .	Unrelated
72.	Gene. 2018 May 15;654:43-48. doi: 10.1016/j.gene.2018.02.035. Epub 2018 Feb 15. Effect of dopamine receptor D4 (DRD4) haplotypes on general psychopathology in patients with eating disorders. Gervasini G , González LM , Gamero-Villarroel C , Mota-Zamorano S , Carrillo JA , Flores I , García-Herráiz A .	Unrelated
73.	Eat Weight Disord. 2018 Apr;23 :177-184. doi: 10.1007/s40519-017-0464-z. Epub 2018 Jan 8. Eating disorders, substance use disorders and multiple symptoms: three clinical vignettes. Fava Vizziello G , Bellin L .	No OCD
74.	J Clin Med Res. 2017 Dec;9 :1026-1028. doi: 10.14740/jocmr3195w. Epub 2017 Nov 6. Obsessive-Compulsive Disorder in a 19-Year-Old Female Adolescent With Turner Syndrome. Moonga SS , Pinkhasov A , Singh D .	Case Report
75.	Encephale. 2018 Nov;44 :429-434. doi: 10.1016/j.encep.2017.07.005. [The psychiatric comorbidity of anorexia nervosa: A comparative study in a population of French and Greek anorexic patients]. [Article in French] Kountza M , Garyfallos G , Ploumpidis D , Varsou E , Gkiouzepas I .	Adult
76.	Psicothema. 2017 Nov;29 :433-439. doi: 10.7334/psicothema2016.372. Intensive family exposure-based cognitive-behavioral treatment for adolescents with anorexia nervosa. Iniesta Sepúlveda M , Nadeau JM, Whelan MK, Oiler CM, Ramos A, Riemann BC, Storch EA.	No OCD
77.	J Affect Disord. 2018 Jan 1;225:429-437. doi: 10.1016/j.jad.2017.08.062. Epub 2017 Aug 23. The classification of body dysmorphic disorder symptoms in male and female adolescents. Schneider SC , Baillie AJ , Mond J , Turner CM , Hudson JL .	Unrelated
78.	J Can Acad Child Adolesc Psychiatry. 2017 Winter;26 :59-61. Epub 2017 Mar 1. Fear of Vomiting and Low Body Weight in Two Pediatric Patients: Diagnostic Challenges. Maertens C , Couturier J , Grant C , Johnson N .	Case Report
79.	Front Psychiatry. 2017 Jan 19;8:3. doi: 10.3389/fpsyt.2017.00003. eCollection 2017. Temporal Association of Certain Neuropsychiatric Disorders Following Vaccination of Children and Adolescents: A Pilot Case-	Unrelated

	Control Study. Leslie DL , Kobre RA , Richmand BJ , Aktan Guloksuz S , Leckman JF .	
80.	Z Kinder Jugendpsychiatr Psychother. 2017 Sep;45 :371-380. doi:10.1024/1422 4917/a000493. Epub 2016 Dec 12. Cognitive Flexibility in Juvenile Anorexia Nervosa in Relation to Comorbid Symptoms of Depression, Obsessive Compulsive Symptoms and Duration of Illness. Rößner A , Juniak I , van Noort BM , Pfeiffer E , Lehmkuhl U , Kappel V .	No OCD
81.	J Physiol Biochem. 2017 May;73 :297-305. doi: 10.1007/s13105-016-0540-2. Epub 2016 Dec 6. Neurobiochemical and psychological factors influencing the eating behaviors and attitudes in anorexia nervosa. Grzelak T , Dutkiewicz A , Paszynska E , Dmitrzak-Weglarz M , Slopien A , Tyszkiewicz-Nwafor M .	No OCD
82.	BMC Psychiatry. 2016 Nov 10;16 :393. doi: 10.1186/s12888-016-1109-x. Cognitive remediation therapy (CRT) as a treatment enhancer of eating disorders and obsessive compulsive disorders: study protocol for a randomized controlled trial. van Passel B , Danner U , Dingemans A , van Furth E , Sternheim L , van Elburg A , van Minnen A , van den Hout M , Hendriks GJ , Cath D .	Adult
83.	Trials. 2016 Nov 4;17 :532. doi: 10.1186/s13063-016-1663-z. Guided self-help interventions for mental health disorders in children with neurological conditions: study protocol for a pilot randomised controlled trial. Bennett S , Heyman I , Coughtrey A , Simmonds J , Varadkar S , Stephenson T , DeJong M , Shafran R .	Unrelated
84.	J Assoc Physicians India. 2015 Sep;63 :82-3. Anorexia Nervosa with Obsessive-Compulsive Disorder. Pani A , Santra G , Biswas KD .	Case Report
85.	Neuropsychiatr Dis Treat. 2016 Jul 7;12:1651-60. doi: 10.2147/NDT.S108912. eCollection 2016. Historical evolution of the concept of anorexia nervosa and relationships with orthorexia nervosa, autism, and obsessive-compulsive spectrum. Dell'Osso L , Abelli M , Carpita B , Pini S , Castellini G , Carmassi C , Ricca V .	Review
86.	JMIR Ment Health. 2016 Jul 5;3 :e29. doi: 10.2196/mental.5363. Online Obsessive-Compulsive Disorder Treatment: Preliminary Results of the "OCD? Not Me!" Self-Guided Internet-Based Cognitive Behavioral Therapy Program for Young People. Rees CS , Anderson RA, Kane RT, Finlay-Jones AL.	No ED
87.	Appetite. 2016 Oct 1;105:312-9. doi: 10.1016/j.appet.2016.06.004. Epub 2016 Jun 4. Predictors of binge eating in male and female youths in the United Arab Emirates. Schulte SJ .	Lumping
88.	Psychiatr Pol. 2016;50 :55-64. doi: 10.12740/PP/34810. Obsessive-compulsive symptoms and physical activity in patients with anorexia nervosa - possible relationships. [Article in English, Polish] Błachno M , Bryńska A , Tomaszewicz-Libudzic C , Jagielska G , Srebnicki T , Wiśniewski A , Wolańczyk T .	No OCD

89.	J Psychiatr Res. 2016 Jul;78:57-64. doi: 10.1016/j.jpsychires.2016.03.011. Epub 2016 Mar 26. Full spectrum of mental disorders linked with childhood residential mobility. Mok PL , Webb RT , Appleby L , Pedersen CB .	Unrelated
90.	Clin Neuropsychol. 2016 Feb;30 :228-42. doi: 10.1080/13854046.2016.1147603. Epub 2016 Mar 9. Planning Abilities in Patients with Anorexia Nervosa Compared with Healthy Controls. Carral-Fernández L , González-Blanch C , Goddard E , González-Gómez J , Benito-González P , Bustamante-Cruz E , Gómez Del Barrio A .	Adult
91.	J Neural Transm (Vienna). 2015 Nov;122 :1563-71. doi: 10.1007/s00702-015-1435-z. Epub 2015 Aug 2. Is nocturnal eating in restless legs syndrome linked to a specific psychopathological profile? A pilot study. Marconi S , Scarlatti F , Rizzo G , Antelmi E , Innamorati M , Pompili M , Brugnoli R , Belvederi Murri M , Amore M , Provini F .	Adult
92.	J Child Adolesc Psychopharmacol. 2015 Jun;25 :444-7. doi: 10.1089/cap.2015.29006.bjc. Pharmacotherapeutic Considerations in the Treatment of an Adolescent with Anorexia Nervosa and Obsessive Compulsive Disorder. Rice T , Coffey BJ .	Case Report
93.	Int J Eat Disord. 2015 Nov;48 :994-1004. doi: 10.1002/eat.22407. Epub 2015 Jun Validation of the yale-brown obsessive compulsive scale modified for binge eating. Deal LS , Wirth RJ , Gasior M , Herman BK , McElroy SL .	No OCD
94.	Psychopharmacology (Berl). 2015 Sep;232 :3173-81. doi: 10.1007/s00213-015-3967-1. Epub 2015 May 29.Developmental emergence of an obsessive-compulsive phenotype and binge behavior in rats. Freund N , Thompson BS, Norman KJ, Einhorn P, Andersen SL.	Animal
95.	J Med Internet Res. 2015 Feb 26;17 :e55. doi: 10.2196/jmir.4143. Multiple comorbidities of 21 psychological disorders and relationships with psychosocial variables: a study of the online assessment and diagnostic system within a web-based population. Al-Asadi AM , Klein B, Meyer D.	Unrelated
96.	Mol Autism. 2014 Dec 20;5 :56. doi: 10.1186/2040-2392-5-56. eCollection 2014. An examination of autism spectrum traits in adolescents with anorexia nervosa and their parents. Rhind C , Bonfioli E , Hibbs R , Goddard E , Macdonald P , Gowers S , Schmidt U , Tchanturia K , Micali N(#) , Treasure J(#) .	Lumping
97.	Eat Weight Disord. 2015 Jun;20 :161-6. doi: 10.1007/s40519-014-0171-y. Epub 2014 Dec 28. The prevalence of orthorexia nervosa among eating disorder patients after treatment. Segura-Garcia C , Ramacciotti C, Rania M, Aloi M, Caroleo M, Bruni A, Gazzarrini D, Sinopoli F, De Fazio P.	No OCD
**98.**	**Psychiatry Res. 2014 Dec 30;224 :246-53. doi: 10.1016/j.pscychresns.2014.10.001. Epub 2014 Oct 13. Obsessive-compulsivity and working memory are associated with differential prefrontal cortex and insula activation in adolescents with a recent diagnosis of an eating disorder. Brooks SJ , Solstrand Dahlberg L , Swenne I , Aronsson M , Zarei S , Lundberg L , Jacobsson JA , Rask-Andersen M , Salonen-Ros H , Rosling A , Larsson EM , Schiöth HB .**	**Included**
99.	J Child Adolesc Psychopharmacol. 2015 Feb;25 :48-56. doi: 10.1089/cap.2014.0063. Epub 2014 Oct 20. Disordered eating and food	No OCD

	restrictions in children with PANDAS/PANS.Toufexis MD , Hommer R, Gerardi DM, Grant P, Rothschild L, D'Souza P, Williams K, Leckman J, Swedo SE, Murphy TK.	
100.	Lakartidningen. 2014 Sep 23;111(39):1652-55. [ADHD should be given more attention--early interventions prevents unnecessary suffering]. [Article in Swedish] Fernell E , Nylander L , Kadesjö B , Gillberg C .	Unrelated
**101.**	**Eat Behav. 2014 Dec;15 :591-4. doi: 10.1016/j.eatbeh.2014.08.003. Epub 2014 Aug 23. Body checking as a behavioral link: a preliminary study assessing inhibition and its association to idiosyncratic body checking in anorexia nervosa. Breithaupt LE , Payne HA , Rose M .**	**Included**
102.	Psychiatr Pol. 2014 May-Jun;48 :429-39. [The influence of obsessive compulsive symptoms on the course of anorexia nervosa]. [Article in Polish] Błachno M, Bryńska A, Tomaszewicz-Libudzic C, Jagielska G, Srebnicki T, Wolańczyk T.	No English
103.	J Clin Psychiatry. 2014 Aug;75 :835-6. doi: 10.4088/JCP.13cr08917.Intranasal ketamine treatment in an adult with autism spectrum disorder. Wink LK , O'Melia AM, Shaffer RC, Pedapati E, Friedmann K, Schaefer T, Erickson CA.	Unrelated
104.	Transl Psychiatry. 2014 Sep 2;4(9):e432. doi: 10.1038/tp.2014.68. Genetic neuropathology of obsessive psychiatric syndromes. Jaffe AE , Deep-Soboslay A , Tao R , Hauptman DT(2), Kaye WH(3), Arango V(4), Weinberger DR(5), Hyde TM(6), Kleinman JE(6).	Lumping
105.	Eur Eat Disord Rev. 2014 Jul;22(4):237-42. doi: 10.1002/erv.2295. Epub 2014 May 23. Childhood obsessive-compulsive traits in anorexia nervosa patients, their unaffected sisters and healthy controls: a retrospective study. Degortes D , Zanetti T, Tenconi E, Santonastaso P, Favaro A.	No OCD
106.	PLoS One. 2014 May 20;9(5):e97998. doi: 10.1371/journal.pone.0097998. eCollection 2014. Provocation of symmetry/ordering symptoms in Anorexia nervosa: a functional neuroimaging study. Suda M , Brooks SJ(2), Giampietro V(3), Uher R , Mataix-Cols D(4), Brammer MJ(3), Williams SC(3), Treasure J , Campbell IC .	Adult
107.	Eur Eat Disord Rev. 2014 Jul;22(4):243-51. doi: 10.1002/erv.2286. Epub 2014 Feb 27. Risk factors and antecedent life events in the development of anorexia nervosa: a Portuguese case-control study. Machado BC , Gonçalves SF, Martins C, Hoek HW, Machado PP.	No OCD
108.	J Clin Psychiatry. 2014 Apr;75(4):393-8. doi: 10.4088/JCP.13m08646. The mental health characteristics of pregnant women with depressive symptoms identified by the Edinburgh Postnatal Depression Scale. Lydsdottir LB , Howard LM, Olafsdottir H, Thome M, Tyrfingsson P, Sigurdsson JF.	Unrelated
109.	Eur Eat Disord Rev. 2014 Mar;22(2):116-21. doi: 10.1002/erv.2269. Epub 2013 Nov 26. The clinical implications of high levels of autism spectrum disorder features in anorexia nervosa: a pilot study. Huke V , Turk J, Saeidi S, Kent A, Morgan JF.	No OCD
110.	Stereotact Funct Neurosurg. 2013;91(6):364-72. doi: 10.1159/000348278. Epub 2013 Oct 9. Treatment of intractable anorexia nervosa with inactivation	No OCD

	of the nucleus accumbens using stereotactic surgery. Wang J , Chang C, Geng N, Wang X, Gao G.	
111.	Encephale. 2014 Sep;40(4):323-9. doi: 10.1016/j.encep.2013.06.008. Epub 2013 Oct 1. [Triggers of bulimia and compulsion attacks: validation of the "Start" questionnaire]. [Article in French] Rigaud D , Jiang T(2), Pennacchio H(3), Brémont M(3), Perrin D(4).	No OCD
112.	J Pak Med Assoc. 2012 Dec;62(12):1340-1. A pica case confused with renal and bladder stones. Gulum M , Yeni E, Savas M, Sahin MA, Kati M.	Case-report
113.	Behav Res Ther. 2013 Aug;51(8):512-7. doi: 10.1016/j.brat.2013.05.007. Epub 2013 May 27. Personality-based subtypes of anorexia nervosa: examining validity and utility using baseline clinical variables and ecological momentary assessment. Lavender JM , Wonderlich SA, Crosby RD, Engel SG, Mitchell JE, Crow SJ, Peterson CB, Le Grange D.	No OCD
114.	J Psychiatr Res. 2013 Jun;47(6):747-54. doi: 10.1016/j.jpsychires.2012.12.015. Epub 2013 Jan 19. Common genetic background in anorexia nervosa and obsessive compulsive disorder: preliminary results from an association study. Mas S , Plana MT, Castro-Fornieles J, Gassó P, Lafuente A, Moreno E, Martinez E, Milà M, Lazaro L.	Lumping
115.	Cogn Behav Ther. 2013;42 :64-76. doi: 10.1080/16506073.2012.751124. Epub 2013 Jan 15. Treatment of obsessive-compulsive disorder complicated by comorbid eating disorders. Simpson HB , Wetterneck CT, Cahill SP, Steinglass JE, Franklin ME, Leonard RC, Weltzin TE, Riemann BC.	Unrelated
116.	Psychiatry Res. 2013 Sep 30;209(2):186-95. doi: 10.1016/j.psychres.2012.12.006. Epub 2013 Jan 5. Symptom dimensions, clinical course and comorbidity in men and women with obsessive-compulsive disorder. Torresan RC , Ramos-Cerqueira AT, Shavitt RG, do Rosário MC, de Mathis MA, Miguel EC, Torres AR.	Adult
117.	Child Adolesc Ment Health. 2012 Nov;17(4):246-251. doi:10.1111/j.1475 3588.2012.00651.x. Epub 2012 Feb 22. Computer-assisted assessment of obsessive-compulsive disorder in young people: a preliminary evaluation of the Development and Well-Being Assessment. Krebs G , Liang H , Hilton K , Macdiarmid F(2), Heyman I (3).	No ED
**118.**	**Eur Neuropsychopharmacol. 2012 Sep;22(9):615-24. doi: 10.1016/j.euroneuro.2011.12.006. Epub 2012 Jul 31.Olfactory function and alternation learning in eating disorders. Stein D , Gross-Isseroff R, Besserglick R, Ziv A, Mayer G, Yaroslavsky A, Toledano A, Voet H, Weizman A, Hermesh H.**	**Included**
119.	World Neurosurg. 2013 Sep-Oct;80(3-4):S29.e1-10. doi: 10.1016/j.wneu.2012.06.039. Epub 2012 Jun 25. Deep-brain stimulation for anorexia nervosa. Wu H , Van Dyck-Lippens PJ, Santegoeds R, van Kuyck K, Gabriëls L, Lin G, Pan G, Li Y, Li D, Zhan S, Sun B, Nuttin B.	No OCD
120.	Psychiatry Res. 2012 Dec 30;200(2-3):513-7. doi: 10.1016/j.psychres.2012.04.032. Epub 2012 Jun 15. Depression, anxiety and obsessive-compulsive symptoms in relation to nutritional status and	Adult

	outcome in severe anorexia nervosa. Mattar L , Thiébaud MR, Huas C, Cebula C, Godart N.	
121.	Behav Res Ther. 2012 Aug;50(7-8):449-56. doi: 10.1016/j.brat.2012.04.003. Epub 2012 Apr 28. Cognitive distortions and eating pathology: specificity of thought-shape fusion. Coelho JS , Baeyens C, Purdon C, Pitet A, Bouvard M.	Adult
122.	Camb Q Healthc Ethics. 2012 Jul;21(3):396-7. doi: 10.1017/S0963180112000138. The case: Starving for perfection. Mishra R.	Case report
123.	Eat Weight Disord. 2012 Dec;17(4):e226-33. doi: 10.3275/8272. Epub 2012 Feb 21. Orthorexia nervosa: a frequent eating disordered behavior in athletes. Segura-García C , Papaianni MC, Caglioti F, Procopio L, Nisticò CG, Bombardiere L, Ammendolia A, Rizza P, De Fazio P, Capranica L.	Adult
124.	Aust N Z J Psychiatry. 2012 Feb;46(2):118-31. doi: 10.1177/0004867411432071. The comorbidity between eating disorders and anxiety disorders: prevalence in an eating disorder sample and anxiety disorder sample. Swinbourne J , Hunt C, Abbott M, Russell J, St Clare T, Touyz S.	No OCD
125.	Australas Psychiatry. 2011 Dec;19(6):526-30. doi: 10.3109/10398562.2011.603328. Early onset eating disorders in male adolescents: a series of 10 inpatients. Bayes A , Madden S.	Unrelated
126.	Kobe J Med Sci. 2011 May 11;56(6):E263-9. Assessment of dissociation symptoms in patients with mental disorders by the Dissociation Questionnaire (DIS-Q). Matsui Y , Naito K, Matsuishi K, Kato H, Maeda K, Tanaka K.	Unrelated
127.	Seishin Shinkeigaku Zasshi. 2011;113(7):704-11. [The neurotic disorders]. [Article in Japanese] Honjo S , Nomura K, Kuriyama K, Suzuki F, Yoshikawa T.	No ED
128.	J Child Psychol Psychiatry. 2012 Jan;53 :64-72. doi: 10.1111/j.1469-7610.2011.02432.x. Epub 2011 Jul 28. Is sensory over-responsivity distinguishable from childhood behavior problems? A phenotypic and genetic analysis.Van Hulle CA , Schmidt NL, Goldsmith HH.	Unrelated
129.	Nervenarzt. 2011 Sep;82(9):1093-9. doi: 10.1007/s00115-010-3231-1. [Anorexia nervosa in childhood and adolescence: course and significance for adulthood]. [Article in German] Herpertz-Dahlmann B , Bühren K, Seitz J.	Unrelated
130.	Psychol Med. 2011 Dec;41(12):2507-13. doi: 10.1017/S003329171100078X. Epub 2011 Jun 7. Is childhood OCD a risk factor for eating disorders later in life? A longitudinal study. Micali N , Hilton K, Nakatani E, Heyman I, Turner C, Mataix-Cols D.	No ED
131.	Body Image. 2011 Jun;8(3):301-5. doi: 10.1016/j.bodyim.2011.04.007. Epub 2011 Jun 12. Measuring dysmorphic concern in Italy: psychometric properties of the Italian Body Image Concern Inventory (I-BICI). Luca M , Giannini M, Gori A, Littleton H.	Unrelated

132.	Asian J Psychiatr. 2011 Jun;4(2):150-1. doi: 10.1016/j.ajp.2011.03.001. Epub 2011 Jun 10. Psychiatric comorbidities in patients with celiac disease: Is there any concrete biological association? Sharma TR , Kline DB, Shreeve DF, Hartman DW.	Unrelated
133.	Int J Eat Disord. 2012 May;45(4):615-21. doi: 10.1002/eat.20936. Epub 2011 May 3. Fear of food as a treatment target: exposure and response prevention for anorexia nervosa in an open series. Steinglass J , Albano AM, Simpson HB, Carpenter K, Schebendach J, Attia E.	Unrelated
134.	Behav Cogn Psychother. 2011 Jul;39(4):457-70. doi: 10.1017/S1352465811000099. Epub 2011 Apr 4. Study of obsessive compulsive beliefs: relationship with eating disorders. Roncero M , Perpiñá C, García-Soriano G.	Lumping
135.	Psychiatr Pol. 2010 Sep-Oct;44(5):651-63. [Comorbidity in adolescence: simultaneous declaration of depressive, eating, obsessive-compulsive symptoms and use of psychoactive substances in the general population of 17 year old students in a big city]. [Article in Polish] Modrzejewska R .	Unrelated
136.	J Clin Psychol. 2011 May;67(5):507-16. doi: 10.1002/jclp.20794. Epub 2011 Mar 4. Compulsive hoarding in children. Storch EA , Rahman O, Park JM, Reid J, Murphy TK, Lewin AB.	No ED
137.	J Clin Psychol. 2011 Apr;67(4):391-403. doi: 10.1002/jclp.20770. Epub 2011 Jan 3. The significance of repetitive hair-pulling behaviors in eating disorders. Zucker N , Von Holle A, Thornton LM, Strober M, Plotnicov K, Klump KL, Brandt H, Crawford S, Crow S, Fichter MM, Halmi KA, Johnson C, Kaplan AS, Keel P, LaVia M, Mitchell JE, Rotondo A, Woodside DB, Berrettini WH, Kaye WH, Bulik CM.	Unrelated
138.	Int J Eat Disord. 2011 Nov;44(7):655-60. doi: 10.1002/eat.20902. Epub 2011 Feb 14. Compulsive exercise: the role of personality, psychological morbidity, and disordered eating. Goodwin H , Haycraft E, Willis AM, Meyer C.	No OCD
139.	Clin Psychol Psychother. 2010 Nov-Dec;17(6):519-27. doi: 10.1002/cpp.701. General psychopathology in anorexia nervosa: the role of psychosocial factors. Karatzias T , Chouliara Z, Power K, Collin P, Yellowlees A, Grierson D.	No OCD
140.	Compr Psychiatry. 2010 Nov-Dec;51(6):566-71. doi:10.1016/j.comppsych.2010.03.002. Epub 2010 Apr 24. Predictors of excessive exercise in anorexia nervosa. Bewell-Weiss CV , Carter JC.	No OCD
141.	Aust N Z J Psychiatry. 2010 Dec;44(12):1118-25. doi: 10.3109/00048674.2010.524625. Epub 2010 Oct 22. Facial affect recognition in anorexia nervosa: is obsessionality a missing piece of the puzzle? Castro L , Davies H, Hale L, Surguladze S, Tchanturia K.	Unrelated
142.	Psychiatr Pol. 2010 Jul-Aug;44(4):579-92. [Prevalence of mental disorders and psychoactive substance use in metropolitan 17-year old youth population]. [Article in Polish] Modrzejewska R , Bomba J.	Unrelated
143.	Neuropsychobiology. 2010 Aug;62(3):151-7. doi: 10.1159/000318571. Epub 2010 Jul 14. Preproenkephalin expression in peripheral blood mononuclear	Unrelated

	cells of acutely underweight and recovered patients with anorexia nervosa. Weiss D , Infante-Duarte C, Salbach Andrae H, Burghardt R, Hamann I, Pfeiffer E, Lehmkuhl U, Ehrlich S.	
144.	Eat Weight Disord. 2010 Mar-Jun;15(1-2):e86-9. doi: 10.1007/BF03325284. Typology of obsessive-compulsive symptoms in children and adolescents with anorexia nervosa. Hirani V , Serpell L, Willoughby K, Neiderman M, Lask B.	No Data
145.	J Psychiatr Res. 2010 Oct;44(14):910-6. doi: 10.1016/j.jpsychires.2010.02.011. Epub 2010 Apr 1. Mediation of symptom changes during inpatient treatment for eating disorders: the role of obsessive-compulsive features. Olatunji BO , Tart CD, Shewmaker S, Wall D, Smits JA.	Lumping
146.	Psychiatr Pol. 2009 Jul-Aug;43(4):457-68. [Application of group psychotherapy in treatment of adolescents and young women with anorexia nervosa (presentation of therapeutic work model in the Neurosis and Eating Disorders Treatment Centre "Dabrówka"--the author's own experience)]. [Article in Polish] Izydorczyk B , Niziołek E.	Unrelated
147.	Encephale. 2009 Dec;35(6):531-7. doi: 10.1016/j.encep.2008.09.005. [Social phobia in anorexia nervosa: evolution during the care]. [Article in French] Coulon N , Jeammet P, Godart N.	Unrelated
148.	J Korean Acad Nurs. 2009 Aug;39(4):459-68. doi: 10.4040/jkan.2009.39.4.459. [Impact of eating psychopathology, obsessive-compulsion and depression on self-harm behavior in patients with eating disorders]. [Article in Korean] Kong SS .	Lumping
149.	Depress Anxiety. 2009;26(11):1012-7. doi: 10.1002/da.20597. Early onset of obsessive-compulsive disorder and associated comorbidity. Janowitz D , Grabe HJ, Ruhrmann S, Ettelt S, Buhtz F, Hochrein A, Schulze-Rauschenbach S, Meyer K, Kraft S, Ferber C, Pukrop R, Freyberger HJ, Klosterkötter J, Falkai P, John U, Maier W, Wagner M.	No ED
150.	Am J Psychiatry. 2009 Jun;166(6):664-74. doi: 10.1176/appi.ajp.2009.08091354. Epub 2009 May 15. Functional disturbances within frontostriatal circuits across multiple childhood psychopathologies. Marsh R , Maia TV, Peterson BS.	Adult
151.	Int Clin Psychopharmacol. 2009 May;24(3):150-8. doi: 10.1097/YIC.0b013e328329c7b5. Lamotrigine in the treatment of binge-eating disorder with obesity: a randomized, placebo-controlled monotherapy trial. Guerdjikova AI , McElroy SL, Welge JA, Nelson E, Keck PE, Hudson JI.	Unrelated
152.	Psychiatr Danub. 2009 Mar;21 :68-71. Usher syndrome and psychiatric symptoms: a challenge in psychiatric management. Rijavec N , Grubic VN.	Unrelated
153.	Eat Disord. 2009 Mar-Apr;17(2):162-82. doi: 10.1080/10640260802714654. The role of depression and anxiety in impulsive and obsessive-compulsive behaviors among anorexic and bulimic patients. Finzi-Dottan R , Zubery E.	Adult

154.	J Am Diet Assoc. 2008 Dec;108(12):2105-8. doi: 10.1016/j.jada.2008.09.015. Refeeding syndrome: recognition is the key to prevention and management. Tresley J, Sheean PM.	Unrelated
155.	Br Dent J. 2008 Nov 22;205(10):525. doi: 10.1038/sj.bdj.2008.994. Wikipedia comes second. Stillman-Lowe C.	Unrelated
156.	CNS Spectr. 2008 Oct;13(10):906-10. doi: 10.1017/s1092852900017016. Nature against nurture: calcification in the right thalamus in a young man with anorexia nervosa and obsessive-compulsive personality disorder. Conrad R , Wegener I, Geiser F, Imbierowicz K, Liedtke R.	Case-report
157.	Int J Eat Disord. 2009 Apr;42(3):267-74. doi: 10.1002/eat.20605. The Yale-Brown-Cornell eating disorder scale in women with anorexia nervosa: what is it measuring? Jordan J , Joyce PR, Carter FA, McIntosh VV, Luty SE, McKenzie JM, Frampton CM, Bulik CM.	Unrelated
158.	Prog Neuropsychopharmacol Biol Psychiatry. 2008 Dec 12;32(8):2010-1. doi: 10.1016/j.pnpbp.2008.09.011. Epub 2008 Sep 27. Is Pica an eating disorder or an obsessive-compulsive spectrum disorder? Hergüner S, Ozyildirim I, Tanidir C.	Case-report
159.	Br Dent J. 2008 Sep 13;205(5):235-49. doi: 10.1038/sj.bdj.2008.734. Access to special care dentistry, part 6. Special care dentistry services for young people. Dougall A , Fiske J.	Unrelated
160.	Int J Eat Disord. 2009 Jan;42 :76-80. doi: 10.1002/eat.20579. Do obsessive compulsive symptoms mediate the relationship between maternal eating psychopathology and restrictive feeding practices? Farrow CV , Blissett J.	Unrelated
161.	Z Kinder Jugendpsychiatr Psychother. 2008 May;36(3):177-84. doi: 10.1024/1422-4917.36.3.177. [Mental disorders among relatives of patients with anorexia nervosa and bulimia nervosa]. [Article in German] Wagner A , Wöckel L, Bölte S, Radeloff D, Lehmkuhl G, Schmidt MH, Poustka F.	Unrelated
162.	Eat Weight Disord. 2008 Jun;13(2):e20-4. An adolescent male with features of EDNOS and obsessive-compulsive disorder. Yanover T , Thompson JK, Straub DM.	Case-report
163.	World J Biol Psychiatry. 2009;10(4 Pt 2):648-57. doi: 10.1080/15622970802043117.Is anorexia nervosa a neuropsychiatric developmental disorder? An illustrative case report. Kerbeshian J , Burd L.	Case report
164.	Eur Eat Disord Rev. 2008 Nov;16(6):451-62. doi: 10.1002/erv.870. The development of the childhood retrospective perfectionism questionnaire (CHIRP) in an eating disorder sample. Southgate L , Tchanturia K, Collier D, Treasure J.	No OCD
165.	Int J Eat Disord. 2008 Sep;41(6):498-504. doi: 10.1002/eat.20533. Do adolescents with eating disorder not otherwise specified or full-syndrome bulimia nervosa differ in clinical severity, comorbidity, risk factors, treatment outcome or cost? Schmidt U , Lee S, Dodge L, Frost S, Jenkins M, Johnson-Sabine E, Keville S, Murphy R, Robinson P, Winn S, Yi I.	No OCD

166.	Ugeskr Laeger. 2008 Mar 31;170(14):1158. [Severe anorexia nervosa: five fatal cases]. [Article in Danish] Nielsen J , Jeppesen NE, Brixen KT, Hørder K, Støving RK.	Case report
167.	Prog Neuropsychopharmacol Biol Psychiatry. 2008 Aug 1;32(6):1362-74. doi: 10.1016/j.pnpbp.2008.01.008. Epub 2008 Jan 17.A systematic review of off-label uses of memantine for psychiatric disorders. Zdanys K , Tampi RR.	Review
168.	Child Psychiatry Hum Dev. 2008 Sep;39(3):261-72. doi: 10.1007/s10578-007-0086-1. Epub 2007 Nov 7. Psychiatric comorbidities among female adolescents with anorexia nervosa. Salbach-Andrae H , Lenz K, Simmendinger N, Klinkowski N, Lehmkuhl U, Pfeiffer E.	No OCD
169.	Acta Paediatr. 2007 Dec;96(12):1853-4. doi: 10.1111/j.1651-2227.2007.00516.x. Epub 2007 Oct 11.Sponge eating: is it an obsessive compulsive disorder or an unusual form of pica? Gupta A , Rajput S, Maduabuchi G, Kumar P.	Unrelated
170.	J Paediatr Child Health. 2008 Jan;44(1-2):70-3. doi: 10.1111/j.1440-1754.2007.01235.x. Epub 2007 Oct 10. Anorexia, Maudsley and an impressive recovery: one family's story. [No authors listed]	Case report
171.	Int J Eat Disord. 2008 Jan;41 :15-21. doi: 10.1002/eat.20462. PANDAS anorexia nervosa--endangered, extinct or nonexistent? Puxley F , Midtsund M, Iosif A, Lask B.	Unrelated
172.	Int J Eat Disord. 2008 Jan;41 :47-56. doi: 10.1002/eat.20463. Specific and nonspecific comorbidity in anorexia nervosa. Jordan J , Joyce PR, Carter FA, Horn J, McIntosh VV, Luty SE, McKenzie JM, Frampton CM, Mulder RT, Bulik CM.	Unrelated
173.	Eur Psychiatry. 2007 Oct;22(7):455-61. doi: 10.1016/j.eurpsy.2007.06.001. Epub 2007 Aug 30. Psychiatric diagnoses and personality traits associated with disadvantageous decision-making. Jollant F , Guillaume S, Jaussent I, Bellivier F, Leboyer M, Castelnau D, Malafosse A, Courtet P.	Unrelated
174.	Int J Eat Disord. 2007 Nov;40 Suppl:S46-51. doi: 10.1002/eat.20429.The association of anxiety disorders and obsessive compulsive personality disorder with anorexia nervosa: evidence from a family study with discussion of nosological and neurodevelopmental implications. Strober M , Freeman R, Lampert C, Diamond J.	Case report
175.	Eat Behav. 2007 Aug;8(3):334-49. doi: 10.1016/j.eatbeh.2006.11.009. Epub 2006 Dec 5. Obligatory exercise and eating pathology in college females: replication and development of a structural model. Thome JL , Espelage DL.	Adult
176.	Eat Behav. 2007 Aug;8(3):305-10. doi: 10.1016/j.eatbeh.2006.11.006. Epub 2006 Dec 5. Cognitive content and process in eating-disordered patients with obsessive-compulsive features. Lawson R , Waller G, Lockwood R.	Unrelated
177.	Eat Disord. 2007 Mar-Apr;15(2):111-24. doi: 10.1080/10640260701190634. Treatment responses of inpatient eating disorder women with and without co-occurring obsessive-compulsive disorder. Cumella EJ , Kally Z, Wall AD.	Adult

178.	Braz J Psychiatry. 2007 Mar;29 :18-22. Validity and limitations of the Brazilian version of the Composite International Diagnostic Interview (CIDI 2.1). Quintana MI , Gastal FL, Jorge MR, Miranda CT, Andreoli SB.	Unrelated
179.	J Psychiatr Pract. 2007 Mar;13(2):65-71. doi: 10.1097/01.pra.0000265762.79753.88. Is anorexia nervosa a delusional disorder? An assessment of eating beliefs in anorexia nervosa. Steinglass JE , Eisen JL, Attia E, Mayer L, Walsh BT.	No OCD
180.	J Anxiety Disord. 2008;22(2):243-52. doi: 10.1016/j.janxdis.2007.01.015. Epub 2007 Feb 12. Characterizing the hoarding phenotype in individuals with OCD: associations with comorbidity, severity and gender.Wheaton M , Timpano KR, Lasalle-Ricci VH, Murphy D.	Unrelated
181.	Int J Eat Disord. 2007 May;40(4):381-5. doi: 10.1002/eat.20363. Obsessive-compulsive characteristics in women who have recovered from bulimia nervosa. Morgan JC , Wolfe BE, Metzger ED, Jimerson DC.	Adult
182.	Br J Clin Psychol. 2006 Sep;45(Pt 3):331-42. doi: 10.1348/014466505x53579. Obsessive-compulsive beliefs and magical ideation in eating disorders. Lavender A , Shubert I, de Silva P, Treasure J.	Unrelated
183.	Eur J Pediatr. 2007 Jun;166(6):623-4. doi: 10.1007/s00431-006-0282-1. Epub 2006 Sep 29. Pitfalls in the approach to pica. Fotoulaki M , Panagopoulou P, Efstratiou I, Nousia Arvanitakis S.	Unrelated
184.	Eat Behav. 2006 Aug;7(3):196-203. doi: 10.1016/j.eatbeh.2006.05.001. Epub 2006 Jun 21. Impulsiveness and lack of inhibitory control in eating disorders. Claes L , Nederkoorn C, Vandereycken W, Guerrieri R, Vertommen H.	Unrelated
185.	Psychiatr Pol. 2006 Jan-Feb;40 :83-97.[Anxiety disorders in children suffering from anorexia nervosa]. [Article in Polish]. Lucka I .	No English
186.	Psychiatr Pol. 2006 Jan-Feb;40 :65-74. [Review of certain conceptions on eating disorders. Suggestions on psychotherapy for women with anorexia and bulimia nervosa (the authors' own experience)]. [Article in Polish] Izydorczyk B , Czekaj B.	Review
187.	Psychosom Med. 2006 May-Jun;68(3):454-62. doi: 10.1097/01.psy.0000221254.77675.f5. Psychiatric comorbidities of female inpatients with eating disorders. Blinder BJ , Cumella EJ, Sanathara VA.	Lumping
188.	Int J Eat Disord. 2006 Dec;39(8):772-8. doi: 10.1002/eat.20274. Does the frequency of anxiety and depressive disorders differ between diagnostic subtypes of anorexia nervosa and bulimia? Godart N , Berthoz S, Rein Z, Perdereau F, Lang F, Venisse JL, Halfon O, Bizouard P, Loas G, Corcos M, Jeammet P, Flament M, Curt F.	Unrelated
189.	Psychiatry Res. 2006 Jun 15;142(2-3):209-17. doi: 10.1016/j.psychres.2005.11.006. Epub 2006 May 12. Anorexia nervosa with excessive exercise: a phenotype with close links to obsessive-compulsive disorder. Davis C , Kaptein S.	Adult

190.	Child Abuse Negl. 2006 Mar;30(3):257-69. doi: 10.1016/j.chiabu.2005.09.004. The impact of childhood sexual abuse in anorexia nervosa. Carter JC , Bewell C, Blackmore E, Woodside DB.	No OCD
191.	Eur Child Adolesc Psychiatry. 2006 Mar;15(2):111-7. doi: 10.1007/s00787-006-0510-2. The extent of social anxiety in combination with mental disorders. Melfsen S , Walitza S, Warnke A.	Unrelated
192.	Am J Psychiatry. 2006 Feb;163(2):327; author reply 327-9. Doi: 10.1176/appi.ajp.163.2.327. Comorbidity of anxiety with eating disorders and OCD. Södersten P, Bergh C.	Unrelated
193.	Child Adolesc Ment Health. 2006 Feb;11 :9-12. doi: 10.1111/j.1475-3588.2005.00348.x. Evidence Based Research in CBT with Adolescent Eating Disorders. Gowers SG .	No OCD
194.	Int J Eat Disord. 2005 Dec;38(4):371-4. doi: 10.1002/eat.20190. The relation among perfectionism, obsessive-compulsive personality disorder and obsessive-compulsive disorder in individuals with eating disorders. Halmi KA , Tozzi F, Thornton LM, Crow S, Fichter MM, Kaplan AS, Keel P, Klump KL, Lilenfeld LR, Mitchell JE, Plotnicov KH, Pollice C, Rotondo A, Strober M, Woodside DB, Berrettini WH, Kaye WH, Bulik CM.	Adult
195.	Encephale. 2005 May-Jun;31(3):279-88. doi: 10.1016/s0013-7006(05)82392-9. [Are anxiety or depressive disorders more frequent among one of the anorexia or bulimia nervosa subtype?]. [Article in French] Godart NT , Curt F, Perdereau F, Lang F, Venisse JL, Halfon O, Bizouard P, Loas G, Corcos M, Jeammet P, Flament MF.	No OCD
196.	Mol Psychiatry. 2005 Dec;10(12):1059-61. doi: 10.1038/sj.mp.4001728. Support for the association between the rare functional variant I425V of the serotonin transporter gene and susceptibility to obsessive compulsive disorder. Delorme R, Betancur C, Wagner M, Krebs MO, Gorwood P, Pearl P, Nygren G, Durand CM, Buhtz F, Pickering P, Melke J, Ruhrmann S, Anckarsäter H, Chabane N, Kipman A, Reck C, Millet B, Roy I, Mouren-Simeoni MC, Maier W, Råstam M, Gillberg C, Leboyer M, Bourgeron T.	No ED
197.	J Nerv Ment Dis. 2005 Aug;193(8):564-7. doi: 10.1097/01.nmd.0000172681.51661.54. Predictors of remission from body dysmorphic disorder: a prospective study. Phillips KA , Pagano ME, Menard W, Fay C, Stout RL.	No OCD
198.	Behav Neurol. 2005;16 :1-8. doi: 10.1155/2005/795343. D8/17 monoclonal antibody: an unclear neuropsychiatric marker. Morer A , Viñas O, Lázaro L, Bosch J, Toro J, Castro J.	Lumping
199.	Int J Eat Disord. 2005 Jul;38 :24-9. doi: 10.1002/eat.20140. Does "excessive" or "compulsive" best describe exercise as a symptom of bulimia nervosa? Adkins EC , Keel PK.	No OCD
200.	Eur Neuropsychopharmacol. 2006 Jan;16 :1-6. doi: 10.1016/j.euroneuro.2005.04.016. Epub 2005 Jun 6. The serotonin-1Dbeta receptor gene and severity of obsessive-compulsive disorder in women with bulimia nervosa. Levitan RD , Kaplan AS, Masellis M, Basile VS, Richter MA, Kennedy JL. @camh.net	Adult

201.	Prax Kinderpsychol Kinderpsychiatr. 2005 Apr;54(4):248-67. [Adolescent eating disorders]. [Article in German] Herpertz-Dahlmann B , Hagenah U, Vloet T, Holtkamp K.	No OCD
202.	Braz J Psychiatry. 2005 Mar;27 :25-31. doi: 10.1590/s1516-44462005000100008. Epub 2005 Apr 18. The Eating Disorders Section of the Development and Well-Being Assessment. (DAWBA): development and validation. Moya T , Fleitlich-Bilyk B, Goodman R, Nogueira FC, Focchi PS, Nicoletti M, Pinzon V, Cordás TA, Lotufo Neto F.	No OCD
203.	Int J Eat Disord. 2005 Apr;37(3):275-7. doi: 10.1002/eat.20136. Anorexia nervosa and obsessive-compulsive disorder in a prepubertal patient with bone dysplasia: a case report. Lázaro L , Moreno E, Baos P, Castro J.	Case report
204.	Eur Child Adolesc Psychiatry. 2005 Mar;14(2):106-10. doi: 10.1007/s00787-005-0431-5. Depression, anxiety, and obsessionality in long-term recovered patients with adolescent-onset anorexia nervosa. Holtkamp K , Müller B, Heussen N, Remschmidt H, Herpertz-Dahlmann B.	Unrelated
205.	Psychiatr Pol. 2004 Nov-Dec;38(6):1011-8. [Characteristics of the forming personality in children suffering from anorexia nervosa]. [Article in Polish] Lucka I , Cebella A.	No OCD
206.	J Psychiatr Res. 2005 May;39(3):303-10. doi: 10.1016/j.jpsychires.2004.08.001. A retrospective study of SSRI treatment in adolescent anorexia nervosa: insufficient evidence for efficacy. Holtkamp K , Konrad K, Kaiser N, Ploenes Y, Heussen N, Grzella I, Herpertz-Dahlmann B.	No OCD
207.	Am J Psychiatry. 2004 Dec;161(12):2215-21. doi: 10.1176/appi.ajp.161.12.2215. Comorbidity of anxiety disorders with anorexia and bulimia nervosa. Kaye WH , Bulik CM, Thornton L, Barbarich N, Masters K.	Adult
208.	J Clin Psychiatry. 2004 Nov;65(11):1463-9.doi:10.4088/jcp.v65n1104.Topiramate in the long-term treatment of binge-eating disorder associated with obesity.McElroy SL , Shapira NA,Arnold LM, Keck PE,Rosenthal NR,Wu SC,Capece JA,Fazzio L,Hudson JI.	No OCD
209.	Am J Med Genet B Neuropsychiatr Genet.2004 Nov 15;131B:7680. doi:10.1002/ajmg.b.20154.CAG repeat polymorphism within the KCNN3 gene is a significant contributor to susceptibility to anorexia nervosa a case-control study of female patients and several ethnic groups in the Israeli Jewish population. Koronyo-Hamaoui M, Gak E,Stein D,Frisch A,Danziger Y Leor S, Michaelovsky E, Laufer N, Carel C,Fennig S,Mimouni M, Apter A, Goldman B, Barkai G, Weizman A.	Unreleated
210.	J Psychiatr Res. 2004 Sep-Oct;38:545-52.doi:10.1016/j.jpsychires.2004.03.001.Set shifting in anorexia nervosa: an examination before and after weight gain,in full recovery and relationship to childhood and adult OCPD traits.Tchanturia K ,Morris RG, Anderluh MB, Collier DA, Nikolaou V,Treasure J.	Adult
211.	J Clin Exp Neuropsychol. 2004 Apr;26:190-9.doi:10.1076/jcen.26.2.190.28091. Conditional-associative learning in	Adult

	eating disorders: a comparison with OCD. Murphy R, Nutzinger DO, Paul T, Leplow B.	
212.	Arch Gen Psychiatry. 2004 Feb;61:192-200.doi:10.1001/archpsyc.61.2.192.Application of a latent class analysis to empirically define eating disorder phenotypes.Keel PK,Fichter M, Quadflieg N, Bulik CM, Baxter MG, Thornton L,Halmi KA, Kaplan AS, Strober M,Woodside DB, Crow SJ, Mitchell JE, Rotondo A, Mauri M, Cassano G, Treasure J, Goldman D, Berrettini WH, Kaye WH.	No OCD
213.	Z Kinder Jugendpsychiatr Psychother.2003 Aug;31:175-85.doi:10.1024/1422 4917.31.3.175. [Comorbidity of compulsive disorders in childhood and adolescence]. [Article in German] Becker K,Jennen-Steinmetz Ch,Holtmann M,el-Faddagh M, Schmidt MH.	No English
214.	Int J Eat Disord. 2003 Sep;34:211-9.doi:10.1002/eat.10177. Anxiety and psychoactive substance use disorder comorbidity in anorexia nervosa or depression.Jordan J,Joyce PR, Carter FA, Horn J, McIntosh VV, Luty SE, McKenzie JM, Mulder RT, Bulik CM.	No OCD
215.	Gen Hosp Psychiatry.2003 Jul-Aug;25:293-4.doi:10.1016/s0163-8343(03)00039-2.Is pica in the spectrum of obsessive-compulsive disorders? Gundogar D,Demir SB, Eren I.	Adult
216.	Arch Pediatr. 2003 Apr;10:337-9.doi:10.1016/s0929-693x(03)00332-4.[Choking phobia in childhood: a differential diagnosis with anorexia nervosa].[Article in French] Bailly D,de Chouly de Lenclave MB, Dhaussy S, Baert F,Turck D.	Unrelated
217.	J Affect Disord. 2003 Jul;75:181-9.doi:10.1016/s0165-0327(02)00038-.Depression and suicidal behavior in adolescent inpatients with obsessive compulsive disorder.Apter A, Horesh N, Gothelf D, Zalsman G, Erlich Z, Soreni N, Weizman A.	Unrelated
218.	Psychiatr Pol. 2003 Jan-Feb;37:29-37.[Anorexia nervosa in adult women].[Article in Polish] Rabe-Jabłońska J.	No OCD
219.	Int J Adolesc Med Health2002 Oct-Dec;14:329-34.doi:10.1515/ijamh.2002.14.4.329. Three case reports on the relationship between anorexia nervosa and obsessive compulsive disorder.Fisher M,Fornari V,Waldbaum R, Gold R.	Case Report
220.	Eur Child Adolesc Psychiatry.2003;12 Suppl1:I78-90.doi:10.1007/s00787-003-1111-y. Outcome of teenage-onset anorexia nervosa in a Swedish community-based sample. Råstam M, Gillberg C, Wentz E.	Adult
221.	Am J Psychiatry.2003 Feb;160:242-7.doi:10.1176/appi.ajp.160.2.242.Childhood obsessive-compulsive personality traits in adult women with eating disorders:defining a broader eating disorder phenotype.Anderluh MB,Tchanturia K, Rabe-Hesketh S,Treasure J.	Adult
222.	Prog Neuropsychopharmacol Biol Psychiatry.2003 Feb;27:85-102.doi:10.1016/s0278 5846(02)00338-x. Role of selective serotonin	Unrelated

	reuptake inhibitors in psychiatric disorders:aomprehensive review. Vaswani M,Linda FK,Ramesh S.	
223.	J Nerv Ment Dis.2002 Nov;190:746-51.doi:10.1097/00005053-200211000-00004. Dissociation in adolescent girls with anorexia: relationship to comorbid psychopathology. Farrington A, Waller G, Neiderman M, Sutton V, Chopping J,Lask B.	No OCD
224.	J Am Acad Child Adolesc Psychiatry.2002 Oct;41:1197-204.doi:10.1097/00004583 200210000-00009. Menstrual functioning and psychopathology in a county-wide population of highschool girls.Bisaga K,Petkova E, Cheng J, Davies M, Feldman JF, Whitaker AH.	Unrelated
225.	J Affect Disord.2002 May;69(1-3):141-8.doi:10.1016/s0165-0327(01)00304-4. Body dysmorphic disorder in outpatients with major depression. Nierenberg AA,Phillips KA, Petersen TJ, Kelly KE, Alpert JE, Worthington JJ,Tedlow JR, Rosenbaum JF, Fava M.	Unrelated
226.	J Clin Exp Neuropsychol.2002 Apr;24:176-86.doi:10.1076/jcen.24.2.176.990.Dissociated conditional-associative learning in anorexia nervosa. Murphy R, Nutzinger DO, Paul T, Leplow B.	No OCD
227.	Int J Eat Disord.2002 Apr;31:284-9.doi:10.1002/eat.10013. Comorbidity of obsessive-compulsive disorders and duration of eating disorders. Milos G , Spindler A, Ruggiero G, Klaghofer R, Schnyder U.	Adult
228.	Am J Psychiatry.2002 Mar;159 :408-11.doi:10.1176/appi.ajp.159.3.408. Self-injurious behavior in women with eating disorders. Paul T , Schroeter K, Dahme B, Nutzinger DO.	Unrelated
229.	Fortschr Neurol Psychiatr.2001 Aug;69:379-87.doi:10.1055/s-2001-16511.[Personality disorders and psychiatric comorbidity in obsessive-compulsive disorder and anorexia nervosa].[Article in German] Müller B , Wewetzer C, Jans T, Holtkamp K, Herpertz SC, Warnke A, Remschmidt H, Herpertz-Dahlmann B.	Adult
230.	Epidemiol Psichiatr Soc.2001 Apr-Jun;10:90-5.doi:10.1017/s1121189x00005169. [Post-partum as a specific risk factor for the onset of obsessive-compulsive disorder: clinical-controlled study]. [Article in Italian] Maina G,Vaschetto P,Ziero S, Di Lorenzo R, Bogetto F.	Unrelated
231.	Z Kinder Jugendpsychiatr Psychother. 2001 Aug;29 :206-20. doi:10.1024//1422 4917.29.3.206.[Evaluated treatment approaches in child and adolescent psychiatry II]. [Article in German] Baving L , Schmidt MH.	Unrelated
232.	J Child Psychol Psychiatry. 2001 Jul;42 :613-22. Ten-year follow-up of adolescent-onset anorexia nervosa: psychiatric disorders and overall functioning scales. Wentz E,Gillberg C, Gillberg IC, Råstam M.	No OCD
233.	Psychol Med. 2001 May;31 :635-45. doi: 10.1017/s0033291701003671. Deriving behavioural phenotypes in an international, multi-centre study of eating disorders. [No authors listed]	No OCD
234.	Psychopathology. 2001 May-Jun;34 :159-63. doi: 10.1159/000049300. Temperament and disruptive behavior disorders. Schmeck K , Poustka F.	Lumping

235.	Int J Eat Disord. 2001 May;29 :463-9. doi: 10.1002/eat.1043. Antibodies against human putamen in adolescents with anorexia nervosa. Harel Z , Hallett J, Riggs S, Vaz R, Kiessling L.	Unrelated
236.	Am J Psychiatry. 2001 Apr;158 :563-9. doi: 10.1176/appi.ajp.158.4.563. Morbidity risk for obsessive-compulsive spectrum disorders in first-degree relatives of patients with eating disorders. Bellodi L , Cavallini MC, Bertelli S, Chiapparino D, Riboldi C, Smeraldi E.	Adult
237.	Eat Weight Disord. 1998 Mar;3 :37-41. doi: 10.1007/BF03339985. Comorbidity of obsessive compulsive disorder in patients with eating disorders.Lennkh C , Strnad A, Bailer U, Biener D, Fodor G, de Zwaan M.	Adult
238.	Eat Weight Disord. 1999 Sep;4 :121-7. doi: 10.1007/BF03339727. Obsessive-compulsive symptoms as a correlate of severity in the clinical presentation of eating disorders: measuring the effects of depression. Speranza M , Corcos M, Levi G, Jeammet P.	Unrelated
239.	Eur Psychiatry. 2000 Aug;15 :302-5. doi: 10.1016/s0924-9338(00)00398-9. The links between body dysmorphic disorder and eating disorders. Rabe-Jablonska Jolanta J, Sobow Tomasz M.	No OCD
240.	J Child Adolesc Psychopharmacol. 2000 Summer;10 :133-45. doi: 10.1089/cap.2000.10.133. Infection-triggered anorexia nervosa in children:clinical description of four cases. Sokol MS .	Case Report
241.	J Am Acad Child Adolesc Psychiatry. 2000 Jul;39 :815-28. doi:10.1097/00004583-200007000-00010. Review of neuroimaging studies of child and adolescent psychiatric disorders from the past 10 years.Hendren RL,De Backer I, Pandina GJ.	Unrelated
242.	J Anxiety Disord. 2000 Jan-Feb;14 :19-30.doi:10.1016/s0887-6185(99)00027-4. Nosological insertion of axis I disorders in the etiology of obsessive-compulsive disorder.Yaryura-Tobias JA ,Grunes MS, Todaro J, McKay D, Neziroglu FA, Stockman R.	Lumping
243.	J Am Acad Child Adolesc Psychiatry. 1999 Nov;38 :1389-95. doi: 10.1097/00004583 199911000-00013. Ten-year follow-up of adolescent-onset anorexia nervosa: personality disorders. Nilsson EW , Gillberg C, Gillberg IC, Råstam M.	Lumping
244.	Compr Psychiatry. 1999 Sep-Oct;40 :337-42. doi: 10.1016/s0010-440x(99)90137-2. A comparison of clinical features among Japanese eating-disordered women with obsessive compulsive disorder. Matsunaga H , Miyata A, Iwasaki Y, Matsui T, Fujimoto K, Kiriike N.	Adult
245.	J Nerv Ment Dis. 1999 Jun;187 :336-42. doi: 10.1097/00005053-199906000-00002. Compulsive physical activity in adolescents with anorexia nervosa: a psychobehavioral spiral of pathology. Davis C , Katzman DK, Kirsh C.	Unrelated
246.	Behav Modif. 1999 Feb;23 :269-79. doi: 10.1177/0145445599232005. Assessment of obsessive-compulsive disorder. Emmelkamp PM , Kraaijkamp HJ, van den Hout MA.	No ED
247.	Acta Psychiatr Scand. 1999 Apr;99 :267-73. doi:10.1111/j.1600-0447.1999.tb07224.x. Low body mass indices in adolescents with	No ED

	obsessive-compulsive disorder. Henninghausen K , Rischmüller B, Heseker H, Remschmidt H, Hebebrand J.	
248.	J Am Acad Child Adolesc Psychiatry. 1999 Mar;38 :228-9. doi:10.1097/00004583-199903000-00002. Anorexia, OCD, and streptococcus. Henry MC, Perlmutter SJ, Swedo SE.	Unrelated
249.	Aust N Z J Psychiatry. 1998 Dec;32 :823-9. doi: 10.3109/00048679809073872. Familial bipolar disorder: preliminary results from the Otago Familial Bipolar Genetic Study. Edmonds LK , Mosley BJ, Admiraal AJ, Olds RJ, Romans SE, Silverstone T, Walsh AE.	Unrelated
250.	Ugeskr Laeger.1998 Dec 7;160(50):7285-90. [Indications for the use of serotonin uptake inhibitors in children and adolescents]. [Article in Danish] Thomsen H.	Unrelated
251.	Arch Gen Psychiatry. 1998 Jul;55 :603-10. doi: 10.1001/archpsyc.55.7.603. A controlled family study of anorexia nervosa and bulimia nervosa: psychiatric disorders in first-degree relatives and effects of proband comorbidity.Lilenfeld LR , Kaye WH, Greeno CG, Merikangas KR, Plotnicov K, Pollice C, Rao R, Strober M, Bulik CM, Nagy L.	Lumping
252.	Psychiatr Pol.1998 Mar-Apr;32 :155-63.[Liaison between body dysmorphic disorder and eating disorders]. [Article in Polish] Rabe-Jabłońska J.	Unrelated
253.	Seishin Shinkeigaku Zasshi. 1998;100 :92-112. [Obsessive-compulsive symptoms in children and pre-adolescents: clinical observation of inpatients]. [Article in Japanese] Minami T .	No ED
254.	Am J Psychiatry. 1998 Apr;155 :548-51. doi: 10.1176/ajp.155.4.548. Does fluoxetine augment the inpatient treatment of anorexia nervosa? Attia E , Haiman C, Walsh BT,Flater SR.	Unrelated
255.	Acta Psychiatr Scand. 1997 Aug;96:101-7. doi:10.1111/j.1600-0447.1997.tb09913.x. Eating disorders and antecedent anxiety disorders: a controlled study.Bulik CM,Sullivan PF, Fear JL, Joyce PR.	No OCD
256.	J Am Acad Child Adolesc Psychiatry.1997 Aug;36 :1128-33.doi:10.1097/00004583 199708000-00021.Case study: an infection-triggered, autoimmune subtype of anorexia nervosa.Sokol MS , Gray NS.	Case Report
257.	Psychiatr Pol.1997 Jul-Aug;31 :409-16.[Anorexia nervosa in a young man: a case report]. [Article in Polish] Małkiewicz-Borkowska M,Zechowski C.	Case Report
258.	Zh Nevrol Psikhiatr Im S S Korsakova. 1997;97 :16-9. [Obsessive-phobic disorders in anorexia and bulimia nervosa]. [Article in Russian] Tsivil'ko MA, Korkina MV, Briukhin AE, Bushenina SD, Lineva TIu.	Adult
259.	Int J Eat Disord. 1997 Jan;21 :83-7. doi:10.1002/(sici)1098-108x(199701)21:1<83::aid-eat10>3.0.co;2-p. Obsessive compulsive comorbidity in the dieting disorders.Thornton C , Russell J.	Adult
260.	S Afr Med J. 1996 Dec;86(12 Suppl):1615-7. A case of pica--neither a compulsion nor an impulse. Gangdev PS, Kariuki FN.	No OCD

261.	S Afr Med J. 1996 Dec;86(12 Suppl):1586-8, 1591-2. Pica and the obsessive-compulsive spectrum disorders. Stein DJ , Bouwer C, van Heerden B.	Case Report
262.	Med Clin (Barc). 1996 Jun 29;107 :169-74. [Clinical, psychological and biological variables in a group of 108 adolescent patients with anorexia nervosa].[Article in Spanish] Lázaro L , Toro J, Canalda G, Castro J, Martínez E, Puig J.	No English
263.	Biol Psychiatry.1996 Jun 1;39:966-9.doi:10.1016/0006-3223(95)00306-1.Comparison of obsessions and compulsions in patients with anorexia nervosa and obsessive compulsive disorder. Bastiani AM,Altemus M, Pigott TA, Rubenstein C, Weltzin TE, Kaye WH.	Adult
264.	J Behav Ther Exp Psychiatry.1996 Jun;27 :195-6.doi:10.1016/0005-7916(96)82613-9.Pica as obsessive-compulsive disorder.Luiselli JK.	Adult
265.	Actas Luso Esp Neurol Psiquiatr Cienc Afines. 1996 Mar-Apr;24 :67-73.[Clinical aspects of anorexia nervosa in males]. [Article in Spanish] de la Serna de Pedro I.	Adult
266.	Psychiatr Pol. 1996 Mar-Apr;30 :187-200. [Obsessive-compulsive disorder in girls with eating disorders]. [Article in Polish] Rabe-Jabłońska J .	Adult
267.	Int J Eat Disord. 1995 Dec;18 :375-9. doi:10.1002/1098-108x(199512)18:4<375::aid-eat2260180412>3.0.co;2-s.Obsessive-compulsive disorder, trichotillomania, and anorexia nervosa: a case report. Pryor TL , Martin RL, Roach N.	Case Report
268.	J Am Acad Child Adolesc Psychiatry. 1995 Dec;34 :1648-54. doi:10.1097/00004583-199512000-00015. Defense mechanisms in severe adolescent anorexia nervosa. Gothelf D,Apter A, Ratzoni G, Orbach I, Weizman R, Tyano S, Pfeffer C.	Unrelated
269.	J Psychiatr Res. 1995 Jul-Aug;29 :333-42. doi: 10.1016/0022-3956(95)00020-6. Obsessionality in eating-disorder patients: relationship to clinical presentation and two-year outcome. Zubieta JK , Demitrack MA, Fenick A, Krahn DD.	Adult
270.	Eur Child Adolesc Psychiatry. 1995 Jul;4 :165-74. doi: 10.1007/BF01980455. A clinical and phenomenological study of 185 Spanish adolescents with anorexia nervosa. Toro J , Nicolau R, Cervera M, Castro J, Blecua MJ, Zaragoza M, Toro A.	Lumping
271.	Aust N Z J Psychiatry. 1995 Mar;29 :114-7. doi: 10.3109/00048679509075899. Gender differences in obsessive compulsive disorder. Castle DJ , Deale A, Marks IM.	Unrelated
272.	Int J Eat Disord. 1995 Jan;17 :33-8. doi:10.1002/1098-108x(199501)17:1<33::aid eat2260170104>3.0.co;2-2. Self-mutilation, anorexia, and dysmenorrhea in obsessive compulsive disorder. Yaryura-Tobias JA , Neziroglu FA, Kaplan S.	Unrelated
273.	Psychother Psychosom Med Psychol. 1995 Jan;45 :8-15. [Compulsive symptoms in anorexia and bulimia nervosa]. [Article in German] Thiel A , Ohlmeier M, Jacoby GE, Schüssler G.	Adult

274.	Am J Psychiatry. 1995 Jan;152 :72-5. doi: 10.1176/ajp.152.1.72. Obsessive-compulsive disorder among patients with anorexia nervosa and bulimia nervosa. Thiel A , Broocks A, Ohlmeier M, Jacoby GE, Schüssler G.	Adult
275.	J Clin Psychiatry. 1995;56 Suppl 4:14-26; discussion 27. Kleptomania, compulsive buying, and binge-eating disorder. McElroy SL , Keck PE Jr, Phillips KA.	Unrelated
276.	Z Psychosom Med Psychoanal. 1995;41 :60-76. [Obsessive-compulsive symptoms in structural ego defects--a study exemplified by anorexia and bulimia nervosa]. [Article in German] Thiel A , Schüssler G.	Unrelated
277.	Compr Psychiatry. 1995 Jan-Feb;36 :70-6. doi: 10.1016/0010-440x(95)90101-z. Anorexia nervosa 6 years after onset: Part II. Comorbid psychiatric problems. Råstam M , Gillberg IC, Gillberg C.	Adult
278.	Compr Psychiatry. 1995 Jan-Feb;36 :61-9. doi: 10.1016/0010-440x(95)90100-a. Anorexia nervosa 6 years after onset: Part I. Personality disorders. Gillberg IC , Råstam M, Gillberg C.	Adult
279.	Psychol Med. 1994 Aug;24 :605-11. doi: 10.1017/s0033291700027756. Bulimia comorbidity in the general population and in the clinic. Bushnell JA , Wells JE, McKenzie JM, Hornblow AR, Oakley-Browne MA, Joyce PR.	No OCD
280.	Eur Child Adolesc Psychiatry. 1994 Jul;3 :138-158. doi: 10.1007/BF02720322. Obsessive-compulsive disorder in children and adolescents. A review of the literature. Thomsen PH .	Review
281.	Eur Child Adolesc Psychiatry. 1994 Apr;3 :82-96. doi: 10.1007/BF01977670. Obsessive-compulsive disorder in children and adolescents. A 6-22-year follow-up study. Clinical descriptions of the course and continuity of obsessive-compulsive symptomatology.Per Thomsen H.	Adult
282.	Compr Psychiatry. 1994 Mar-Apr;35 :145-8. doi: 10.1016/0010-440x(94)90060-u. Compulsive behaviors and obsessive-compulsive disorder (OCD): lack of a relationship between OCD, eating disorders, and gambling. Black DW , Goldstein RB, Noyes R Jr, Blum N.	Adult
283.	J Nerv Ment Dis. 1993 Aug;181 :520-1. doi: 10.1097/00005053-199308000-00011. A putaminal lesion in an adolescent with obsessive-compulsive disorder and atypical anorexia nervosa. Hebebrand J , Siemon P, Lutcke A, MariB G, Remschmidt H.	Unrelated
284.	J Adolesc Health.1993 Jul;14 :390-3.doi:10.1016/s1054-139x(08)80013-4.QT prolongation by isoproterenol in anorexia nervosa.Harris JP , Kreipe RE, Rossbach CN.14642.	Case Report
285.	Int J Eat Disord.1993 Jul;14 :43-8.doi:10.1002/1098-108x(199307)14:1<43::aid eat2260140106>3.0.co;2-7.Family environment of eating disordered and depressed adolescents.Thienemann M,Steiner H.	No OCD
286.	Harefuah.1993 Apr 15;124:477-9,527,526.[Anorexia nervosa and obsessive-compulsive disorder in a young Russian immigrant].[Article in Hebrew] Iancu I, Kikenzon L, Ratzoni G, Apter A.	Case Report

287.	Acta Neurochir Suppl (Wien).1993;58:34-5.doi:10.1007/978-3-7091-9297-9_7. Dorsomedial thalamotomy as a treatment for terminal anorexia: a report of two cases. Zamboni R,Larach V, Poblete M, Mancini R, Mancini H, Charlin V, Parr F, Carvajal C, Gallardo R.	Case Report
288.	Pediatr Ann.1992 Nov;21:769-74. doi:10.3928/0090-4481-19921101-12.The adolescent male with an eating disorder.Farrow JA.	Lumping
289.	Adolescence.1992 Summer;27(106):381-6.Predisposition factors in anorexia nervosa. Nagel KL, Jones KH.	No OCD
290.	Psychosomatics.1992 Spring;33:156-65.doi:10.1016/S0033-3182(92)71991-4.Body dysmorphic disorder. Diagnostic issues and related disorders.Hollander E,Neville D, Frenkel M, Josephson S, Liebowitz MR.	Adult
291.	Acta Paedopsychiatr.1992;55 :207-10.Psychometric measures in clarifying diagnosis in malnourished adolescents.Thienemann M , Steiner H.	Case Report
292.	J Clin Psychiatry.1991 Nov;52 :464-71.An open trial of fluoxetine in patients with anorexia nervosa.Kaye WH,Weltzin TE, Hsu LK, Bulik CM.	Unrelated
293.	Br J Psychiatry.1991 Jun;158:829-33. doi:10.1192/bjp.158.6.829.Adoption and eating disorders: a high-risk group? Holden NL .	Unrelated
294.	Br J Psychiatry.1991 Feb;158:260-3. doi:10.1192/bjp.158.2.260.Gender-divergent aetiological factors in obsessive-compulsive disorder.Noshirvani HF, Kasvikis Y, Marks IM, Tsakiris F, Monteiro WO.	No ED
295.	Psychiatr Clin North Am. 1990 Sep;13 :469-88.Adolescence and eating disorder: the obsessive-compulsive syndrome.Rothenberg A .	Lumping
296.	Arch Gen Psychiatry.1990 May;47 :487-96.doi:10.1001/archpsyc.1990.01810170087013. Uncommon troubles in young people: prevalence estimates of selected psychiatric disorders in a nonreferred adolescent population.Whitaker A , Johnson J, Shaffer D, Rapoport JL, Kalikow K, Walsh BT, Davies M, Braiman S, Dolinsky A.	Unrelated
297.	Encephale.1989 May-Jun;15 :355-8.[Anorexia nervosa. A frequent antecedent of obsessive compulsive disorder].[Article in French] Zribi S, Chambon O, Cottraux J.	Adult
298.	Jpn J Psychiatry Neurol.1988 Jun;42:209-16.doi:10.1111/j.1440-1819.1988.tb01972.x. The perimenarche syndrome (a proposal).Ushijima S , Kobayashi R.	Unrelated
299.	J Child Psychol Psychiatry.1986 May;27 :289-95.doi:10.1111/j.1469-7610.1986.tb01833.x. Childhood obsessive compulsive disorder.Rapoport JL.	No ED
300.	Hosp Community Psychiatry.1983 Jun;34 :514-21.doi:10.1176/ps.34.6.514.Current issues in adolescent psychiatry. Hodgman CH.	Lumping
301.	Can J Psychiatry.1982 Jun;27 :282-6.doi:10.1177/070674378202700404.A comparative psychometric study of anorexia nervosa and obsessive neurosis.Solyom L, Freeman RJ, Miles JE.	Adult

302.	Br J Med Psychol.1977 Dec;50 :375-80.doi:10.1111/j.2044-8341.1977.tb02436.x.Some psychological characteristics of patients with anorexia nervosa whose weight has been newly restored.Pillay M, Crisp AH.	No OCD
303.	Tijdschr Ziekenverpl.1976 Nov 30;29 :1139-41.[Anorexia nervosa].[Article in Dutch] Luitjes WF.	No OCD
304.	Compr Psychiatry.1976 Jul-Aug;17 :527-39.doi: 10.1016/0010-440x(76)90035-3. Obsessive-compulsive neurosis: record, follow-up, and family studies.I. Inpatient record study.Welner A, Reich T, Robins E, Fishman R, Van Doren T.	No ED
305.	Br J Psychiatry.1976 Jan;128:57-60.doi:10.1192/bjp.128.1.57. Some personality characteristics of patients with anorexia nervosa. Smart DE, Beumont PJ, George GC.	Adult
306.	J Nerv Ment Dis.1974 Mar;158 :222-5.doi:10.1097/00005053-197403000-00008. Comparison of demographic and clinical features in patient groups with different ages and weights at onset of anorexia nervosa. Halmi KA.	No OCD
307.	Psychosom Med.1974 Jan-Feb;36:18-26. doi:10.1097/00006842-197401000-00002. Anorexia nervosa: demographic and clinical features in 94 cases. Halmi KA.	No OCD
308.	Proc R Soc Med.1973 Sep;66 :847-50.A psychiatrist's view of problems in adolescence. Reinhold M.	Unrelated
309.	S Afr Med J.1973 Sep 1;47(34):1544-50.[The psychodynamics of anorexia nervosa]. [Article in Afrikaans].Robbertze JH.	No OCD
310.	Br J Psychiatry.1969 Dec;115(529):1437-41.doi:10.1192/bjp.115.529.1437.A six year follow-up study of sixty-five adolescent patients:predictive value of presenting clinical picture.King LJ, Pittman GD.	Unrelated
311.	Schweiz Arch Neurol Neurochir Psychiatr.1968;101:383-93.[Anorexia nervosa in the male].[Article in German] Ladewig D.	No OCD
312.	Int J Psychoanal.1968;49 :682-90.Trichotillomania, trichophagy, and cyclic vomiting.A contribution to the psychopathology of female sexuality.Sperling M.	Unrelated
	Search from CENTRAL	
1	Validation of the yale-brown obsessive compulsive scale modified for binge eating LS Deal, RJ Wirth, M Gasior, BK Herman, SL McElroy International journal of eating disorders, 2015, 48(7), 994-1004 | added to CENTRAL: 31 gennaio 2017 | 2017 Issue 1	No OCD, duplicate
2	Cognitive remediation therapy (CRT) as a treatment enhancer of eating disorders and obsessive compulsive disorders: study protocol for a randomized controlled trial B van Passel, U Danner, A Dingemans, E van Furth, L Sternheim, A van Elburg, A van Minnen, M van den Hout, GJ Hendriks, D Cath BMC psychiatry, 2016, 16(1), 393 | added to CENTRAL: 30 giugno 2017 | 2017 Issue 6	Adult, duplicate

3	Perfectionism, anorexia nervosa, and family treatment: how perfectionism changes throughout treatment and predicts outcomes HA Welch, WS Agras, J Lock, KA Halmi International journal of eating disorders, 2020, 53(12), 2055-2060 | added to CENTRAL: 30 novembre 2020 | 2020 Issue 11	No OCD
4	Prevalence, correlates and comorbidities of feeding and eating disorders in a nationally representative sample of Iranian children and adolescents MR Mohammadi, SA Mostafavi, Z Hooshyari, A Khaleghi, N Ahmadi, P Molavi, A Armani Kian, P Safavi, A Delpisheh, S Talepasand, SK Hojjat, P Pourdehghan, R Ostovar, SH Hosseini, S Mohammadzadeh, M Salmanian, SS Alavi, A Ahmadi, H Zarafshan. International journal of eating disorders, 2020, 53(3), 349-361 | added to CENTRAL: 31 dicembre 2019 | 2019 Issue 12	No OCD, duplicate
5	Evaluation of Intravenous Immunoglobulin in Pediatric Acute-Onset Neuropsychiatric Syndrome. I Melamed, RH Kobayashi, M O'Connor, AL Kobayashi, A Schechterman, M Heffron, S Canterberry, H Miranda, N Rashid Journal of child and adolescent psychopharmacology, 2021, 31(2), 118-128 | added to CENTRAL: 30 novembre 2021 | 2021 Issue 11	Unrelated
6	Comparison of 2 family therapies for adolescent anorexia nervosa: a randomized parallel trial. WS Agras, J Lock, H Brandt, SW Bryson, E Dodge, KA Halmi, B Jo, C Johnson, W Kaye, D Wilfley, B Woodside. JAMA psychiatry, 2014, 71(11), 1279-1286 | added to CENTRAL: 30 aprile 2015 | 2015 Issue 4	No OCD
7	Randomized Controlled Trial Comparing Health Coach-Delivered Smartphone-Guided Self-Help With Standard Care for Adults With Binge Eating T Hildebrandt, A Michaeledes, M Mayhew, R Greif, R Sysko, T Toro-Ramos, L DeBar. American journal of psychiatry, 2020, 177(2), 134-142 | added to CENTRAL: 31 marzo 2020 | 2020 Issue 03	Unrelated, duplicate
8	Can adaptive treatment improve outcomes in family-based therapy for adolescents with anorexia nervosa? Feasibility and treatment effects of a multi-site treatment study. J Lock, D Le Grange, WS Agras, KK Fitzpatrick, B Jo, E Accurso, S Forsberg, K Anderson, K Arnow, M Stainer. Behaviour research and therapy, 2015, 73, 90-95 | added to CENTRAL: 30 novembre 2015 | 2015 Issue 11	No OCD
9	Reliability and Validity of the Schedule for Affective Disorders and Schizophrenia for School-Age Children-Present and Lifetime Version, DSM-5 November 2016-Turkish Adaptation (K-SADS-PL-DSM-5-T). F Unal, F Oktem, F Cetin Cuhadaroglu, SE Cengel Kultur, D Akdemir, D Foto Ozdemir, HT Cak, D Unal, K Tiras, C Aslan, BM Kalayci, BS Aydos, F Kutuk, E Tasyurek, R Karaokur, B Karabucak, B Karakok, Y Karaer, A Artik. Turk psikiyatri dergisi [Turkish journal of psychiatry], 2019, 30(1), 42-50 | added to CENTRAL: 31 agosto 2019 | 2019 Issue 08	Unrelated
10	Guided self-help interventions for mental health disorders in children with neurological conditions: study protocol for a pilot randomised controlled trial. S Bennett, I Heyman, A Coughtrey, J Simmonds, S Varadkar, T Stephenson, M DeJong, R Shafran. Trials, 2016, 17(1), 532 | added to CENTRAL: 28 febbraio 2018 | 2018 Issue 2	Unrelated, duplicate.
11	A comparison of short- and long-term family therapy for adolescent anorexia nervosa. J Lock, WS Agras, S Bryson, HC Kraemer. Journal of the American	No OCD

	Academy of Child and Adolescent. Psychiatry, 2005, 44(7), 632-639 | added to CENTRAL: 31 gennaio 2006 | 2006 Issue 1	
12	A pragmatic randomised multi-centre trial of multifamily and single family therapy for adolescent anorexia nervosa. I Eisler, M Simic, J Hodsoll, E Asen, M Berelowitz, F Connan, G Ellis, P Hugo, U Schmidt, J Treasure, I Yi, S Landau. BMC psychiatry, 2016, 16(1), 422 | added to CENTRAL: 30. giugno 2017 | 2017 Issue 6	No OCD
13	Successful growth hormone(GH)treatment for severe growth failure in adolecents with anorexia nervosa until adult height. AM Paulsen, A Bargiacchi, C Doyen, C Raverdy, J-C Carel, M-F Le Heuzey, J Leger. Endocrine reviews, 2015, 36 | added to CENTRAL: 28 febbraio 2017 | 2017 Issue 2	No OCD
14	Internet-based cognitive behavioral therapy in children and adolescents with obsessive-compulsive disorder: a randomized controlled trial. K Hollmann, CS Hohnecker, A Haigis, AK Alt, J Kuhnhausen, A Pascher, U Worz, R App, H Lautenbacher, TJ Renner, A Conzelmann. Frontiers in psychiatry, 2022, 13, 989550 | added to CENTRAL: 30 novembre 2022 | 2022 Issue 11	No ED
15	Optimal treatment for obsessive compulsive disorder: a randomized controlled feasibility study of the clinical-effectiveness and cost-effectiveness of cognitive-behavioural therapy, selective serotonin reuptake inhibitors and their combination in the management of obsessive compulsive disorder. NA Fineberg, DS Baldwin, LM Drummond, S Wyatt, J Hanson, S Gopi, S Kaur, J Reid, V Marwah, RA Sachdev, I Pampaloni, S Shahper, Y Varlakova, D Mpavaenda, C Manson, C O'Leary, K Irvine, D Monji-Patel, A Shodunke, T Dyer, A Dymond, G Barton, D Wellsted. International clinical psychopharmacology, 2018, 33(6), 334-348 | added to CENTRAL: 30 novembre 2018 | 2018 Issue 11	No ED
16	Polyunsaturated omega-3 fatty acids are not effective for depressive and compulsive symptoms in adolescent girls with anorexia nervosa. A Pirog-Balcerzak, A Bazynska, J Bragoszewska, P Niwinski, L Popek, B Remberk, F Rybakowski. European neuropsychopharmacology, 2016, 26, S729-S730 | added to CENTRAL: 28 febbraio 2017 | 2017 Issue 2	Lumping
17	A clinical study of esucos in a mentally retarded clientele. O Lindholm. Acta neurologica et psychiatrica Belgica, 1967, 67, Suppl:49-64 | added to CENTRAL: 31 gennaio 1998 | 1998 Issue 1	Unrelated
18	DSM-5 field trials in the United States and Canada, part II: test-retest reliability of selected categorical diagnoses. DA Regier, WE Narrow, DE Clarke, HC Kraemer, SJ Kuramoto, EA Kuhl, DJ Kupfer. American journal of psychiatry, 2013, 170(1), 59-70 | added to CENTRAL: 31 gennaio 2014 | 2014 Issue 1	Unrelated
19	Universal parent training as a supplement to inpatient psychiatric treatment for children and adolescents. C Schwenck, W Schneider, A Reichert. European child and adolescent psychiatry. 25 (8) (pp 879-889), 2016. Date of publication: 01 aug 2016., 2016 | added to CENTRAL: 31 ottobre 2016 | 2016 Issue 10	Unrelated
20	The Yale-Brown-Cornell eating disorder scale in women with anorexia nervosa: what is it measuring?. J Jordan, PR Joyce, FA Carter, VV McIntosh, SE Luty, JM McKenzie, CM Frampton, CM Bulik. International journal of eating	Adult, duplicate

	disorders, 2009, 42(3), 267-274 | added to CENTRAL: 31 ottobre 2009 | 2009 Issue 4	
21	Anxiety and psychoactive substance use disorder comorbidity in anorexia nervosa or depression. J Jordan, PR Joyce, FA Carter, J Horn, VV McIntosh, SE Luty, JM McKenzie, RT Mulder, CM Bulik International journal of eating disorders, 2003, 34(2), 211-219 | added to CENTRAL: 31 luglio 2004 | 2004 Issue 3	Adult, duplicate
22	Does fluoxetine augment the inpatient treatment of anorexia nervosa? E Attia, C Haiman, BT Walsh, SR Flater. American journal of psychiatry, 1998, 155(4), 548-551 | added to CENTRAL: 31 luglio 2009 | 2009 Issue 3	No OCD, duplicate
23	In-person vs. Ehealth mindfulness-based intervention for adolescents with chronic illnesses: a pilot randomized trial. N Chadi, E Weisbaum, C Malboeuf-Hurtubise, SA Kohut, C Viner, N Palaniyar, M Kaufman, J Locke, DX Vo. Adolescent psychiatry, 2019, 9(1), 11-23 | added to CENTRAL: 31 agosto 2019 | 2019 Issue 08	Lumping
24	Childhood-, teenage-, and adult-onset depression: diagnostic and individual characteristics in a clinical sample. K Fernando, JD Carter, CM Frampton, SE Luty, J McKenzie, RT Mulder, PR Joyce Comprehensive psychiatry, 2011, 52(6), 623-629 | added to CENTRAL: 31 marzo 2019 | 2019 Issue 3	Unrelated
25	Protocol for READY2Exit: a patient-oriented, mixed methods study examining transition readiness in adolescents with co-occurring physical and mental health conditions. B Allemang, S Samuel, KC Sitter, SB Patten, M Patton, K Pintson, K Greer, K Schofield, M Farias, Z Punjwani, AS Mackie, G Dimitropoulos. Journal of transition medicine, 2022, 4(1) | added to CENTRAL: 30 novembre 2022 | 2022 Issue 11	Lumping
26	Digital cognitive behavioral therapy for insomnia for adolescents with mental health problems: feasibility open trial. B Cliffe, A Croker, M Denne, J Smith, P Stallard. JMIR mental health, 2020, 7(3) | added to CENTRAL: 31 gennaio 2021 | 2021 Issue 01	Unrelated
27	Effects of institutional rearing and foster care on psychopathology at age 12 years in Romania: follow-up of an open, randomised controlled trial. KL Humphreys, MM Gleason, SS Drury, D Miron, CA Nelson, NA Fox, CH Zeanah. The lancet. Psychiatry, 2015, 2(7), 625-634 | added to CENTRAL: 30 settembre 2015 | 2015 Issue 9	Unrelated
28	Moderators and processes of change in a pilot randomized controlled trial of acceptance-enhanced behavior therapy for trichotillomania in adolescents. JM Petersen, LK Capel, ME Levin, MP Twohig. Journal of obsessive-compulsive and related disorders, 2022, 35 | added to CENTRAL: 30 novembre 2022 | 2022 Issue 11	Unrelated
29	Association of insulin-manipulation and psychiatric disorders: a systematic epidemiological evaluation of adolescents with type 1 diabetes in Austria. G Berger, T Waldhoer, I Barrientos, D Kunkel, BM Rami-Merhar, E Schober, A Karwautz, G Wagner. Pediatric diabetes, 2019, 20(1), 127-136 | added to CENTRAL: 30 novembre 2018 | 2018 Issue 11	Unrelated

30	Intranasal oxytocin in the treatment of anorexia nervosa: randomized controlled trial during re feeding. J Russell, S Maguire, GE Hunt, A Kesby, A Suraev, J Stuart, J Booth, IS McGregor Psychoneuroendocrinology, 2018, 87, 83-92 | added to CENTRAL: 30 novembre 2017 | 2017 Issue 11	No OCD
31	Ongoing or previous mental disorders predispose to adverse mood reporting during combined oral contraceptive use. H Bengtsdotter, C Lundin, K Gemzell Danielsson, M Bixo, J Baumgart, L Marions, J Brynhildsen, A Malmborg, I Lindh, I Sundström Poromaa. European journal of contraception & reproductive health care, 2018, 23(1), 45-51 | added to CENTRAL: 30 aprile 2018 | 2018 Issue 4	Unrelated

Supplementary Table 2. Risk of bias assessment

	Study	Risk of bias	Level
	Stein et al., 2012	Selection	Low
			Medium
			High
		Performance	Low
			Medium
			High
		Detection	Low
			Medium
			High
		Attrition	Low
			Medium
			High
		Reporting	Low
			Medium
			High
	Breithaupt et al., 2014	Selection	Low
			Medium
			High
		Performance	Low
			Medium
			High
		Detection	Low
			Medium
			High
		Attrition	Low
			Medium
			High
		Reporting	Low
			Medium
			High
	Brooks et al., 2014	Selection	Low

			Medium
			High
		Performance	Low
			Medium
			High
		Detection	Low
			Medium
			High
		Attrition	Low
			Medium
			High
		Reporting	Low
			Medium
			High
	Lewis et al., 2018	Selection	Low
			Medium
			High
		Performance	Low
			Medium
			High
		Detection	Low
			Medium
			High
		Attrition	Low
			Medium
			High
		Reporting	Low
			Medium
			High
	Tyszkiewicz-Nwafor et al., 2018	Selection	Low
			Medium
			High
		Performance	Low
			Medium
			High
		Detection	Low
			Medium
			High
		Attrition	Low
			Medium
			High
		Reporting	Low
			Medium
			High
	Plana et al., 2019	Selection	Low
			Medium
			High
		Performance	Low
			Medium
			High
		Detection	Low

			Medium
			High
		Attrition	Low
			Medium
			High
		Reporting	Low
			Medium
			High
	Flamarique et al., 2019	Selection	Low
			Medium
			High
		Performance	Low
			Medium
			High
		Detection	Low
			Medium
			High
		Attrition	Low
			Medium
			High
		Reporting	Low
			Medium
			High
	Bohon et al., 2020	Selection	Low
			Medium
			High
		Performance	Low
			Medium
			High
		Detection	Low
			Medium
			High
		Attrition	Low
			Medium
			High
		Reporting	Low
			Medium
			High
	Reilly et al., 2022	Selection	Low
			Medium
			High
		Performance	Low
			Medium
			High
		Detection	Low
			Medium
			High
		Attrition	Low
			Medium
			High
		Reporting	Low

			Medium
			High
	Camprodon-Boadas et al., 2023	Selection	Low
			Medium
			High
		Performance	Low
			Medium
			High
		Detection	Low
			Medium
			High
		Attrition	Low
			Medium
			High
		Reporting	Low
			Medium
			High
